# TEAD–YAP Interaction Inhibitors and MDM2 Binders from DNA‐Encoded Indole‐Focused Ugi Peptidomimetics

**DOI:** 10.1002/anie.202006280

**Published:** 2020-07-15

**Authors:** Verena B. K. Kunig, Marco Potowski, Mohammad Akbarzadeh, Mateja Klika Škopić, Denise dos Santos Smith, Lukas Arendt, Ina Dormuth, Hélène Adihou, Blaž Andlovic, Hacer Karatas, Shabnam Shaabani, Tryfon Zarganes‐Tzitzikas, Constantinos G. Neochoritis, Ran Zhang, Matthew Groves, Stéphanie M. Guéret, Christian Ottmann, Jörg Rahnenführer, Roland Fried, Alexander Dömling, Andreas Brunschweiger

**Affiliations:** ^1^ TU Dortmund University Faculty of Chemistry and Chemical Biology Otto-Hahn-Strasse 6 44227 Dortmund Germany; ^2^ Max Planck Institute of Molecular Physiology Department of Chemical Biology Otto-Hahn-Strasse 11 44227 Dortmund Germany; ^3^ TU Dortmund University Faculty of Statistics Vogelpothsweg 87 44227 Dortmund Germany; ^4^ Medicinal Chemistry, Research and Early Development, Cardiovascular, Renal and Metabolism (CVRM) BioPharmaceuticals R&D AstraZeneca 43150 Gothenburg Sweden; ^5^ AstraZeneca-Max Planck Institute Satellite Unit Max-Planck Institute of Molecular Physiology Department of Chemical Biology Otto-Hahn-Strasse 11 44227 Dortmund Germany; ^6^ Lead Discovery Center GmbH (Germany) Otto-Hahn-Strasse 15 44227 Dortmund Germany; ^7^ Laboratory of Chemical Biology Department of Biomedical Engineering and Institute for Complex Molecular Systems Eindhoven University of Technology Den Dolech 2 5612 AZ Eindhoven The Netherlands; ^8^ University of Groningen Drug Design Deusinglaan 1 7313 AV Groningen The Netherlands; ^9^ University of Crete Department of Chemistry 70013 Heraklion Greece

**Keywords:** combinatorial chemistry, DNA-encoded library, peptidomimetics, protein–protein interaction inhibition, Ugi reaction

## Abstract

DNA‐encoded combinatorial synthesis provides efficient and dense coverage of chemical space around privileged molecular structures. The indole side chain of tryptophan plays a prominent role in key, or “hot spot”, regions of protein–protein interactions. A DNA‐encoded combinatorial peptoid library was designed based on the Ugi four‐component reaction by employing tryptophan‐mimetic indole side chains to probe the surface of target proteins. Several peptoids were synthesized on a chemically stable hexathymidine adapter oligonucleotide “hexT”, encoded by DNA sequences, and substituted by azide‐alkyne cycloaddition to yield a library of 8112 molecules. Selection experiments for the tumor‐relevant proteins MDM2 and TEAD4 yielded MDM2 binders and a novel class of TEAD‐YAP interaction inhibitors that perturbed the expression of a gene under the control of these Hippo pathway effectors.

## Graphical abstract

**A focused approach**: A DNA‐encoded peptoid library was designed by the Ugi multicomponent reaction around indole structures that mimic the side chain of tryptophan. Applying this focused library to the challenging cancer targets MDM2 and hTEAD4 yielded compounds for inhibitor development. Compounds binding to hTEAD4 disrupted the hTEAD4–YAP interaction, and reduced expression of a gene under control of the TEAD–YAP transcription factor complex.

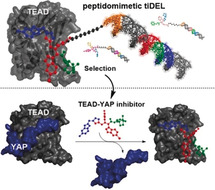

The development of small molecules that inhibit protein–protein interactions (PPIs) often suffers from a lack of starting points for compound design, even though many target proteins contain small‐molecule binding sites.^[1]^ PPI inhibitors harbor vast potential to understand biological systems and for drug development.^[2]^ For instance, PPIs such as the MDM2–p53 and TEAD–YAP interactions are involved in malignant diseases.^[1]^ Dysregulated PPIs of transcriptional enhancer factor‐1 domains (TEAD1‐4) with co‐transcription factor YAP (Yes‐associated protein), late Hippo signaling effectors, are involved in important oncogenic mechanisms.[^3^, ^4^, ^5^] Inhibition of the TEAD–YAP PPI has been achieved in vitro with peptides that addressed “interface 3” (Figure 1 a).[^6^, ^7^] In silico small‐molecule screening yielded TEAD–YAP inhibitors **1** and **2** (Figure 1 b).^[8]^ Intriguingly, TEAD is palmitoylated in a cavity, called a “central pocket”, which contributes to protein stability.^[9]^ Compounds that bound to this pocket such as niflumic acid (**4**), flufenamic acid (**5**), and TED‐347 (**6**) displaced a YAP‐derived peptide from hTEAD4, while quinolinol **7** augmented YAP–TEAD activity (Figure 1 c).^[10]^ DNA‐encoded libraries (DELs) have delivered a few PPI inhibitors.[^11^, ^12^] They enable deep sampling of chemical space around key or “anchor” motifs. Here, we designed a DNA‐encoded peptidomimetic library focused on the tryptophan side‐chain motif—the indole moiety (Figure 1 d). Tryptophan is significantly enriched in protein–protein interactions and often contributes disproportionally to protein binding.^[13]^ Thus, it has been exploited as an “anchor motif” for the design of PPI inhibitors.[^14^, ^15^] In this strategy, the anchor analogue—a substructure that is a chemical mimic of a specific amino acid residue (here tryptophan)—will be used to provide focused libraries with an increased probability of bioactivity. The anchor motif strategy has been repeatedly used to discover potent PPI inhibitors.^[16]^ Our DNA‐encoded peptidomimetic library was selected on the p53‐binding domain of MDM2 as the archetypal target for indole‐based peptidomimetics.^[14]^ As a second promising target for this library we selected the YAP‐interacting domain of (human) hTEAD4, because it contains a key tryptophan‐binding site in “interface 3”, and its central pocket has been demonstrated to accommodate heteroaromatic structures (Figure 1 a).[^10^, ^16^]

**Figure 1:**
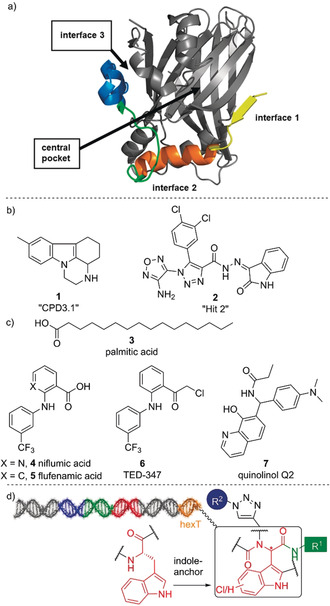
Targeting the TEAD family of transcription factors. a) Structure of the TEAD1–YAP complex (PDB ID: 3KYS; TEAD1 in gray, YAP in yellow, orange, green, and blue) with areas highlighted that can be targeted for inhibitor development. b) TEAD inhibitors binding to the surface of TEAD. c) TEAD modulators binding to a palmitate‐accommodating “central pocket”. d) Design of the indole‐focused encoded peptidomimetic library.

We elected the Ugi four‐component reaction for the initial step in the design of an encoded library because it combines selectable linker moieties to the DNA, diversity elements, and handles for library expansion into a peptoid backbone (Figure 1 d). DNA‐encoded library synthesis was initiated by a Ugi reaction (U‐4CR, Figure 2 a) on the chemically stable, solid‐phase‐coupled hexathymidine adapter “hexT”, with a broad range of reaction conditions tolerated (Figures 2 b and S1).^[17]^ This strategy allows for the synthesis of target molecules from bulk hexT‐coupled starting materials in parallel, and it is more efficient than coupling individual multicomponent reaction products to DNA codes; all hexT products were isolated, thus providing fidelity. Carboxylic acid **hexT 1** and indole‐carboxaldehydes **hexT 2** and **hexT 3** were reacted either with an alkyne‐substituted amine (**hexT 1**, **hexT 2**, and **hexT 3**) or an alkyne‐substituted carboxylic acid (**hexT 2**) that served as handles for the library expansion step. Three tryptophan‐mimicking indole carbaldehydes and one tyrosine‐mimicking *p*‐hydroxybenzaldehyde placed the anchor motif distal from the DNA. A set of 18 isocyanides were used as diversity elements R^1^ in the Ugi reaction (Figure 2 b, Tables S1 and S2). Reaction optimization efforts identified temperatures of 80 °C and high concentrations of isocyanides as requisite for peptoid synthesis. We synthesized in total 78 hexT peptoids, which were then ligated in one pot to peptoid backbone‐ and isocyanide‐encoding DNA barcodes (Figures 2 c and S4).^[17]^ Pooling, splitting, a second barcode ligation step, and copper(I)‐promoted alkyne‐azide cycloaddition with 104 azides,^[18]^ —produced in situ from the corresponding halides beforehand— finalized the 8112‐membered “tiDEL” (thymidine‐initiated DEL; Figure S2 and Table S3). The library was selected against streptavidin for library validation (Figures 3 b and S7), against the p53‐binding domain of MDM2, and against the YAP‐interacting domain of human TEAD4 (hTEAD4). The sequencing data were progressed by the in‐house‐programmed algorithm ECEC (encoded compound enrichment calculator) based on R (Figures 3 a, S5, and S6). Two‐dimensional plots visualized enrichment factors of selection experiments versus bead‐only control selections to facilitate compound identification. For the MDM2 target, the most highly enriched peptoids contained 6‐chloroindole derivatives irrespective of their positioning on the backbone (Figure 3 c). Interestingly, a single building block from the 104 diverse azides was enriched, the 2,4‐dimethylphenylacetamide **A48**. Compound **8** was selected based on enrichment factor calculations. It showed a plausible in silico binding mode, and MST experiments confirmed binding to MDM2, thus validating the library design concept (Figures 4 a,b and S9).^[15]^ Selection experiments for hTEAD4 identified peptoid **hexT 21‐A56** as the most enriched compound (Figures 3 d and S8). In this peptoid, a 6‐chloroindole was flanked by a C‐terminal hydrocarbon and a triazole‐linked imidazopyridine. We synthesized a small series of compounds inspired by **hexT 21‐A56**/**9** and investigated their binding to depalmitoylated hTEAD4 by nanodifferential scanning fluorimetry (Figures 4 c,d and S10). Stabilization of hTEAD4 was observed for peptoids **9**—**11**, which differed in their C‐terminal alkyl amides, with a *tert*‐butyl amide leading to the highest Δ*T*_m_ value. Exchanging the succinate linker by acetamide **14** reduced the Δ*T*_m_ value, thereby suggesting that the linker was involved in protein binding.^[19]^ The imidazopyridine substituent could be exchanged by 5‐phenyloxazole‐2‐yl (to give **12**), as suggested by the enrichment plot (Figure S8). We next studied the biological consequences of the compound–hTEAD4 interaction. Compound **9** inhibited the palmitic acid–hTEAD4 interaction with an IC_50_ value of 0.41 μm, while compound **10** showed a much weaker inhibition, which suggests a different binding mode (Figure 4 e). Both compounds were then evaluated for inhibition of the YAP–hTEAD4 interaction (YAP^50–100^, Figure 4 f). They inhibited the PPI with IC_50_ values of 6.75 μm (**9**) and 5.65 μm (**10**).

**Figure 2:**
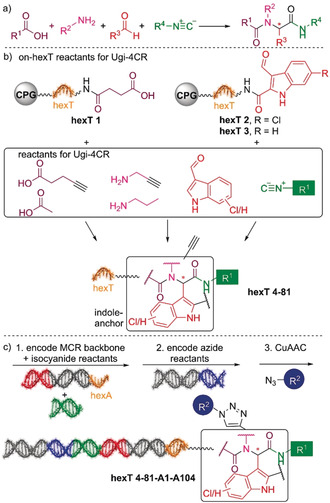
Library design and synthesis. a) Ugi four‐component reaction. b) Synthesis of the library: Ugi reaction followed by a click reaction. c) Barcoding strategy.

**Figure 3:**
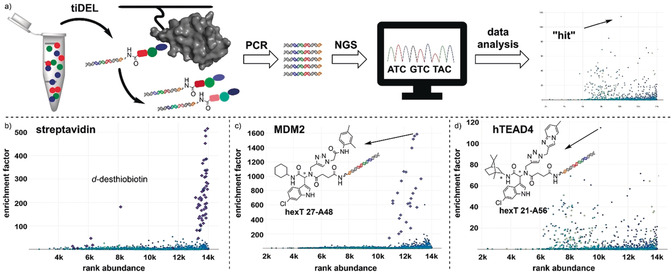
Identification of compounds by selection. a) Compound identification. b) Encoded library validation by streptavidin selection. c) Library selection for MDM2 identifies a 1,3‐dimethylanilide building block coupled to the peptoid. d) Selection of the library for hTEAD4 uncovered a novel class of potential TEAD4 binders.

**Figure 4:**
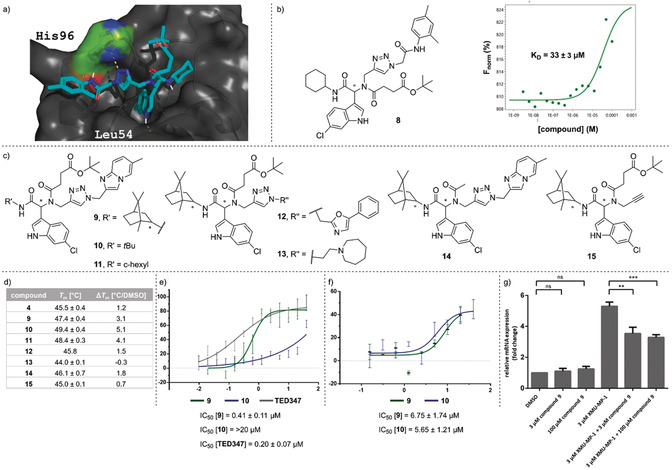
Validation of MDM2 and hTEAD4 binders. a) Docking of **8** into the MDM2–p53 interaction site. b) Effect of the off‐DNA‐synthesized **8** bound to MDM2. c) Chemical structures of biologically evaluated compounds **9**, **12**–**15**. d) Validation of compound binding to hTEAD4 by nanoDSF. e) Inhibition of palmitic acid binding to the hTEAD4 central pocket measured by fluorescence polarization. f) Inhibition of YAP binding to hTEAD4 measured by fluorescence polarization. g) Evaluation of the cellular activity of **9** by measurement of CTGF transcript expression levels.

Finally, we tested the cellular activity of compound **9** by measuring transcript levels of CTGF, a gene under control of the hippo pathway effectors TEAD–YAP (Figure 4 g). HEK293 cells were treated with compound **9** alone, and with a combination of compound **9** and the hippo signaling inhibitor XMU‐MP‐1. XMU‐MP‐1 blocks MST1/2 kinases which are upstream components in the Hippo pathway. This inhibition results in inactivation of downstream kinases LATS1/2, and subsequent translocation of YAP into the nucleus, where it forms a transcriptional complex with TEAD, thereby leading to gene expression. The addition of compound **9** to HEK293 cells did not alter the CTGF transcript levels, whereas it caused significant reduction in gene expression after inhibition of Hippo signaling by XMU‐MP‐1. This observation was in line with an on‐target mechanism and suggested a potential implication for treating tumors driven by abnormal Hippo pathway signaling.

Initiating encoded library synthesis with an Ugi multicomponent reaction step that turned simple starting materials into peptoid side chains provided flexibility in the library design around privileged “anchor” motifs such as tryptophan mimics. This library design uncovered chemical aspects of challenging target proteins from relatively few encoded compounds. Currently, we are elucidating the binding mode of compounds **9** and **10**, and we are synthesizing analogues to better understand the structure–activity relationships of these TEAD–YAP inhibitors.

## Conflict of interest

The authors declare no conflict of interest.

## Supporting information

As a service to our authors and readers, this journal provides supporting information supplied by the authors. Such materials are peer reviewed and may be re‐organized for online delivery, but are not copy‐edited or typeset. Technical support issues arising from supporting information (other than missing files) should be addressed to the authors.

Supplementary
