# Supplementary material for: TEAD–YAP Interaction Inhibitors and MDM2 Binders from DNA‐Encoded Indole‐Focused Ugi Peptidomimetics
Source: Angew Chem Int Ed Engl. 2020 Jul 15;59(46):20338–42. doi: 10.1002/anie.202006280 (PMC7689693; doi:10.1002/anie.202006280)
Supplement: Supplementary file 1 — Supplementary [file ANIE-59-20338-s001.pdf]

Angewandte Chemie
Eine Zeitschrift der Gesellschaft Deutscher Chemiker

Supporting Information

TEAD–YAP Interaction Inhibitors and MDM2 Binders from DNA-Encoded Indole-Focused Ugi Peptidomimetics

*Verena B. K. Kunig, Marco Potowski, Mohammad Akbarzadeh, Mateja Klika Škopić, Denise dos Santos Smith, Lukas Arendt, Ina Dormuth, Hélène Adihou, Blaž Andlovic, Hacer Karatas, Shabnam Shaabani, Tryfon Zarganes-Tzitzikas, Constantinos G. Neochoritis, Ran Zhang, Matthew Groves, Stéphanie M. Guéret, Christian Ottmann, Jörg Rahnenführer, Roland Fried, Alexander Dömling, and Andreas Brunschweiger**

anie_202006280_sm_miscellaneous_information.pdf

Table of Contents	Page

General methods and materials	S3
tiDEL synthesis and characterization	S5
Selection assay of tiDEL	S15
ECEC algorithm for enrichment factor calculation and visualization	S17
Synthesis and characterization of isocyanides **I1**-**I18**	S22
Synthesis and characterization of compounds **8**-**15**	S23
Synthesis and characterization of YAP peptide fragment **YAP$^{50-100}$**	S29
Evaluation of compound **8** as MDM2 binder	S31
Docking of compound **8** into the crystal structure of MDM2	S31
Expression and purification of MDM2	S31
MicroScale Thermophoresis (MST) Assay	S32
Biological evaluation of compounds **9**-**15** as hTEAD4 binder	S33
Expression and purification of hTEAD4	S33
Deacylation (depalmitoylation) of hTEAD4	S33
Nano Differential Scanning Fluorometry (nanoDSF)	S33
Fluorescence polarization (FP) experiments	S35
Measurement of CTGF expression by RT-qPCR	S36
Compound characterization data	S38
Supplementary references	S69

Supplementary Figures

Figure S1: Ugi four-component reactions on CPG-coupled hexT-conjugates **hexT 1**-**3**	S5
Figure S2: Copper-promoted azide-alkyne cycloaddition on CPG-coupled hexT-Ugi conjugates **hexT 4**-**81**	S6
Figure S3: Workflow of library synthesis and encoding strategy	S13
Figure S4: Agarose gel of tiDEL synthesis	S14
Figure S5: Section of the structure sheet of tiDEL for ECEC	S18
Figure S6: Section of the output of tiDEL selection analysis by ECEC	S19
Figure S7: 2D plots of selection experiment on streptavidin beads generated by ECEC	S20
Figure S8: Plot of enrichment factors obtained after tiDEL selection on hTEAD4	S21
Figure S9: Thermal protein stability curve obtained by MST	S32
Figure S10: Thermal protein stability curves obtained by nanoDSF	S34
Figure S11: Tracers used in the FP-based competitive binding assays	S35

Supplementary Tables

Table S1: Overview of the Ugi four-component reactions on CPG-coupled hexT-conjugate **hexT 1**	S9
Table S2: Overview of the Ugi four-component reactions on CPG-coupled hexT-conjugates **hexT 2**-**3**	S10
Table S3: Overview of *in situ* formed azides **A1**-**A104** used for the copper-promoted azide-alkyne cycloaddition	S11
Table S4: Sequences of DNA oligonucleotides ***I*** – ***IV/IV'***	S14

General methods and materials

Chemicals were purchased from ABCR, Acros Organics, Alfa Aesar, Fisher Scientific, Merck, Sigma Aldrich, TCI and VWR and were used as provided without further purifications. Dry solvents (DMF, EtOH, THF) were used as commercially available. 5’-Aminolinker-modified hexathymidine (hexT) oligonucleotide bound to controlled pore glass solid support (CPG, 1000 Å porosity) were synthesized by IBA (Göttingen, Germany). DNA barcodes for library synthesis were purchased by Integrated DNA Technologies (IDT). CPG-bound oligonucleotide-small molecule conjugates were filtered and washed through synthesis columns using a vacuum manifold (Vac-Man®) from Promega.

Semi-preparative ion pair RP-HPLC. Compound purification was performed on a Shimadzu Prominence HPLC system equipped with a C_{18} stationary phase (Gemini, 5 µm, C_{18}, 110 Å, 50 x 10 mm). A gradient from 100 mM aqueous triethylammonium acetate (pH = 8.0, eluent A) to MeOH (eluent B) was used at a flow rate of 5 mL/min. Fractions containing the desired product were pooled and concentrated.

> **Method:** Step gradient of 30% to 80% B within 13 min, then 80% to 100% B within 1 min followed by 100% B for 3 min using 100 mM aqueous triethylammonium acetate (pH = 8.0, eluent A) and MeOH (eluent B) at a flow rate of 5 mL/min.

MALDI-TOF. Mass analysis was performed on a MALDI TOF/TOF MS from Bruker Daltonics using 2’,4’,6’-trihydroxyacetophenone (THAP) matrix (Dichrom).

Analytical thin-layer chromatography (**TLC**) was performed on pre-coated TLC sheets ALUGRAM® Xtra SIL G/UV_{254} (silica gel 60 with fluorescent indicator UV_{254}) from Macherey-Nagel. Compounds were visualized by irradiation with UV light or potassium permanganate staining. **Column chromatography** was performed using silica gel NORMASIL 60 (particle size 0.040 - 0.063 mm) from VWR. Solvent mixtures are understood as volume/volume.

^{1}H-NMR and **^{13}C-NMR** were recorded on a Bruker AVANCE 400 (^{1}H NMR, 400 MHz; ^{13}C NMR, 101 MHz), Bruker AVANCE 500 (^{1}H NMR, 500 MHz; ^{13}C NMR, 126 MHz) or Bruker AVANCE 600 spectrometer (^{1}H NMR, 600 MHz; ^{13}C NMR, 151 MHz). Data are reported in the following order: chemical shift (δ) values are reported in ppm with the solvent resonance as internal standard ($CDCl_3$: δ = 7.26 ppm for ^{1}H, δ = 77.16 ppm for ^{13}C; CD_2Cl_2: δ = 5.32 ppm for ^{1}H, δ = 54.00 ppm for ^{13}C; MeOD: δ = 4.87 ppm for ^{1}H, δ = 49.00 ppm for ^{13}C) or relative to TMS (δ = 0 ppm); multiplicities are indicated s (singlet), d (doublet), t (triplet), q (quartet) m (multiplet); coupling constants (J) are given in Hertz (Hz).

Analytical RP-HPLC-MS (I) was performed using a Thermo Scientific Dionex® UltiMate 3000 equipped with a DAD-3000 detector (210, 254, 280 und 350 nm) coupled to a LCQ Fleet ion trap mass spectrometer. Heated electrospray ionization mass spectra were recorded in 110-2000 Da range using an EC Nucleodur C_{18} gravity column (150 mm x 3 mm, particle size 1.8 µm) from Macherey-Nagel.

> **Method:** A step gradient of 10% to 10% A for 0.5 min, then 10% to 95% A for 7 min was applied at a flow rate of 0.4 mL/min. Eluent A: ACN + 0.1% TFA, eluent B: H_2O + 0.1% TFA.

Analytical RP-HPLC-MS (II) was performed using an Agilent HPLC system (1100 series) equipped with an ESA Corona Detector coupled to a Thermo Scientific Finnigan LCQ Advantage Max ion trap mass spectrometer. Heated electrospray ionization mass spectra were recorded in 110-2000 Da range.

Compounds were eluted from a CC Nucleodur C_{18} gravity column (125 mm x 4 mm, particle size 3.0 µm, Macherey-Nagel).

Method: A step gradient from 1% to 10% A for 1 min, then 10% to 95% A for 9 min was applied at a flow rate of 0.4 mL/min. Eluent A: ACN + 0.1% TFA; eluent B: H_2O + 0.1% TFA.

High resolution mass spectra (**HRMS**) were recorded on a Thermo Electron LTQ Orbitrap fourier transformation mass spectrometer coupled to an HPLC-System (HPLC column: Hypersyl GOLD, 50 mm x 1 mm, particle size 1.9 µm, ionization method: electron spray ionization). Compound mass was recorded in the range of 150 to 2000 Da.

tiDEL synthesis and characterization

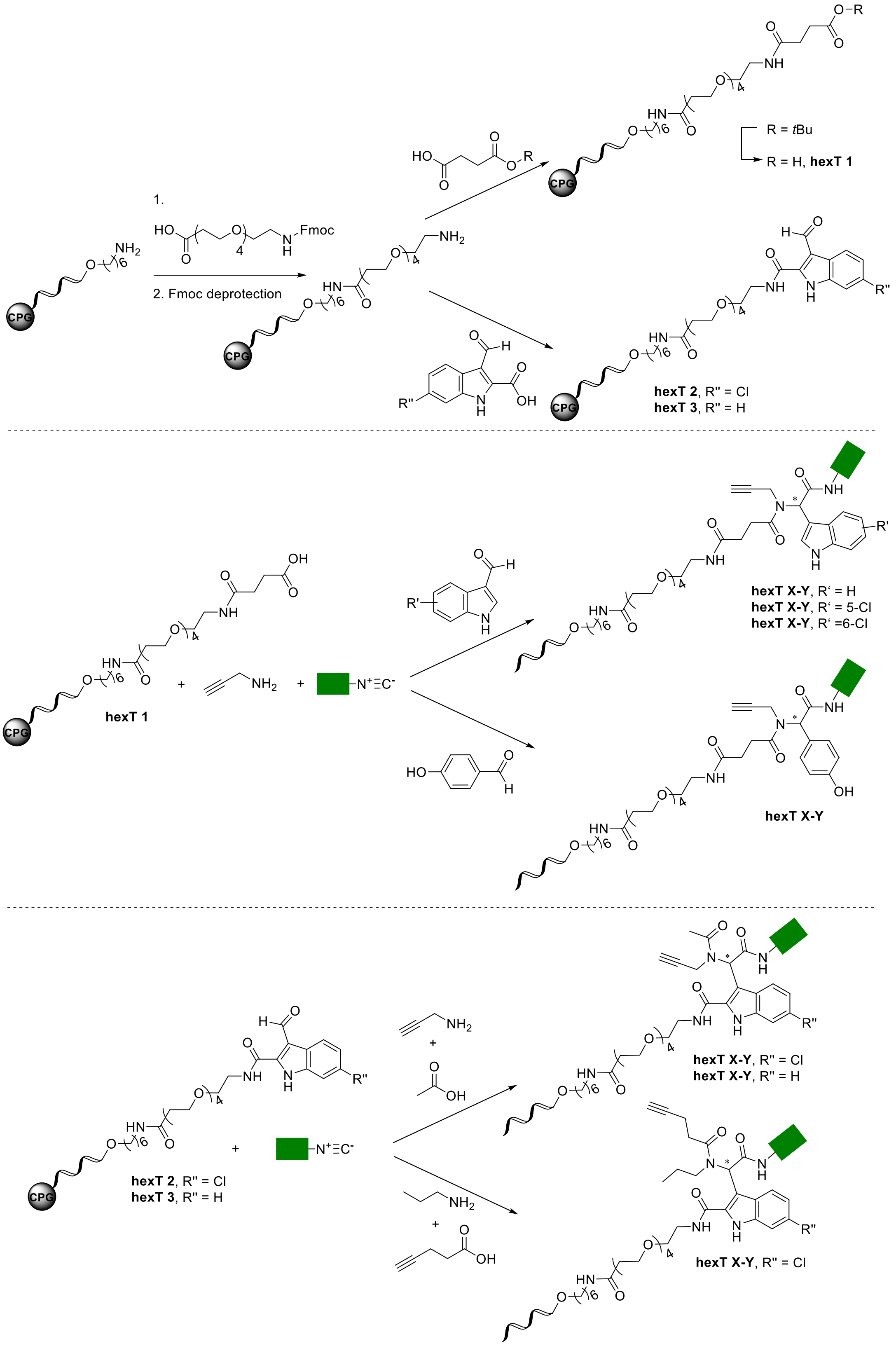

Figure S1. Ugi four-component reactions on CPG-coupled hexT-conjugates **hexT 1-3**.

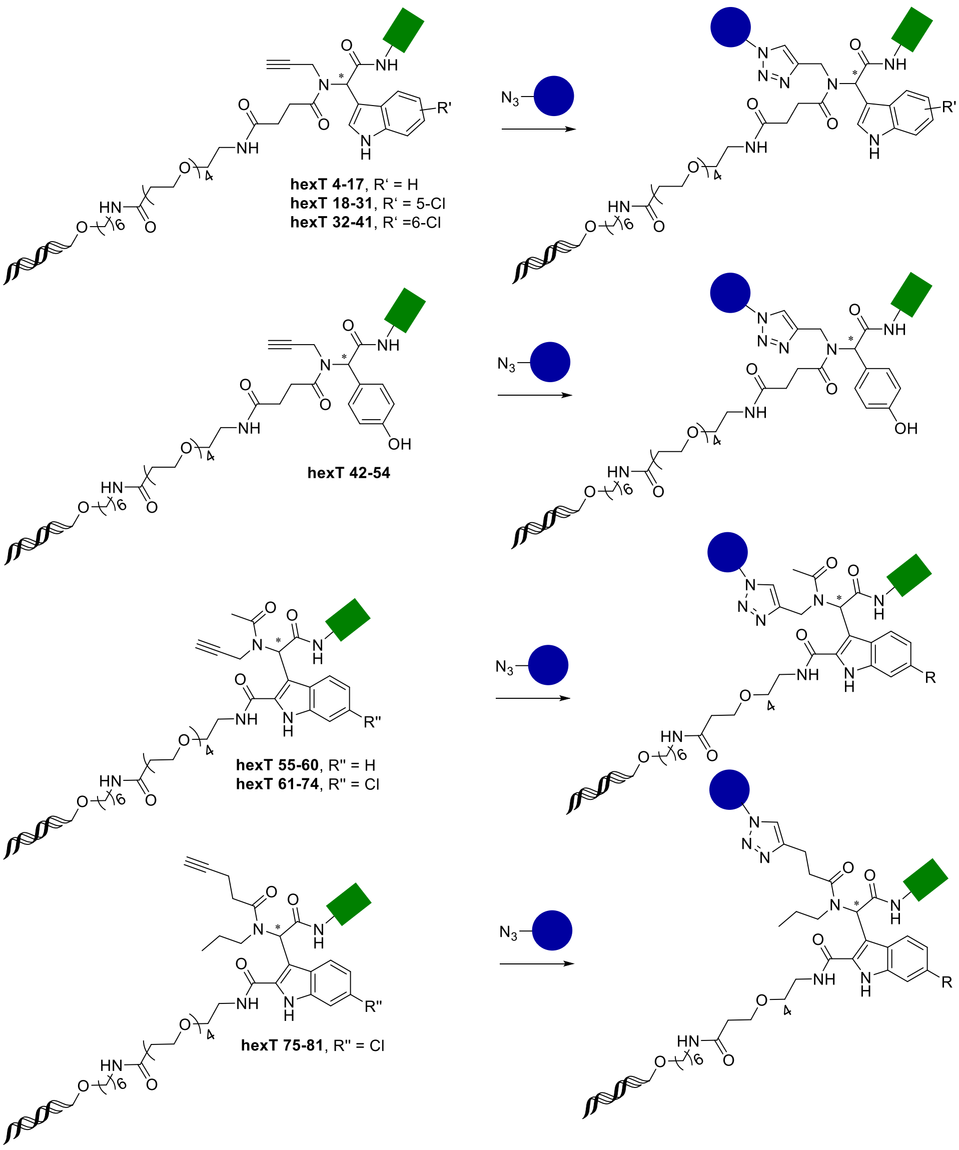

Figure S2: Copper-promoted azide-alkyne cycloaddition on CPG-coupled hexT-Ugi conjugates **hexT 4-81**.

Representative procedure for amide coupling (RP-01a)

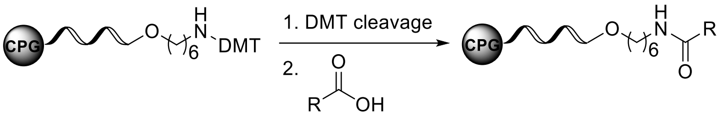

Step 1: The DMT-protective group of the CPG-bound hexathymidine oligonucleotide (250 nmol, 9-10 mg of solid phase material) was removed by addition of 200 µL 3% trichloroacetic acid in CH_2Cl_2 for 30 s – 60 s. An orange coloring of the solution indicated successful removal of the protecting group. The deprotection was repeated several times until no coloring of the solution was observed anymore. The CPG containing deprotected DNA was washed three times with each 200 µL of 1% TEA in ACN, DMF, MeOH, ACN and CH_2Cl_2 and then dried *in vacuo*.

Step 2: The CPG-bound hexathymidine oligonucleotide, the carboxylic acid and HATU were dried *in vacuo* for 15 min. Stock solutions of all reactants in dry DMF were prepared before the reaction was started. To the solution of the acid (25 µmol, 100 equiv.) in 75 µL dry DMF were added HATU (25 µmol, 100 equiv.) dissolved in 75 µL dry DMF and DIPEA (62.5 µmol, 250 equiv.). The mixture was shaken for 5 min and added to the solid support-bound DNA suspended in 75 µL dry DMF (250 nmol, 1 equiv.). The amide coupling reaction was shaken at ambient temperature for 2 h. Then, the CPG solid phase was filtered over a filter column, washed with each 3x 200 µL of DMF, MeOH, ACN and CH_2Cl_2 and dried *in vacuo*. The amide coupling was repeated once. The completeness of the amide coupling was controlled by a test cleavage of a small portion (0.7 – 0.9 mg, ~20 nmol) of the oligonucleotide conjugate with 500 µL AMA solution (AMA = aqueous ammonia (30%)/aqueous methylamine (40%), 1:1, vol/vol) for 30 min at ambient temperature. To this solution 20 µL of 1 M Tris-buffer (pH = 7.5) were added, the mixture was dried in a SpeedVac and dissolved in 200 µL of distilled water. The crude was analyzed by RP-HPLC and MALDI-MS. In case of uncompleted coupling (<90%), the reaction was repeated a third time.

Unreacted amines were capped with acetic acid anhydride (3x 200 µL, 30 s, 1:1 mixture of THF/methylimidazole, 9:1, vol/vol, and THF/pyridine/acetic acid anhydride, 8:1:1, vol/vol). The CPG was washed afterwards again with each 3x 200 µL of DMF, MeOH, ACN and CH_2Cl_2 and dried *in vacuo*.

Representative procedure for amide coupling (RP-01b)

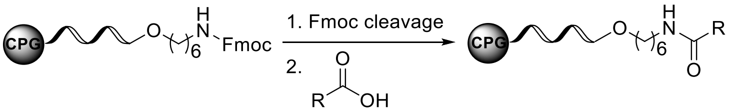

Step 1: The Fmoc-protecting group of a CPG-bound oligonucleotide conjugate (250 nmol, 9 – 10 mg) was removed by addition of 200 µL 20 % piperidine in dry DMF and shaking for 5 min. Afterwards, the CPG solid phase was washed with each 3x 200 µL of DMF, MeOH, ACN and CH_2Cl_2 and then dried *in vacuo*.

Step 2: The CPG-bound oligonucleotide, the carboxylic acid and HATU were dried *in vacuo* for 15 min. Stock solutions of all reactants in dry DMF were prepared before the reaction was started. To the solution of the carboxylic acid (25 µmol, 100 equiv.) in 75 µL dry DMF were added HATU (25 µmol, 100 equiv.) dissolved in 75 µL dry DMF and DIPEA (62.5 µmol, 250 equiv.). The mixture was shaken for 5 min and added to the solid support-bound hexT DNA (suspended in 75 µL dry DMF, 250 nmol, 1 equiv.). The amide coupling reaction was shaken at ambient temperature for 2 hours. Then, the solid phase was filtered over a filter column and washed with each 3x 200 µL of DMF, MeOH, ACN and CH_2Cl_2 and dried *in vacuo*. The amide coupling was repeated two times.

The completeness of the coupling reaction was controlled by a test cleavage of a small portion (0.7 – 0.9 mg, ~20 nmol) of the CPG-bound oligonucleotide conjugate with 500 µL AMA (AMA = aqueous ammonia (30%)/ aqueous methylamine (40%), 1:1, vol/vol) for 30 min at ambient temperature. To this solution 20 µL of 1 M Tris-buffer (pH = 7.5) were added, the mixture was dried in a SpeedVac,

re-dissolved in 200 µL of distilled water. The crude was analyzed by RP-HPLC and MALDI-MS. In case of uncompleted coupling the reaction was repeated a third time.

Unreacted amines were capped with acetic acid anhydride (3x 200 µL, 30 s, 1:1 mixture of THF/methylimidazole, 9:1, vol/vol, and THF/pyridine/acetic acid anhydride 8:1:1, vol/vol). The CPG-bound oligonucleotide conjugate was washed afterwards again with each 3x 200 µL of DMF, MeOH, ACN and CH_2Cl_2 and dried *in vacuo*.

Step 3: In the case of the presence of a *t*Bu protecting group, the *tert*-butyl group was removed with 500 µL 10% trifluoroacetic acid in CH_2Cl_2 for 4 h. Then, the CPG-solid phase with the deprotected conjugate was filtered over a filter column and washed with each 3x 200 µL of DMF, MeOH, ACN and CH_2Cl_2 and dried *in vacuo*.

Representative procedure for Ugi four-component reaction on CPG-bound hexT-conjugates (RP-02)

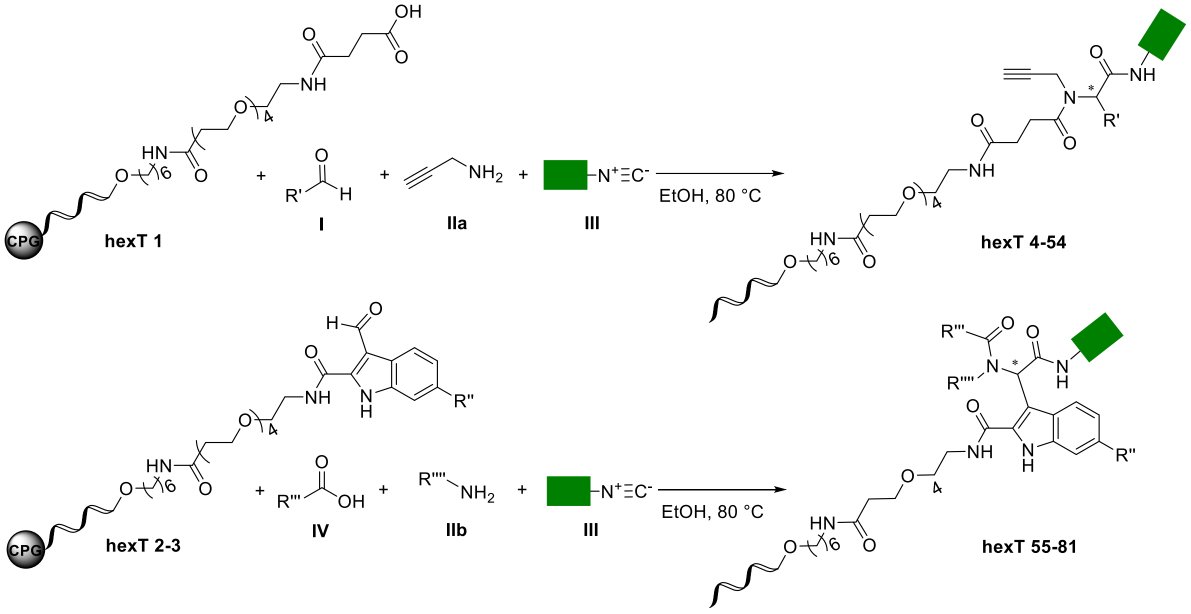

Aldehyde **I** (20 µmol, 1000 equiv.) or carboxylic acid **IV** (20 µmol, 1000 equiv.), amine **II** (20 µmol, 1000 equiv.) and isocyanide **III** (20 µmol, 1000 equiv.) were added to the CPG-bound DNA-acid conjugate **hexT 1** (20 nmol, 1 equiv.) or –aldehyde conjugate **hexT 2,3** (20 nmol, 1 equiv.). The reaction mixture was filled up to a final volume of 50 µL with EtOH and the reaction was shaken at 80 °C for 24 h. Then the CPG-solid phase with a conjugate **hexT 4-81** was filtered over a filter column, washed with each 3x 200 µL of DMF, MeOH, ACN and CH_2Cl_2 and dried *in vacuo*. The CPG-bound DNA conjugate **hexT 4-81** was cleaved from the solid phase with 500 µL AMA solution for 30 min at ambient temperature. Then, 20 µL of 1 M Tris buffer (pH = 7.5) were added to the crude, the mixture was dried in a SpeedVac and dissolved in 200 µL of distilled water. The crude was analyzed by MALDI-MS and the product was isolated by preparative RP-HPLC. Fractions containing the products were collected, coevaporated three times using water/ethanol (1:1, vol/vol), and re-dissolved in 40 µL ddH_2O.

Table S1 – Overview of the Ugi four-component reactions on CPG-coupled hexT-conjugate **hexT 1**.

	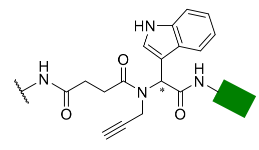	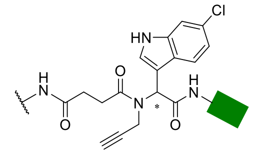	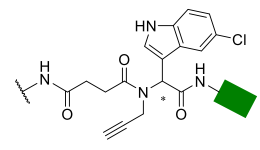	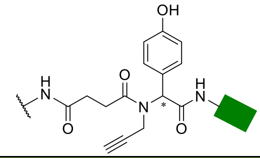
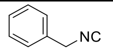 **I1**	**hexT 4**	**hexT 18**	**hexT 32**	**hexT 42**
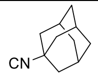 **I2**	**hexT 5**	**hexT 19**	**hexT 33**	**hexT 43**
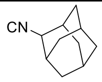 **I3**	**hexT 6**	**hexT 20**		**hexT 44**
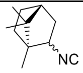 **I4**	**hexT 7**	**hexT 21**	**hexT 34**	
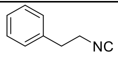 **I5**	**hexT 8**	**hexT 22**	**hexT 35**	**hexT 45**
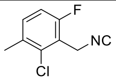 **I6**	**hexT 9**		**hexT 36**	
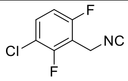 **I7**	**hexT 10**		**hexT 37**	
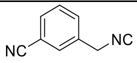 **I8**	**hexT 11**	**hexT 23**	**hexT 38**	**hexT 46**
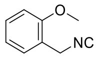 **I9**	**hexT 12**	**hexT 24**		**hexT 47**
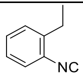 **I10**	**hexT 13**	**hexT 25**		**hexT 48**
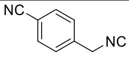 **I11**	**hexT 14**	**hexT 26**	**hexT 39**	**hexT 49**
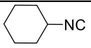 **I12**		**hexT 27**	**hexT 40**	**hexT 50**
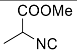 **I13**		**hexT 28**	**hexT 41**	
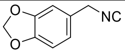 **I14**	**hexT 15**	**hexT 29**		**hexT 51**
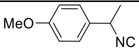 **I15**	**hexT 16**	**hexT 30**		**hexT 52**
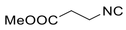 **I16**				
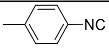 **I17**	**hexT 17**	**hexT 31**		**hexT 53**
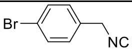 **I18**				**hexT 54**

Table S2 – Overview of the Ugi four-component reactions on CPG-coupled hexT-conjugates **hexT 2-3**.

	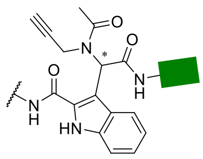	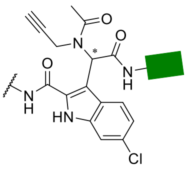	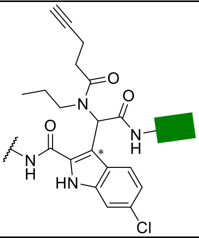
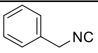 **I1**		**hexT 61**	**hexT 75**
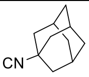 **I2**	**hexT 55**	**hexT 62**	**hexT 76**
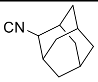 **I3**	**hexT 56**	**hexT 63**	**hexT 77**
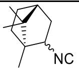 **I4**		**hexT 64**	
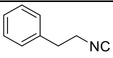 **I5**		**hexT 65**	**hexT 78**
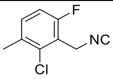 **I6**		**hexT 66**	
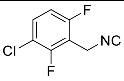 **I7**			
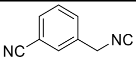 **I8**		**hexT 67**	
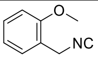 **I9**		**hexT 68**	
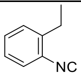 **I10**	**hexT 57**	**hexT 69**	
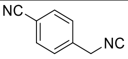 **I11**	**hexT 58**	**hexT 70**	
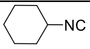 **I12**	**hexT 59**	**hexT 71**	**hexT 79**
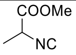 **I13**			
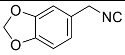 **I14**		**hexT 72**	
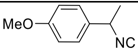 **I15**	**hexT 60**	**hexT 73**	**hexT 80**
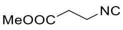 **I16**			**hexT 81**
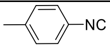 **I17**			

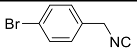 I18		hexT 74	

successful reaction	failed reaction	reaction not performed

Representative procedure for copper-promoted azide-alkyne cycloaddition on hexT-peptidomimetic conjugates (RP-03)

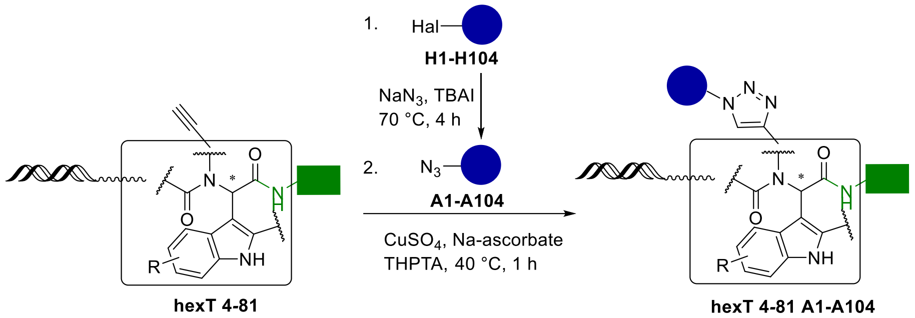

All azides were prepared freshly prior azide-alkyne cycloaddition (Table S3). To an alkyl or benzyl halide **H1-104** (10 µmol) dissolved in 800 µL DMF were added 100 µL of an aqueous solution of sodium azide (123 µmol, 8 mg/100 µL) and tetrabutylammonium iodide dissolved in DMF (100 µL, 20 µmol, 7.5 mg/100 µL). The reaction mixture was shaken for 4 hours at 70 °C. Afterwards, an aliquot of 2 µL of each azide solution **A1-104** (20 nmol, 250 equiv.), 1.5 µL of an aqueous tris(3-hydroxypropyltriazolylmethyl)amine (THPTA)/$CuSO_4$ solution (10 nmol, 125 equiv.), and 1.6 µL of an aqueous sodium ascorbate solution (16 nmol, 200 equiv.) were added to the solution of the pooled DNA-encoded hexT-peptidomimetic conjugates **hexT 4-81** (80 pmol, 1 equiv.) in 45 µL ddH_2O The reaction mixture was shaken at 40 °C for 1 h. Then, the reaction mixtures of the azide-alkyne cycloaddition were pooled and three times precipitated with 75% ethanol and 0.1 mM EDTA at -80 °C overnight.

Table S3 – Overview of *in situ* formed azides **A1-A104** used for the copper-promoted azide-alkyne cycloaddition on **hexT 4-81**.

**A1**	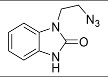	**A2**	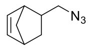	**A3**	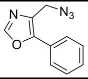	**A4**	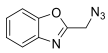
**A5**	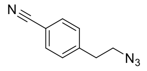	**A6**	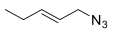	**A7**	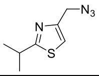	**A8**	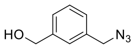
**A9**	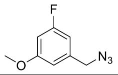	**A10**	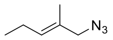	**A11**	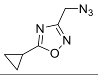	**A12**	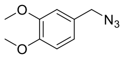
**A13**	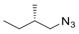	**A14**	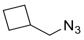	**A15**	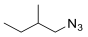	**A16**	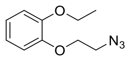
**A17**	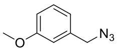	**A18**	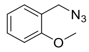	**A19**	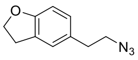	**A20**	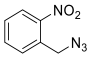
**A21**	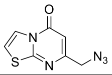	**A22**	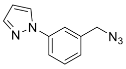	**A23**	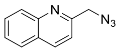	**A24**	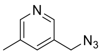

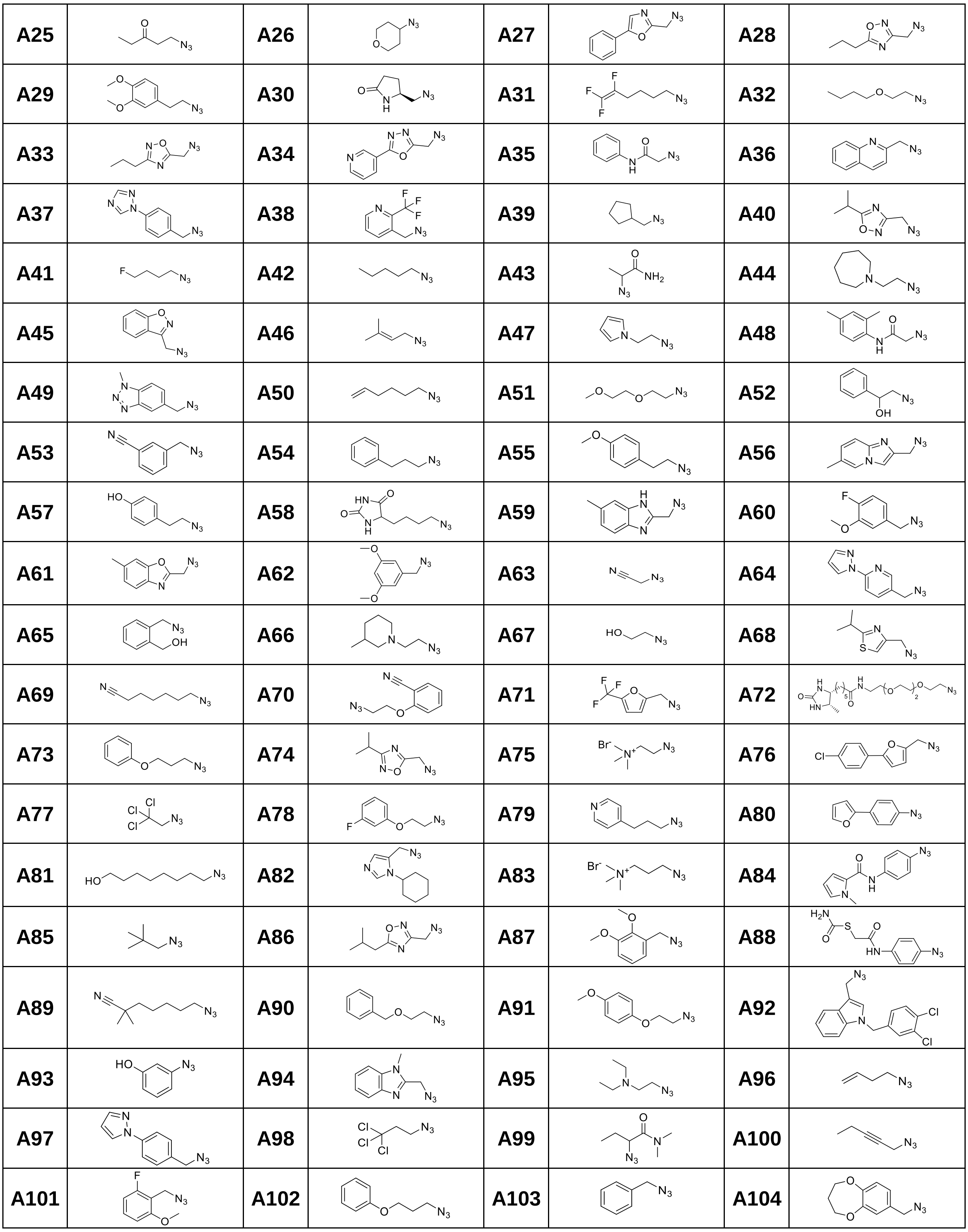
A25
A26
A27
A28
A29
A30
A31
A32
A33
A34
A35
A36
A37
A38
A39
A40
A41
A42
A43
A44
A45
A46
A47
A48
A49
A50
A51
A52
A53
A54
A55
A56
A57
A58
A59
A60
A61
A62
A63
A64
A65
A66
A67
A68
A69
A70
A71
A72
A73
A74
A75
A76
A77
A78
A79
A80
A81
A82
A83
A84
A85
A86
A87
A88
A89
A90
A91
A92
A93
A94
A95
A96
A97
A98
A99
A100
A101
A102
A103
A104

tiDEL synthesis

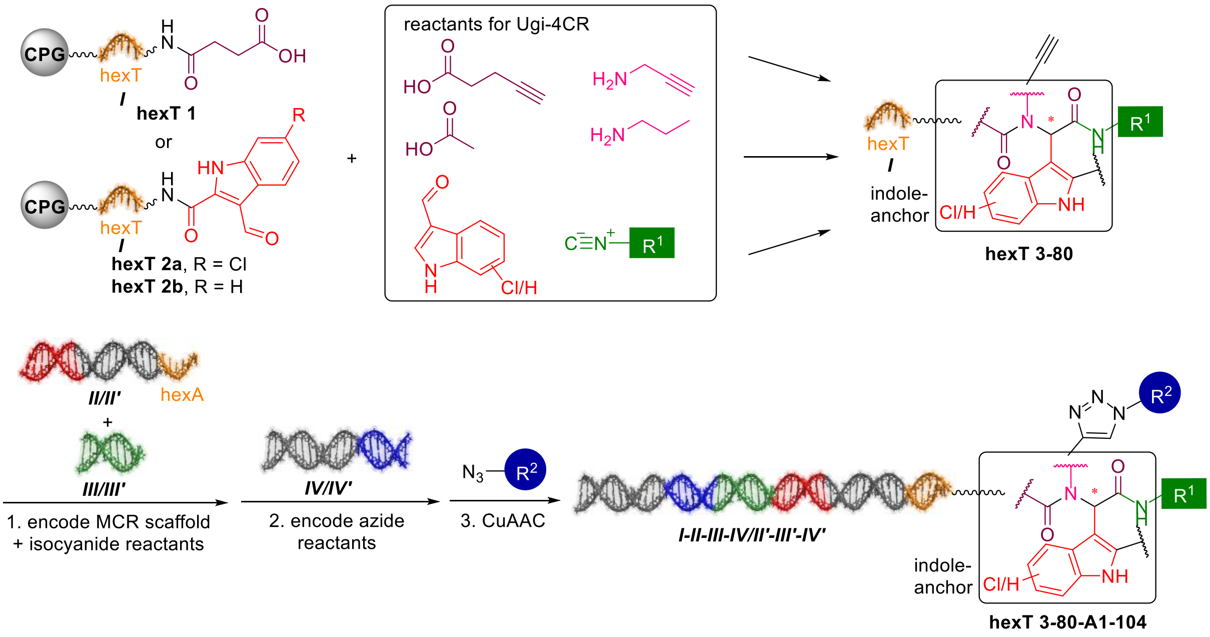

Figure S3: Workflow of library synthesis and encoding strategy.

In total, 78 HPLC-purified hexT-Ugi conjugates **hexT 4-81** depicted as oligonucleotide ***I*** were placed in one 96-well plate and annealed with enzymatically phosphorylated oligonucleotides ***II*/*II*´** and ***III*/*III*´** at 85 °C for 10 min and slowly cooled down to 4 °C. The total reaction volume was set to 40 µL. This contained 100 pmol of each DNA oligonucleotide. Ligations to encode the peptoid backbone and the isocyanide building block were carried out at 25 °C for overnight and stopped by heat inactivation at 75 °C for 15 min. All duplex DNA strands ***I-II-III/II´-III´*** corresponding to encoded hexT-Ugi conjugates were pooled and precipitated. The pellet was dissolved in IDTE buffer and the concentration was determined by Nanodrop measurement. The pooled and encoded hexT-conjugates were distributed in two 96-well plates (104 wells, 80 pmol/well). Then, the free 5'-end of the duplex DNA ***I-II-III/II´-III´*** and 104 oligonucleotides ***IV*** that contained the code for the azide building blocks **A1-104** were phosphorylated with PNK. For ligation, the duplex DNA sequences ***I-II-III/II´-III´*** and ***IV/IV'*** were annealed at 85 °C for 10 min and slowly cooled down to 4 °C. After the ligation at 25 °C for overnight, the DNA ligase was heat-inactivated at 75 °C for 15 min and the duplex DNA oligonucleotides ***I-II-III-IV/II´-III´-IV´*** were precipitated separately. The pellets were dissolved in ddH_2O and the duplex DNA ***I-II-III-IV/II´-III´-IV´*** were transferred into 104 glass vials. 104 halides were *in situ* transformed into azides with sodium azide. Azide-alkyne cycloaddition was performed at 40 °C for 60 min. Then, the reaction mixtures of the azide-alkyne cycloaddition were pooled and purified three times by ethanol precipitation using 0.1 mM EDTA.

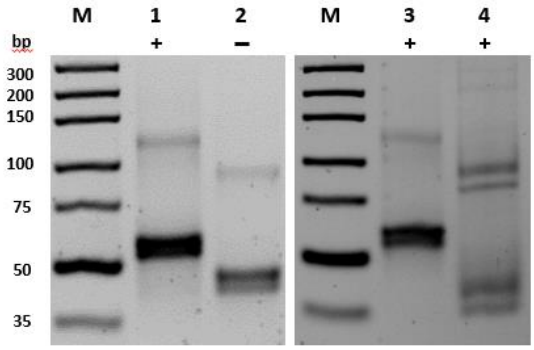

Figure S4. Agarose gel of tiDEL synthesis. Gel electrophoresis (5.5% agarosel gel) of the ligation of hexT-conjugates to DNA duplexes ***II/II´*** and ***III/III´*** in one pot to encode the peptoid backbone and the isocyanide building block yielding the duplex ***I-II-III/II´-III´*** and ligation of DNA duplex sequences ***I-II-III/II´-III´*** to the DNA duplex ***IV/IV´*** to encode azide building blocks; lane 1: ligation of duplexes ***II/II´*** and ***III/III'*** to hexT-Ugi conjugates **hexT 4-81**; lane 2: negative control w/o T4 ligase; lane 3: ethanol precipitation of ligation of duplexes ***II/II´*** and ***III/III'*** to hexT-Ugi conjugates **hexT 4-81**; lane 4: gel electrophoresis of the ligation of DNA duplex sequences ***I-II-III/II´-III´*** to the DNA duplexes ***IV/IV´*** to encode azide building blocks **A1-104**.

Table S4: Sequences of DNA oligonucleotides ***I*** – ***IV/IV'***.

DNA	sequence (5'-3')
I	TTT TTT
II	GAA TTC AGG TCG GTG TGA ACG GAT TTG XXX XXX XX
II'	ATA CXX XXX XXX CAA ATC CGT TCA CAC CGA CCT GAA TTC AAA AAA
III	GTA TXX XXX XXX
III'	TAG GXX XXX XXX
IV	CCT AXX XXX XXX TGA CCT CAA CTA CAT GGT CTA CA
IV'	TGT AGA CCA TGT AGT TGA GGT CAX XXX XXX X

5'-phosphorylation of DNA

For 5'-phosphorylation of 280 pmol DNA in a total reaction volume of 20 µL, 10 U of T4 polynucleotide kinase (T4 PNK, Thermo Fisher Scientific), 1x PNK Buffer A (500 mM Tris-HCl, 100 mM $MgCl_2$, 50 mM DTT, 1 mM spermidine, pH = 7.6, 25 °C, Thermo Fisher Scientific) and 1 mM ATP (Thermo Fisher Scientific) were used. Reaction mixtures were incubated at 37 °C for 20 min, then heat-inactivated at 75 °C for 15 min and slowly cooled down to 4 °C.

Ligation of DNA

Prior to enzymatic ligation of DNA, the oligonucleotides were annealed by incubation at 85 °C for 10 min and cooling down to 4 °C. For ligation (40 µL scale), 100 pmol or 80 pmol of each oligonucleotide, 600 U of T4 DNA Ligase (T4 DNA ligase rapid, Biozym) and 1x T4 DNA Ligase Buffer (500 mM Tris-HCl, 100 mM $MgCl_2$, 50 mM DTT, 10 mM ATP, pH = 7.6 at 25 °C) were mixed. Ligation reactions were performed at 25 °C for 16 h, then stopped by heat inactivation at 75 °C for 15 min and cooled down to 4 °C.

Analysis of DNA ligation

For analysis of DNA ligation reactions, agarose gel electrophoresis was performed. Using a 5.5% agarose gel, electrophoresis was carried out in TBE buffer (89 mM Tris-borate, 2 mM EDTA, pH = 8.3) at 100 V constant voltage for 15 min and then 150 V constant voltage for about 45 min. For staining of

the DNA, Midori Green Direct (NIPPON Genetics) and as a reference, GeneRuler Ultra Low Range DNA Ladder (Thermo Fisher Scientific) was used. Imaging of the gels was performed using the Bio-Rad Gel Doc™ XR system.

Purification of DNA by ethanol precipitation

DNA was precipitated by adding 1/10 volume of 3 M aqueous sodium acetate (pH = 5.2) and 3 volumes of ethanol and storing this solution for overnight at -80 °C. Afterwards, the samples were centrifuged at 4 °C for 30 min (13200 rpm; Centrifuge 5415 R, Eppendorf), the supernatant was taken off and the DNA pellets were dried. The DNA samples were dissolved in DNA storage buffer or in ddH_2O.

Selection assay of tiDEL

Selection assay

HisPur™ Ni-NTA Magnetic Beads (Thermo Scientific) or Pierce™ Streptavidin Magnetic Beads (Thermo Scientific) were transferred to an Eppendorf tube and placed on magnet. Beads were washed four times with 100 µL PBS buffer (+ 0.01% Tween®-20) and then split (4 µL for each selection experiment). Beads were washed two times with 100 µL immobilization buffer (protein buffer). Afterwards, 10 µL of His-tagged protein (4.8 µg) were incubated with the magnetic beads for 30 min at 4 °C with continuous gentle mixing. Then, beads were washed two times with 100 µL immobilization buffer, one time with 100 µL bead blocking buffer (immobilization buffer + 0.1 mg/mL BSA + 0.2 mg/mL herring sperm DNA) and again two times with 100 µL immobilization buffer. Afterwards, 1 µL of DEL (250 fmol) was incubated in 49 µL immobilization buffer with the protein-coated beads for 45 min at room temperature with continuous gentle mixing. Then, beads were washed eight times with 100 µL washing buffer (immobilization buffer + 0.1 mg/mL BSA). DNA conjugates were eluted by heat denaturation of the protein (80 °C, 5 min). Heat denaturation was performed two times in 20 µL ddH_2O. Selection assay was performed a second time as described above using 20 µL of the eluted DNA conjugates and 30 µL of immobilization buffer. Afterwards, the coding DNAs of the oligonucleotide conjugates were amplified by barcoding PCR to elongate compound barcodes with selection experiment-encoding sequences and adapters for Illumina next-generation sequencing.

hTEAD4 protein buffer: 25 mM MES, pH = 6.5, 0.05% CHAPS

MDM2 protein buffer: 25 mM HEPES, 150 mM NaCl, 1 mM TCEP, pH = 8.0, 5% glycerol, 0.01% Tween-20

Streptavidin control experiment: PBS, pH = 7.4, 0.01% Tween-20

Barcode PCR

Following selection experiments, the coding DNA of the oligonucleotide-compound conjugates was amplified by PCR. In a first PCR run, the Illumina Nextera Transposase Adapters were introduced using 10 µL eluted DNA-conjugates directly from the selection assay without further purification, 5 U of Taq DNA polymerase (Thermo Fisher Scientific), 1x Taq Buffer (100 mM Tris-HCl (pH = 8.8 at 25 °C), 500 mM KCl, 0.8% (v/v) Nonidet P40, Thermo Fisher Scientific), 3 mM $MgCl_2$, 0.625 mM of each dNTP (dATP, dGTP, dTTP, and dCTP, corresponding to 2.5 mM of the mixture of dNTPs) and 1 µM of each primer in a total reaction volume of 40 µL. The PCR program started with pre-denaturation at 95 °C for 3 min, followed by denaturation for 30 s at 95 °C, annealing for 30 s at 55 °C, and elongation for 30 s at 72 °C. After 25 cycles in total, the time for elongation was prolonged to 5 min. In a second PCR, dual indices and Illumina sequencing adapters using the Nextera XT Index Kit were attached to the amplified DNA oligonucleotides. For this, 10 µL DNA template (PCR 1, unpurified), 5 U of Taq DNA polymerase (Thermo Fisher Scientific), 1x Taq Buffer (100 mM Tris-HCl (pH = 8.8 at 25 °C), 500 mM KCl, 0.8% (v/v) Nonidet P40, Thermo Fisher Scientific), 3 mM $MgCl_2$, 0.625 mM of each dNTP (dATP, dGTP, dTTP, and dCTP, corresponding to 2.5 mM of the mixture of dNTPs), 5 µL Nextera XT Index 1 primer and 5 µL Nextera XT Index 2 primer in a total reaction volume of 40 µL were used. The PCR program started with pre-denaturation at 95 °C for 3 min, followed by denaturation for 30 s at 95 °C, annealing for 30 s at 55 °C, and elongation for 30 s at 72 °C. After 15 cycles in total, the time for elongation was prolonged to 5 min. The PCR products were analyzed by agarose gel electrophoresis (4% agarose gel) and then purified using the QIAquick PCR Purification Kit (Qiagen). The DNA concentration was determined by UV spectroscopy and barcoded oligonucleotide sequences from different selection experiments were pooled. High-throughput sequencing with the MiSeq protocol was performed by CeGat GmbH (Tübingen, Germany).

ECEC algorithm for enrichment factor calculation and visualization

Encoded compound enrichment calculator ECEC

The software ECEC (encoded compound enrichment calculator) was developed in a collaboration between the Faculty of Chemistry and Chemical Biology and the Department of Statistics at TU Dortmund University. With this tool, DNA-encoded library screening results can be analyzed that are based on combinations of building blocks of DNA sequences. The functionality includes the identification of compound-encoding DNA barcodes, DNA barcode positions in a DNA-encoded library, types of sequencing errors, the calculation of combinations of DNA barcode blocks making up an encoded molecule, called herein connectivities, the calculation of enrichment factors comparing relative frequencies of connectivities between selections on target proteins and control experiments, and the visualization of the results of the enrichment analysis. The tool is written in the freely available statistical software R[1], using also additional R packages. To establish a reproducible procedure for different datasets of screening results, the algorithm is constructed in a flexible way, allowing different values for variables and different quantities to be calculated.

Setup of the algorithm

The basis of the algorithm is to split the DNA sequencing results for a selection experiment into code blocks, where DNA barcode sequences are represented as strings built up by the letters A, T, C, G and N. The letters A, T, C and, G indicate corresponding nucleobases of the sequence, and the letter N indicates a not-readable letter of the sequence. The input for the algorithm is a so-called Structure Sheet (in the current version an Excel Sheet as shown in Figure S5) that provides DNA barcode sequences; information about combinations of DNA barcode blocks, the chemical building blocks encoded by each barcode sequence, and the intermediate partial sequences between the building block-coding DNA sequences, i.e. the overhang sequences used to ligate DNA barcodes. The intermediate partial sequences are fixed enabling the algorithm to identify the start and end positions of building block-coding sequences within a sequence. In this DNA-encoded library, the following three building blocks represent a sequence:

code 1: *Ugi backbone*

code 2: *isocyanide building block*

code 3: *azide building block*

A

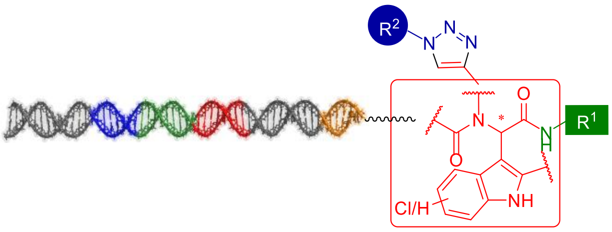

B

Region3Code_length	Region3Code_ID	Region3Code_name	Region3Code_sequence	Region3Code_smiles
8	A001	1-(2-azidoethyl)-1,	GCTTCAGA	[N-]=[N+]=NCCn1c(=O)[nH]c2ccccc
	A002	4-(2-aminoethyl)b	GCTAGGTA	N#Cc1ccc(CCN)cc1
	A003	1-(azidomethyl)-3-	GCTAACCT	COc1cc(F)cc(CN=[N+]=[N-])c1
	A004	(S)-1-azido-2-me	GCCAAGAT	CC[C@H](C)CN=[N+]=[N-]
	A005	1-(azidomethyl)-3-	GCATGATT	COc1cccc(CN=[N+]=[N-])c1
	A006	7-(azidomethyl)-5	GCATCTAT	[N-]=[N+]=NCc1cc(=O)n2ccsc2n1
	A007	1-azidopentan-3-	GCATCCTA	CCC(=O)CCN=[N+]=[N-]
	A008	4-(2-azidoethyl)-1	GCAGTTAC	COc1ccc(CCN=[N+]=[N-])cc1OC
	A009	5-(azidomethyl)-3	GATTGATC	CCCc1noc(CN=[N+]=[N-])n1
	A010	1-(4-(azidomethyl)	GATGTTAG	[N-]=[N+]=NCc1ccc(-n2cncn2)cc1
	A012	1-azido-4-fluorob	GATGTCTA	[N-]=[N+]=NCCCCF
	A013	3-(azidomethyl)be	GATGCTGA	[N-]=[N+]=NCc1noc2ccccc12
	A014	5-(azidomethyl)-1-	GATCTGAT	Cn1nnc2cc(CN=[N+]=[N-])ccc21
	A015	3-(azidomethyl)be	GAGGAAGT	N#Cc1cccc(CN=[N+]=[N-])c1
	A016	4-(2-azidoethyl)pl	GAGCTACA	[N-]=[N+]=NCCc1ccc(O)cc1
	A017	2-(azidomethyl)-6	GAGCATAG	Cc1ccc2nc(CN=[N+]=[N-])oc2c1
	A018	(2-(azidomethyl)pl	GACTTGAC	[N-]=[N+]=NCc1ccccc1CO
	A019	7-azidoheptaneni	GACTGTCT	N#CCCCCCCN=[N+]=[N-]
	A020	(3-azidopropoxy)b	GACATCTC	[N-]=[N+]=NCCCOc1ccccc1
	A021	2-azido-1,1,1-trich	GAATGGTA	[N-]=[N+]=NCC(Cl)(Cl)Cl
	A022	2-azido-1,1,1-trich	GAAGACAG	[N-]=[N+]=NCC(Cl)(Cl)Cl
	A023	1-azido-2,2-dimet	GAACTTGT	CC(C)(C)CN=[N+]=[N-]
	A024	7-azido-2,2-dime	GAACGCTT	CC(C)(C#N)CCCCCN=[N+]=[N-]

Figure S5: Section of the structure sheet of tiDEL for ECEC. A. Schematic representation of the fully encoded library (code 1: *Ugi backbone*, code 2: *isocyanide building block R1*, code 3: *azide building block R2*). B. Part of a Structure Sheet for code 3 (azide building block R2) with variables for sequence length, ID for the sequence variant, name of the variant, string of characters (denoted as 'sequence') of the variant, and SMILE representing the structural formula.

Identification of sequencing errors

Running an algorithm for the analysis of the resulting string of characters for each sequence is complicated by sequencing errors within the code blocks. After identifying the position of the intermediate parts, the algorithm proceeds by checking each code block for possible errors and counting errors per code block. In the case of a series of nucleobases, these errors can be either mutations (one nucleobase swapped with another at the same position), or insertions (a nucleobase added into a code block) or deletions (a missing nucleobase), in contrast to matches representing nucleobases at their desired position.

After loading the Structure Sheet, the algorithm identifies the first intermediate partial sequence of a sequence and the screening results, which are stored in a fastq file. Then, out of all possible combinations of code blocks, the best fitting combination can be identified by calculating the generalized Levenshtein distance of the sequence to all possible outcomes, using the R function adist. However, runtime would be very high, due to time complexity of O(mn) for a sequence part of length m and n possible outcomes. Alternatively, the algorithm iteratively runs through a series of functions comparing the given sequence part with an increasing number of error combinations. This approach requires a threshold for the maximal number of errors allowed in a code block (for the current version of the

algorithm this threshold is 2, regardless of the length of the code block), since the number of error combinations also increases exponentially. In the case of too many errors in one sequence part, the function adist is used to approximate the number of errors.

The output of this analysis step in R is a data frame that contains the results for all sequences, shown in Figure S6. This structure enables the calculation of interesting quantities that are later used to generate two-dimensional plots and corresponding tables for descriptive analysis.

```
  Structure_Sheet ProteinCode_ID ProteinCode_name ProteinCode_code ProteinCode_expCondition ProteinCode_Errors
1            VDEL              2        alpha ear         CGTACTAG                   Normal           No Error
2            VDEL              2        alpha ear         CGTACTAG                   Normal           No Error
3            VDEL              2        alpha ear         CGTACTAG                   Normal           No Error
4            VDEL              2        alpha ear         CGTACTAG                   Normal           No Error
5            VDEL              2        alpha ear         CGTACTAG                   Normal           No Error
6            VDEL              2        alpha ear         CGTACTAG                   Normal           No Error
  ProteinCode_ErrorPosition1 ProteinCode_ErrorPosition2 ProteinCode_Flipindex Zwischenteil1_ID Zwischenteil1_name
1                No Position                No Position                     0              ZT1      Zwischenteil1
2                No Position                No Position                     0              ZT1      Zwischenteil1
3                No Position                No Position                     0              ZT1      Zwischenteil1
4                No Position                No Position                     0              ZT1      Zwischenteil1
5                No Position                No Position                     0              ZT1      Zwischenteil1
6                No Position                No Position                     0              ZT1      Zwischenteil1
     Zwischenteil1_code Zwischenteil1_smile Zwischenteil1_Errors Zwischenteil1_ErrorPosition1 Zwischenteil1_ErrorPosition2
1 AGGTCGGTGTGAACGGATTTG            No Smile             No Error                  No Position                  No Position
2 AGGTCGGTGTGAACGGATTTG            No Smile             No Error                  No Position                  No Position
3 AGGTCGGTGTGAACGGATTTG            No Smile             No Error                  No Position                  No Position
4 AGGTCGGTGTGAACGGATTTG            No Smile             No Error                  No Position                  No Position
5 AGGTCGGTGTGAACGGATTTG            No Smile             No Error                  No Position                  No Position
6 AGGTCGGTGTGAACGGATTTG            No Smile             No Error                  No Position                  No Position
  Zwischenteil1_Flipindex Region1Code_ID Region1Code_name Region1Code_code
1                       0          No ID          No Name          No Code
2                       0      Scaffold4        ScaffoldH         GACGGAAC
3                       0      Scaffold6        ScaffoldK         CTGATTAG
4                       0      Scaffold3        ScaffoldC         CAGAGCAG
5                       0      Scaffold6        ScaffoldK         CTGATTAG
6                       0      Scaffold1        ScaffoldA         CATTCTGG
                              Region1Code_smile Region1Code_Errors Region1Code_ErrorPosition1
1                                      No Smile    Too many errors                No Position
2   C#CCN(C(C)=O)C(C(N)=O)c1c(C(=O)O)[nH]c2cc(Cl)ccc12           No Error                No Position
3 C#CCCC(=O)N(CCC)C(C(N)=O)c1c(C(=O)O)[nH]c2cc(Cl)ccc12           No Error                No Position
4    C#CCN(C(=O)CCC(=O)O)C(C(N)=O)c1c[nH]c2ccc(Cl)cc12           No Error                No Position
5 C#CCCC(=O)N(CCC)C(C(N)=O)c1c(C(=O)O)[nH]c2cc(Cl)ccc12           No Error                No Position
6         C#CCN(C(=O)CCC(=O)O)C(C(N)=O)c1c[nH]c2ccccc12           No Error                No Position
```

Figure S6: Section of the output of tiDEL selection analysis by ECEC. Part of a data frame containing the results for all sequence in the corresponding rows; the result for each sequence starts with an identifier related to the used Structure Sheet and proceeds with sequentially summing up information about each code block related to the variables given in the Structure Sheet.

Calculation of enrichment factors

The next step in the analysis of the screening results is the calculation of enrichment factors for so-called connectivities. Connectivities are combinations of chemical building blocks making up the compound binding to a target protein. The observed numbers of connectivities in the dataset between a target protein and a control (streptavidin beads, empty beads, a selection condition, or the amplified library) and the factors between the relative frequencies (of observed numbers and reads) are calculated. The ratio between these numbers, normalized to the corresponding numbers of reads for the protein and the control experiment, are called enrichment factors. The enrichment factors indicate the compounds binding to a given target protein.

For example, for the connectivity $\mathrm{connectx, y, z} = \{\mathrm{Scaffoldx, BB1y, BB2z}\}$ for a target protein and a control the frequencies are calculated by

$$\mathrm{freqprotein(connectx, y, z)} = |\{\{Sequences\ with\ \mathrm{connectx, y, z}\} \cap \{Sequences\ with\ \mathrm{protein}\}|,$$

$$\mathrm{freqcontrol(connectx, y, z)} = |\{\{Sequences\ with\ \mathrm{connectx, y, z}\} \cap \{Sequences\ with\ \mathrm{control}\}|.$$

The total numbers of reads in the corresponding experiments are given by

$$\mathrm{readsprotein(connectx, y, z)} = |\{Sequences\ with\ \mathrm{protein}\}|,$$

$$\mathrm{readscontrol(connectx, y, z\,)} = |\{\mathit{Sequences\ with}\ \mathrm{control}\}|$$

Then the enrichment factor is calculated as

$$\mathrm{EFprotein, control(connectx, y, z)} = \frac{\mathrm{freqprotein(connectx, y, z\,)} * \mathrm{readscontrol}}{\mathrm{freqcontrol(connectx, y, z\,)} * \mathrm{readsprotein}}.$$

Visualization of the results

For visualization, it is useful to consider both enrichment factor and abundance of connectivities in a two-dimensional display. Since the abundance values can vary substantially, we use ranks to order connectivities by abundance:

$$RA(\mathrm{connectx, y, z}) = rank(\mathrm{connectx, y, z}\ |C).$$

Here, $RA(\mathrm{connectx, y, z})$ is the rank of $\mathrm{connectx, y, z}$ within all connectivities with abundance>0, where the rank is calculated also with respect to all proteins and control groups. The corresponding two-dimensional plot of enrichment factor and abundance allows the determination of interesting candidate molecules, which can be found in such a plot with large values for both quantities. Figures S7 show a plot for the selection experiments on streptavidin. The ECEC allows for highlighting selected building blocks to deduce structure-enrichment relationships from the plot.

A **B**

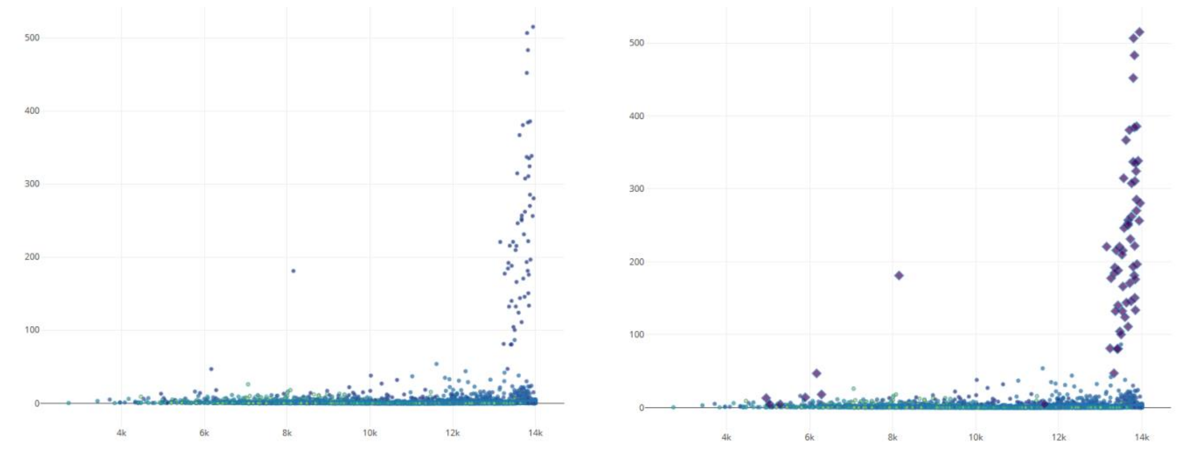

Figure S7: 2D plots of selection experiment on streptavidin beads generated by ECEC. A. Two-dimensional display of the results for the selection on streptavidin beads versus the control experiment on empty beads; the colouring variable is Building Block 2 (azides). B. Visualization of the selection experiment on streptavidin beads with highlighting of the barcode for a desthiobiotin building block.

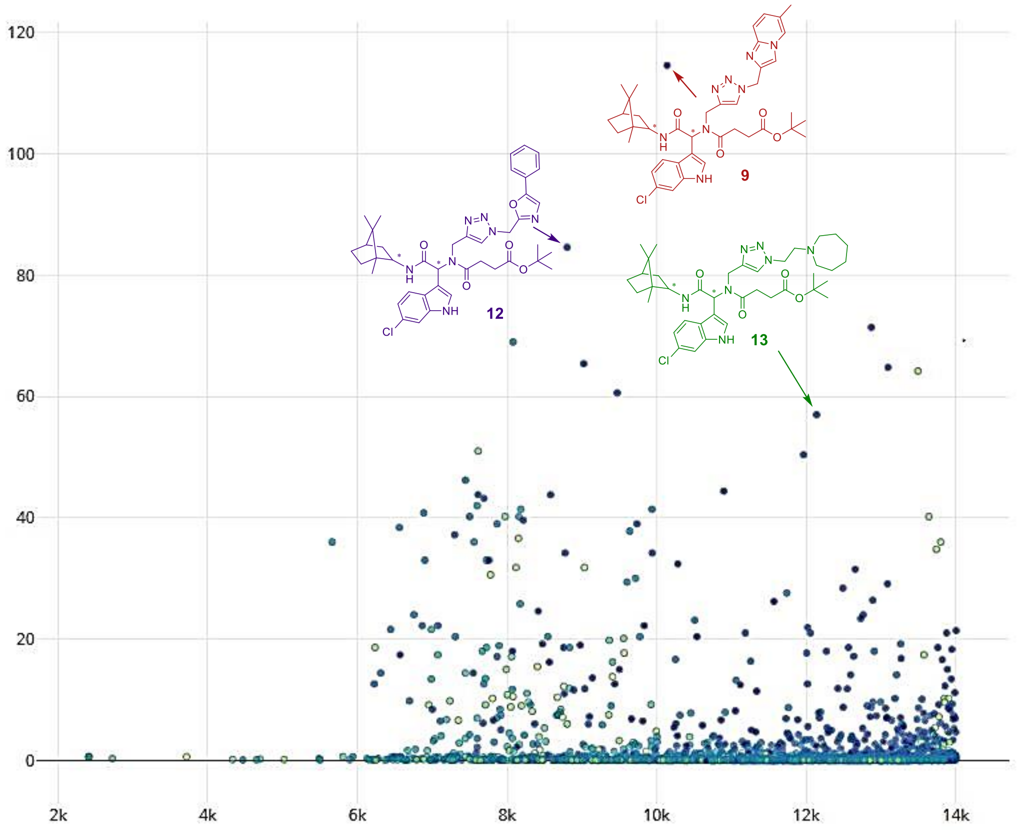

Figure S8: Plot of enrichment factors obtained after tiDEL selection on hTEAD4.

Synthesis and characterization of isocyanides I1-I18

General procedure

All isocyanides (**I1**,[2] **I2**,[3] **I3**,[3] **I5**,[4] **I6**,[5] **I7**,[5] **I8**,[6] **I9**,[6] **I12**,[7] **I13**,[8] **I14**,[9] **I16**,[2] **I17**,[10] **I18**[11]) were prepared in house by either performing the Ugi,[12] Hoffman[13] or Leukart-Wallach reductive amination procedure.[6]

Representative procedure (RP-04) for the synthesis of 1-ethyl-2-isocyanobenzene (I10)

To a solution of *N*-(2-ethylphenyl)formamide (73.8 mmol) in CH_2Cl_2 (200 mL), NEt_3 (370 mmol) was added and the mixture was cooled to -5 °C. $POCl_3$ (73.8 mmol) was added dropwise while keeping the temperature between -5 to 0 °C. Then, the reaction mixture was stirred for 2 h at room temperature. After completion of the reaction, an aqueous solution of Na_2CO_3 (0.6 M, 100 mL) was added carefully. Additional water was added until all salts were dissolved (~300 mL). The mixture was transferred to a separatory funnel and the organic layer was separated. The water layer was extracted with CH_2Cl_2 (5x 200 mL). The combined organic layers were washed with brine (100 mL), dried over $MgSO_4$, concentrated *in vacuo*, fast filtration over silica column (eluting with diethyl ether), to give the corresponding isocyanide in 91% yield.

Analytical data for some exemplary isocyanides

2-isocyano-1,7,7-trimethylbicyclo[2.2.1]heptane (I4)

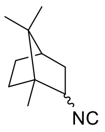

82% yield; light yellow solid; mixture of diastereoisomers (endo/exo 1:2) **^{1}H NMR** (500 MHz, $CDCl_3$): major diastereoisomer; δ = 3.52-3.36 (m, 1H), 2.17-2.01 (m, 1H), 1.98-1.24 (m, 6H), 1.07 (s, 3H), 1.06 (s, 3H), 0.87 ppm (s, 3H). **^{13}C NMR** (126 MHz, $CDCl_3$): mixture of diastereoisomers; δ = 155.3 (t, *J* = 5.3 Hz), 155.1 (t, *J* = 5.6 Hz), 60.1 (t, *J* = 5.5 Hz), 59.2 (t, *J* = 6.5 Hz), 49.9, 48.9, 47.7, 44.6, 44.5, 38.5, 37.3, 34.7, 34.6, 28.2, 27.6, 26.8, 19.9, 19.8, 19.7, 18.3, 13.1, 12.4 ppm.

1-ethyl-2-isocyanobenzene (I10)

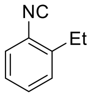

91% yield; dark orange oil; **^{1}H NMR** (500 MHz, $CDCl_3$): δ = 7.39-7.30 (m, 2H), 7.30-7.27 (m, 1H), 7.25-7.17 (m, 1H), 2.80 (q, *J* = 7.6 Hz, 2H), 1.27 ppm (t, *J* = 7.6 Hz, 3H). **^{13}C NMR** (126 MHz, $CDCl_3$): δ = 165.6 (t, *J* = 5.7 Hz), 140.6, 129.4, 128.9, 126.8, 126.6, 25.4, 13.8 ppm.

4-(isocyanomethyl)benzonitrile (I11)

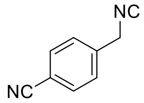

32% yield; orange solid; **^{1}H NMR** (500 MHz, $CDCl_3$): δ = 7.72 (d, *J* = 8.5 Hz, 2H), 7.51 (d, *J* = 8.5 Hz, 2H), 4.77 ppm (s, 2H). **^{13}C NMR** (126 MHz, $CDCl_3$): δ = 159.1, 137.1, 132.5, 127.0, 118.0, 112.1, 44.9 ppm (t, *J* = 7.5 Hz).

1-(1-isocyanoethyl)-4-methoxybenzene (I15)

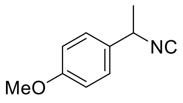

44% yield; orange semi-solid; **^{1}H NMR** (500 MHz, $CDCl_3$): δ = 7.27 (d, *J* = 8.7 Hz, 2H), 6.90 (d, *J* = 8.7 Hz, 2H), 4.86-4.66 (m, 1H), 3.80 (s, 3H), 1.72-1.54 ppm (m, 3H). **^{13}C NMR** (126 MHz, $CDCl_3$): δ = 159.3, 155.7 (t, *J* = 4.8 Hz), 130.6, 126.5, 114.1, 55.2, 53.1 (t, *J* = 6.2 Hz), 24.9 ppm.

Synthesis and characterization of compounds 8-15

Representative procedure for the Ugi four-component condensation (RP-05)

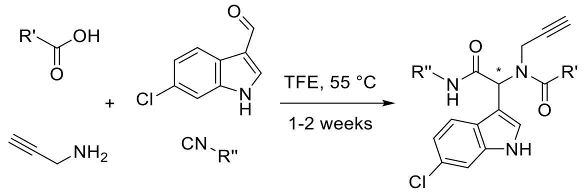

Carboxylic acid (1 equiv., 0.17 mmol), propargylamine (1 equiv., 10.9 µL, 0.17 mmol), 6-chloro-1*H*-indole-3-carbaldehyde (1 equiv., 30.9 mg, 0.17 mmol) und isocyanide (1 equiv., 0.17 mmol) were suspended in 200 µL 2,2,2-trifluoroethanol. The suspension was stirred at 55 °C for 1-2 weeks. Every third day fresh 2,2,2-trifluoroethanol (200 µL) was added. The solvent was removed *in vacuo* and the crude mixture was charged onto silica gel and the product was purified using petroleum ether (40 – 60 °C) / ethyl acetate as eluent.

Representative procedure for the copper-catalyzed azide-alkyne cycloaddition (RP-06)

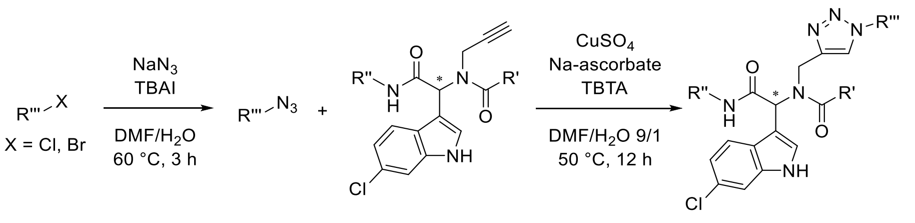

To the solution of an alkyl or benzyl halide (1 equiv., 0.041 mmol) in 400 µL DMF were added sodium azide (1.2 equiv., 3.2 mg, 0.049 mmol) dissolved in 200 µL H_2O and tetrabutylammonium iodide (TBAI, 20 mol%, 3.1 mg, 0.008 mmol) dissolved in 100 µL DMF. The resulting solution was stirred at 60 °C for 3 h. Afterwards, the Ugi-reaction product (1.2 equiv., 0.049 mmol) was dissolved in 400 µL DMF, tris(benzyltriazolylmethyl)amine (TBTA, 10 mol%, 2.2 mg, 0.004 mmol) dissolved in 200 µL DMF and sodium ascorbate (25 mol%, 2.1 mg, 0.01 mmol) and copper(II) sulfate (5 mol%, 0.5 mg, 0.002 mmol), each dissolved in 200 µL H_2O, were added sequentially. The reaction mixture was stirred at 50 °C for 12 h. The reaction mixture was then diluted with cold water (10 mL). The aqueous layer was extracted with EtOAc (3x 10 mL). The combined organic layers were washed with 1 M EDTA solution (10 mL) and brine (10 mL), dried over anhydrous $MgSO_4$, filtered and concentrated *in vacuo*. The triazole product was isolated by silica gel column chromatography using MeOH in DCM as eluent.

Analytical data for hit compounds 8-15

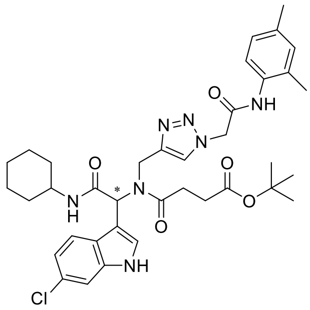

***tert*-butyl 4-((1-(6-chloro-1*H*-indol-3-yl)-2-(cyclohexylamino)-2-oxoethyl)((1-(2-((2,4-dimethyl-phenyl)amino)-2-oxoethyl)-1*H*-1,2,3-triazol-4-yl)methyl)amino)-4-oxobutanoate (8)**

Compound **8** was prepared by Ugi-reaction according to the representative procedure RP-05 from mono-*tert*-butyl succinate (30 mg, 0.17 mmol), propargylamine (10.9 µL, 0.17 mmol), 6-chloro-1*H*-indole-3-carbaldehyde (30.9 mg, 0.17 mmol) and isocyanocyclohexane (21.4 µL, 0.17 mmol). The product was isolated with 39% yield as a slightly orange mixture with unreacted 6-chloro-1*H*-indole-3-carbaldehyde (ratio 1:1) and was used without further purification. The azide was prepared from 2-bromo-*N*-(2,4-dimethylphenyl)acetamide (10 mg, 0.041 mmol) and was used for the azide-alkyne cycloaddition with the Ugi-product (24.5 mg, 0.049 mmol) according to representative procedure RP-06. The desired product **8** was isolated with 44% yield as a colorless, amorphous solid.

^{1}H NMR: (400 MHz, $CDCl_3$): δ = 8.30 (s, 1H), 7.50 – 7.45 (m, 1H), 7.37 – 7.34 (m, 1H), 7.31 – 7.26 (m, 2H), 7.24 (s, 1H), 7.11 (s, 1H), 7.01 – 6.96 (m, 1H), 6.95 (s, 1H), 6.47 (s, 1H), 5.53 (s, 1H), 4.26 (s, 1H), 2.81 – 2.63 (m, 3H), 2.26 (s, 3H), 2.06 (s, 2H), 1.80 (d, J = 10.6 Hz, 2H), 1.69 – 1.52 (m, 4H), 1.46 (s, 6H), 1.25 (s, 9H), 1.19 – 1.04 ppm (m, 4H); ^{13}C NMR (101 MHz, $CDCl_3$): δ = 173.30, 172.72, 136.23, 135.97, 134.14, 131.40, 129.36, 129.12, 128.59, 128.26, 127.88, 127.36, 124.50, 123.60, 120.96, 119.77, 111.46, 80.84, 54.67, 53.23, 46.19, 40.63, 32.87, 32.06, 30.76, 29.83, 29.50, 29.03, 28.24, 25.56, 24.91, 22.83, 21.01, 17.67, 14.26 ppm; RP-HPLC-MS (I): t_R = 6.8 min, $[M+H]^+$ = 704.1; HRMS (ESI) calculated for $C_{37}H_{47}O_5N_7{}^{35}Cl$ $[M+H]^+$ = 704.33217, found 704.33200; calculated for $C_{37}H_{47}O_5N_7{}^{37}Cl$ $[M+H]^+$ = 706.32922, found 706.32946.

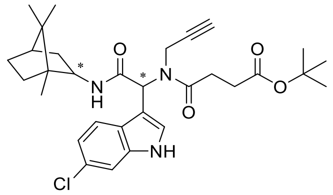

***tert*-butyl 4-((1-(6-chloro-1*H*-indol-3-yl)-2-oxo-2-(((1*S*,2*R*,4*R*)-1,7,7-trimethylbicyclo-[2.2.1]heptan-2-yl)amino)ethyl)(prop-2-yn-1-yl)amino)-4-oxobutanoate (15)**

Compound **15** was prepared by Ugi-reaction according to the representative procedure RP-05 from mono-*tert*-butyl succinate (30 mg, 0.17 mmol), propargylamine (10.9 µL, 0.17 mmol), 6-chloro-1*H*-indole-3-carbaldehyde (30.9 mg, 0.17 mmol) and 2-isocyano-1,7,7-trimethylbicyclo[2.2.1]heptane (32.0 µL, 0.17 mmol). Product **15** was isolated with 44% yield as an amorphous solid and a mixture of diastereomers (ratio 2:1).

^{1}H NMR (500 MHz, $CDCl_3$): δ = 8.54 (m, 1H), 7.70 – 7.61 (m, 1H), 7.40 – 7.32 (m, 3H), 7.08 – 7.03 (m, 2H), 6.48 (d, J = 10.0 Hz, 1H), 5.30 (s, 1H), 4.06 (s, 1H), 4.05 – 4.02 (m, 2H), 2.83 – 2.76 (m, 3H), 2.71 – 2.64 (m, 3H), 2.59 (t, J = 6.7 Hz, 1H), 2.45 (t, J = 6.7 Hz, 1H), 1.95 (d, J = 5.7 Hz, 1H), 1.81 (dd, J = 13.1, 9.2 Hz, 1H), 1.73 – 1.70 (m, 3H), 1.73 – 1.55 (m, 4H), 1.47 (s, 9H), 1.44 (s, 5H), 1.30 – 1.23 (m, 2H), 1.18 – 1.11 (m, 2H), 0.88 – 0.84 (m, 4H), 0.84 – 0.78 ppm (m, 9H);^{13}C NMR (126 MHz, $CDCl_3$): δ = 173.07, 173.03, 172.40, 172.25, 128.68, 128.60, 127.01, 125.89, 125.79, 121.02, 120.95, 120.06, 111.35, 111.32, 81.17, 80.72, 72.22, 72.01, 71.73, 48.83, 47.19, 44.96, 38.87, 38.78, 36.16, 36.05, 31.18, 30.82, 30.61, 29.39, 28.94, 28.26, 28.20, 27.16, 20.35, 20.31, 12.04 ppm; RP-HPLC-MS (I): t_R = 9.1 min, $[M+H]^+$ = 553.8; HRMS (ESI) calculated for $C_{31}H_{41}O_4N_3{}^{35}Cl$ $[M+H]^+$ = 554.27801, found 554.27783; calculated for $C_{31}H_{41}O_4N_3{}^{37}Cl$ $[M+H]^+$ = 556.27506, found 556.27504.

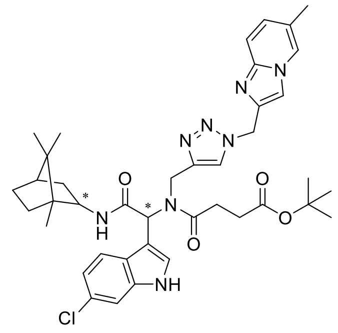

tert-butyl 4-((1-(6-chloro-1_H_-indol-3-yl)-2-oxo-2-(((1_S_,2_R_,4_R_)-1,7,7-trimethylbicyclo-[2.2.1]heptan-2-yl)amino)ethyl)((1-((6-methylimidazo[1,2-a]pyridin-2-yl)methyl)-1H-1,2,3-triazol-4-yl)methyl)amino)-4-oxobutanoate (9)

The azide was prepared from 2-(chloromethyl)-6-methylimidazo[1,2-a]pyridine hydrochloride (12 mg, 0.055 mmol) with sodium azide (7.9 mg, 0.120 mmol, 2.2 equiv.) and was used for the azide-alkyne cycloaddition with compound **15** (36.4 mg, 0.066 mmol) according to representative procedure RP-06. The desired product **9** was isolated with 56% yield as a colorless, amorphous solid and a mixture of diastereomers (ratio 1:1).

^{1}H NMR (400 MHz, CD_2Cl_2): δ = 8.94 (s, 1H), 8.91 (s, 1H), 7.92 (s, 2H), 7.55 (dd, *J* = 7.6, 2.1 Hz, 2H), 7.47 – 7.30 (m, 6H), 7.30 – 7.23 (m, 2H), 7.18 (d, *J* = 1.7 Hz, 1H), 7.12 – 7.05 (m, 3H), 6.98 – 6.92 (m, 3H), 6.66 (s, 1H), 6.51 (d, *J* = 8.6 Hz, 1H), 6.44 – 6.35 (m, 3H), 5.27 (d, *J* = 4.9 Hz, 1H), 4.61 – 4.50 (m, 4H), 3.87 – 3.74 (m, 2H), 2.81 – 2.71 (m, 4H), 2.62 – 2.55 (m, 4H), 2.32 (s, 1H), 2.31 (s, 1H), 1.69 (s, 6H), 1.59 (s, 5H), 1.44 (s, 19H), 1.33 – 1.11 (m, 7H), 0.86 – 0.72 ppm (m, 18H); ^{13}C NMR (151 MHz, CD_2Cl_2) δ 173.78, 173.09, 173.00, 170.71, 170.56, 169.76, 169.63, 145.51, 145.45, 145.33, 139.92, 139.89, 136.70, 136.67, 136.56, 136.42, 132.85, 132.71, 129.59, 128.74, 128.24, 128.15, 128.09, 127.97, 127.65, 127.57, 127.50, 126.48, 126.02, 125.99, 123.28, 123.18, 123.10, 120.75, 120.66, 120.54, 120.17, 113.56, 113.51, 113.43, 111.35, 111.33, 111.17, 110.04, 110.00, 109.84, 80.86, 80.83, 80.81, 57.61, 57.48, 54.75, 54.68, 50.09, 50.03, 49.22, 49.20, 48.67, 48.64, 47.48, 47.46, 45.50, 45.48, 44.08, 44.05, 43.97, 43.89, 40.70, 40.63, 40.58, 39.18, 39.02, 37.38, 37.34, 36.53, 36.52, 31.23, 31.17, 31.15, 29.62, 29.59, 29.56, 28.56, 28.40, 28.27, 27.51, 20.52, 20.40, 20.05, 18.91, 18.89, 18.43, 18.42, 14.14, 14.12, 12.26, 12.08 ppm; RP-HPLC-MS (I): t_R = 8.4 min, $[M+H]^+$ = 741.1; HRMS (ESI) calculated

for $C_{40}H_{50}O_4N_8{}^{35}Cl$ $[M+H]^+$ = 741.36381, found 741.36420; calculated for $C_{40}H_{50}O_4N_8{}^{37}Cl$ $[M+H]^+$ = 743.36086, found 743.36061.

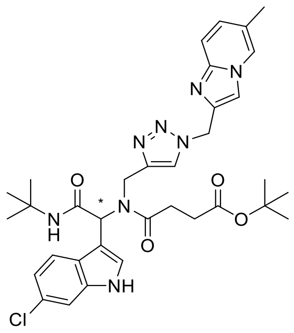

tert-butyl 4-((2-(_tert_-butylamino)-1-(6-chloro-1_H_-indol-3-yl)-2-oxo-1l3-ethyl)((1-((6-methylimidazo-[1,2-a]pyridin-2-yl)methyl)-1_H_-1,2,3-triazol-4-yl)methyl)amino)-4-oxo-butanoate (10)

Compound **10** was prepared by Ugi-reaction according to the representative procedure RP-05 from mono-*tert*-butyl succinate (77.6 mg, 0.44 mmol), propargylamine (28.5 µL, 0.44 mmol), 6-chloro-1*H*-indole-3-carbaldehyde (80.0 mg, 0.44 mmol) and *tert*-butyl isocyanide (99.6 µL, 0.44 mmol). The Ugi product was isolated with 50% yield as a slightly orange mixture with unreacted 6-chloro-1*H*-indole-3-carbaldehyde (ratio 9:1) and was used without further purification. The azide was prepared from 2-(chloromethyl)-6-methylimidazo[1,2-a]pyridine hydrochloride (40.0 mg, 0.18 mmol) with sodium azide (18.0 mg, 0.27 mmol, 1.5 equiv.) and was used for the azide-alkyne cycloaddition with the Ugi-product (103.75 mg, 0.22 mmol) according to representative procedure RP-06. The desired product **10** was isolated with 32% yield as a colorless, amorphous solid.

^1H NMR (500 MHz, $CDCl_3$) δ 9.78 (s, 1H), 8.01 (s, 1H), 7.63 – 7.58 (m, 1H), 7.45 (s, 1H), 7.38 (s, 1H), 7.30 – 7.27 (m, 1H), 7.22 (d, J = 8.5 Hz, 1H), 7.13 (s, 1H), 6.83 (dd, J = 8.5, 1.6 Hz, 1H), 6.63 (s, 1H), 6.43 (s, 1H), 5.40 – 5.29 (m, 2H), 4.63 (d, J = 4.4 Hz, 2H), 2.85 – 2.78 (m, 2H), 2.66 – 2.61 (m, 2H), 2.38 (s, 3H), 1.44 (s, 9H), 1.32 (s, 9H); ^{13}C NMR (126 MHz, $CDCl_3$) δ 173.69, 172.87, 169.50, 145.29, 136.39, 128.14, 127.18, 126.15, 124.86, 122.67, 120.75, 120.05, 111.50, 110.13, 80.72, 51.81, 40.91, 31.10, 29.43, 28.96, 28.48, 18.50 ppm; RP-HPLC-MS (I): t_R = 4.6 min, $[M+H]^+$ = 661.1; HRMS (ESI) calculated for $C_{34}H_{42}O_4N_8{}^{35}Cl$ $[M+H]^+$ = 661.30121, found 661.30143; calculated for $C_{34}H_{42}O_4N_8{}^{37}Cl$ $[M+H]^+$ = 663.29826, found 663.29927.

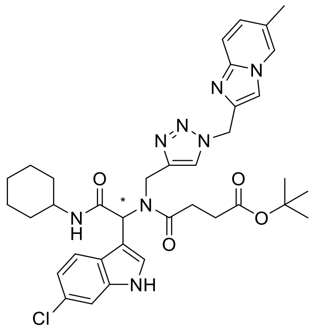

tert-butyl 4-((1-(6-chloro-1_H_-indol-3-yl)-2-(cyclohexylamino)-2-oxo-1l3-ethyl)((1-((6-methyl-imidazo[1,2-a]pyridin-2-yl)methyl)-1_H_-1,2,3-triazol-4-yl)methyl)amino)-4-oxo-butanoate (11)

Compound **11** was prepared by Ugi-reaction according to the representative procedure RP-05 from mono-*tert*-butyl succinate (67.9 mg, 0.39 mmol), propargylamine (25.0 µL, 0.39 mmol), 6-chloro-1*H*-indole-3-carbaldehyde (70.0 mg, 0.39 mmol) and cyclohexyl isocyanide (48.4 µL, 0.39 mmol). The Ugi product was isolated with 43% yield as a slightly orange mixture with unreacted 6-chloro-1*H*-indole-3-carbaldehyde (ratio 1:1) and was used without further purification. The azide was prepared from 2-(chloromethyl)-6-methylimidazo[1,2-a]pyridine hydrochloride (30.0 mg, 0.14 mmol) with sodium azide (13.48 mg, 0.21 mmol, 1.5 equiv.) and was used for the azide-alkyne cycloaddition with the Ugi-product (82.09 mg, 0.16 mmol) according to representative procedure RP-06. The desired product **11** was isolated with 40% yield as a colorless, amorphous solid.

^{1}H NMR (500 MHz, $CDCl_3$) δ 9.87 (s, 1H), 8.07 (s, 1H), 7.73 (d, J = 9.2 Hz, 1H), 7.51 (d, J = 2.1 Hz, 1H), 7.45 (s, 1H), 7.40 (d, J = 9.3 Hz, 1H), 7.21 (d, J = 8.5 Hz, 1H), 7.15 (d, J = 1.5 Hz, 1H), 6.80 (dd, J = 8.5, 1.7 Hz, 1H), 6.59 (s, 1H), 6.54 (s, 1H), 5.40 (dd, J = 37.7, 15.5 Hz, 2H), 4.62 (dd, J = 49.2, 17.0 Hz, 2H), 2.93 – 2.56 (m, 5H), 2.41 (s, 3H), 1.87 (t, J = 14.7 Hz, 2H), 1.68 (dd, J = 9.7, 3.7 Hz, 2H), 1.58 (dd, J = 8.6, 4.2 Hz, 1H), 1.45 (s, 9H), 1.32 – 1.09 (m, 7H); ^{13}C NMR (126 MHz, $CDCl_3$) δ 173.56, 172.82, 168.90, 145.01, 136.10, 127.76, 127.03, 125.93, 124.92, 122.56, 120.48, 119.80, 114.57, 112.22, 111.22, 109.77, 80.62, 53.65, 48.79, 40.64, 32.89, 31.01, 29.11, 28.24, 25.61, 25.04, 24.99, 18.27.; RP-HPLC-MS (I): t_R = 4.7 min, $[M+H]^+$ = 687.2; HRMS (ESI) calculated for $C_{36}H_{44}O_4N_8{}^{35}Cl$ $[M+H]^+$ = 687.31686, found 687.31676.

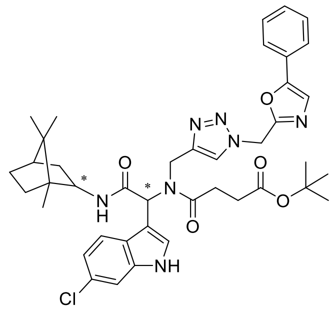

***tert*-butyl 4-((1-(6-chloro-1*H*-indol-3-yl)-2-oxo-2-(((1*S*,4*R*)-1,7,7-trimethylbicyclo[2.2.1]-heptan-2-yl)amino)-1l3-ethyl)((1-((5-phenyloxazol-2-yl)methyl)-1*H*-1,2,3-triazol-4-yl)-methyl)amino)-4-oxobutanoate (12)**

The azide was prepared 2-(chloromethyl)-5-phenyloxazole (5.0 mg, 0.026 mmol) with sodium azide (2.5 mg, 0.038 mmol, 1.5 equiv.) and was used for the azide-alkyne cycloaddition with compound **15** (17.0 mg, 0.031 mmol) according to representative procedure RP-06. The desired product **12** was isolated with 76% yield as a colorless, amorphous solid and a mixture of diastereomers (ratio: 2:1).

^{1}H NMR (500 MHz, CD_2Cl_2): δ = 8.74 (d, *J* = 17.3 Hz, 1H), 7.63 – 7.59 (m, 4H), 7.43 (t, *J* = 7.6 Hz, 4H), 7.36 (t, *J* = 7.4 Hz, 2H), 7.32 (d, *J* = 2.1 Hz, 2H), 7.30 (d, *J* = 1.8 Hz, 1H), 7.29 – 7.27 (m, 1H), 7.26 (d, *J* = 2.8 Hz, 1H), 7.01 – 6.97 (m, 2H), 6.41 (d, *J* = 8.4 Hz, 1H), 6.38 (d, *J* = 6.5 Hz, 1H), 5.42 (s, 1H), 5.38 (s, 1H), 5.33 (d, *J* = 1.4 Hz, 1H), 4.61 (dd, *J* = 16.1, 5.8 Hz, 2H), 4.53 (dd, *J* = 17.3, 7.2 Hz, 1H), 2.79 – 2.52 (m, 7H), 1.79 – 1.66 (m, 5H), 1.58 – 1.49 (m, 2H), 1.44 (s, 5H), 1.44 (s, 9H), 1.27 – 1.09 (m, 4H), 0.83 (s, 2H), 0.80 – 0.77 (m, 7H), 0.74 (s, 3H); ^{13}C NMR (126 MHz, CD_2Cl_2): δ = 173.70, 172.83, 172.73,

169.46, 169.38, 156.69, 156.64, 153.30, 153.24, 145.94, 145.84, 136.57, 136.40, 129.53, 129.50, 128.63, 128.58, 127.82, 127.80, 127.69, 127.59, 126.38, 126.33, 124.86, 123.16, 123.13, 122.95, 121.04, 121.02, 120.33, 120.30, 111.71, 111.66, 110.16, 110.09, 80.82, 80.74, 57.41, 57.37, 49.24, 49.14, 47.47, 47.43, 47.01, 46.90, 45.45, 41.24, 40.97, 39.05, 39.02, 36.51, 36.46, 31.08, 30.99, 29.50, 28.36, 27.50, 20.53, 20.50, 20.40, 20.37, 20.06, 14.17, 14.12, 12.24, 12.09 ppm; RP-HPLC-MS (II): t_R = 11.2 min, $[M+H]^+$ = 754.0; HRMS (ESI) calculated for $C_{41}H_{49}O_5N_7{}^{35}Cl$ $[M+H]^+$ = 754.34782, found 754.34665; calculated for $C_{41}H_{49}O_5N_7{}^{37}Cl$ $[M+H]^+$ = 756.34487, found 756.34439.

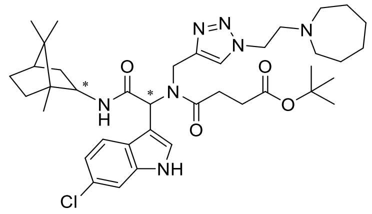

***tert*-butyl 4-(((1-(2-(azepan-1-yl)ethyl)-1*H*-1,2,3-triazol-4-yl)methyl)(1-(6-chloro-1*H*-indol-3-yl)-2-oxo-2-(((1*S*,2*R*,4*R*)-1,7,7-trimethylbicyclo[2.2.1]heptan-2-yl)amino)ethyl)amino)-4-oxobutanoate (13)**

The azide was prepared from 1-(2-chloroethyl)azepane hydrochloride (10 mg, 0.050 mmol) with sodium azide (7.2 mg, 0.110 mmol, 2.2 equiv.) and was used for the azide-alkyne cycloaddition with Ugi-product **15** (33.2 mg, 0.060 mmol) according to representative procedure RP-06. The desired product **13** was isolated with 76% yield as a colorless, amorphous solid and a mixture of diastereomers (ratio 2:1).

^{1}H NMR (500 MHz, CD_2Cl_2): δ = 7.62 (s, 1H), 7.57 (s, 1H), 7.37 – 7.35 (m, 2H), 7.31 (dd, *J* = 8.5, 2.3 Hz, 2H), 7.02 (d, *J* = 8.3 Hz, 2H), 6.94 (s, 1H), 6.68 (s, 1H), 6.46 (s, 1H), 6.43 (s, 1H), 4.64 – 4.58 (m, 3H), 4.13 – 3.96 (m, 5H), 3.90 – 3.82 (m, 2H), 3.24 – 3.18 (m, 3H), 2.73 – 2.64 (m, 6H), 2.63 – 2.55 (m, 12H), 1.80 – 1.75 (m, 2H), 1.72 – 1.70 (m, 3H), 1.67 – 1.62 (m, 5H), 1.46 (s, 6H), 1.45 (s, 9H), 1.44 – 1.40 (m, 5H), 1.30 – 1.25 (m, 4H), 1.19 – 1.12 (m, 3H), 0.87 – 0.85 (m, 3H), 0.83 – 0.78 ppm (m, 12H); ^{13}C NMR (126 MHz, CD_2Cl_2): δ = 122.83, 120.41, 57.46, 55.70, 55.65, 49.21, 47.49, 47.44, 45.47, 41.43, 41.14, 39.03, 36.56, 36.49, 31.07, 29.45, 28.86, 28.38, 27.52, 24.57, 20.54, 20.27, 13.94, 12.24, 12.15 ppm; RP-HPLC-MS (II): t_R = 7.7 min, $[M+H]^+$ = 722.2; HRMS (ESI) calculated for $C_{39}H_{57}O_4N_7{}^{35}Cl$ $[M+H]^+$ = 722.41551, found 722.41544; calculated for $C_{39}H_{57}O_4N_7{}^{37}Cl$ $[M+H]^+$ = 724.41256, found 724.41399.

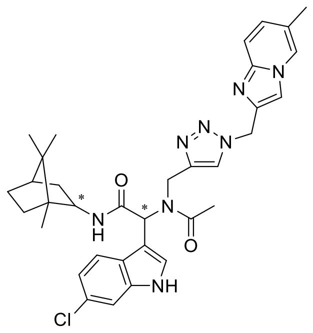

2-(6-chloro-1*H*-indol-3-yl)-2-(*N*-((1-((6-methylimidazo[1,2-a]pyridin-2-yl)methyl)-1*H*-1,2,3-triazol-4-yl)methyl)acetamido)-*N*-((1*S*,2*R*,4*R*)-1,7,7-trimethylbicyclo[2.2.1]heptan-2-yl)acetamide (14)

Compound **14** was prepared by Ugi-reaction according to the representative procedure RP-05 from acetic acid (9.5 µL, 0.17 mmol), propargylamine (10.9 µL, 0.17 mmol), 6-chloro-1*H*-indole-3-carbaldehyde (30.9 mg, 0.17 mmol) and 2-isocyano-1,7,7-trimethylbicyclo-[2.2.1]heptane (32.0 µL, 0.17 mmol). The Ugi product was isolated with 41% yield as a slightly orange mixture with unreacted 6-chloro-1*H*-indole-3-carbaldehyde (ratio 1:1) and was used without further purification. The azide was prepared from 2-(chloromethyl)-6-methylimidazo[1,2-a]pyridine hydrochloride (12 mg, 0.055 mmol) with sodium azide (7.9 mg, 0.120 mmol, 2.2 equiv.) and was used for the azide-alkyne cycloaddition with the Ugi-product (28.9 mg, 0.066 mmol) according to representative procedure RP-06. The desired product **14** was isolated with 47% yield as a colorless, amorphous solid and a mixture of diastereomers (ratio 1:1).

^{1}H NMR (600 MHz, MeOD): δ = 8.21 (s, 2H), 7.59 (s, 1H), 7.56 (s, 1H), 7.44 – 7.40 (m, 2H), 7.34 (s, 1H), 7.31 (s, 1H), 7.26 – 7.22 (m, 5H), 7.19 – 7.14 (m, 2H), 6.94 (s, 1H), 6.85 (s, 1H), 6.84 – 6.81 (m, 2H), 6.35 (s, 1H), 6.31 (s, 1H), 5.42 – 5.28 (m, 4H), 4.75 – 4.63 (m, 3H), 3.85 – 3.77 (m, 1H), 2.35 (s, 6H), 2.29 (s, 3H), 2.27 (s, 3H), 1.77 – 1.53 (m, 10H), 1.27 – 1.11 (m, 4H), 0.82 (s, 3H), 0.82 – 0.80 (m, 6H), 0.79 (s, 3H), 0.79 ppm (s, 6H); ^{13}C NMR (151 MHz, MeOD): δ = 174.69, 174.53, 172.36, 172.30, 146.37, 146.19, 145.79, 140.22, 140.15, 137.92, 137.82, 130.78, 128.89, 128.45, 128.19, 127.08, 127.05, 125.86, 124.42, 123.53, 123.41, 121.06, 120.99, 120.57, 120.51, 116.75, 112.86, 112.24, 112.15, 110.34, 110.29, 58.67, 58.63, 56.20, 55.61, 50.57, 50.12, 47.88, 47.83, 46.31, 46.29, 42.96, 42.80, 38.66, 38.35, 37.23, 37.11, 28.01, 22.65, 22.62, 20.79, 20.36, 20.25, 20.21, 20.17, 19.01, 17.95, 14.49, 14.37, 12.41, 12.15 ppm; RP-HPLC-MS (I): t_R = 4.4 min, $[M+H]^+$ = 627.1; HRMS (ESI) calculated for $C_{34}H_{40}O_2N_8{}^{35}Cl$ $[M+H]^+$ = 627.29573, found 627.29591; calculated for $C_{34}H_{40}O_2N_8{}^{37}Cl$ $[M+H]^+$ = 629.29573, found 629.29346.

Synthesis and characterization of YAP peptide fragment YAP$^{50\text{-}100}$

Peptides were synthesized on a Syro I peptide synthesizer (Multisyntech) following standard Fmoc-protocols for solid-phase peptide synthesis. Peptide synthesis was performed on Rink amide ChemMatrix resin (Aldrich, 0.28 mmol/g, 0.05 mmol). To prepare the solutions, the amino acids were dissolved in a solution of oxyma pur 0.5 M in DMF to obtain a final concentration of 0.5 M, HATU was dissolved in DMF to get a concentration of 0.5 M, DIPEA was dissolved in NMP to get a concentration

of 2 M, piperidine was dissolved in DMF with a concentration of 25% and acetic anhydride was dissolved in NMP with a concentration of 10%. For the coupling, 400 µL of amino acids solution (0.2 mmol, 4 equiv.) were mixed with 400 µL of HATU solution (0.2 mmol, 4 equiv.) and 200 µL of DIPEA solution (0.4 mmol, 8 equiv.) and added to the resin for 40 min (each amino acid was coupled twice or three times). The amino acid couplings were followed by a capping of the remaining free amino groups with 800 µL of Ac_2O solution in presence of 200 µL of DIPEA solution for 2 min. Fmoc-deprotection was performed with 25% piperidine in DMF for 10 min. **YAP$^{50\text{-}100}$** was obtained after peptide elongation of the corresponding amino acid sequence:
(AGHQIVHVRGD**S**E**TDL**EALFNAVMNPKTANVPQTV**PMR**L**R**KLPDSFFKP**P**E (residues in bold were triply coupled and underlined residues were doubly coupled). The resulting peptide resin was washed six times with 2 mL of DMF between each step. The *N*-terminal Fmoc group was deprotected using 2 mL of 25% piperidine in DMF (3x 5 min) followed by washing steps with DMF (3x 6 mL) and CH_2Cl_2 (3x 6 mL). 76 mg of Fmoc-O_2Oc-OH (0.2 mmol, 4 equiv.), 76 mg of HATU (0.2 mmol, 4 equiv.) were dissolved in 400 µL of DMF, followed by 70 µL of DIPEA (0.4 mmol, 8 equiv.). The mixture was then added to the resin and stirred for 2 h. The peptide resin was washed with DMF (3x 6 mL) and CH_2Cl_2 (3x 6 mL). The *N*-terminal Fmoc group was deprotected using 2 mL of 25% piperidine in DMF (3x 5 min) followed by washing steps with DMF (3x 6 mL) and CH_2Cl_2 (3x 6 mL). A solution of 0.10 g 5-isothiocyanatofluorescein (0.25 mmol, 5 equiv.) and 90 µL of DIPEA (0.5 mmol, 10 equiv.) in 800 µL DMF. The reaction mixture was shaken in the dark at room temperature for 3 h. The resin was washed with DMF (3x 6 mL) and CH_2Cl_2 (3x 6 mL). The coupling procedure was repeated an additional time and this time it was shaken in the dark at room temperature for 16 h. The resulting bright yellow resin was washed with DMF (3x 6 mL) and CH_2Cl_2 (3x 6 mL) and dried under vacuum filtration. The peptidyl resin (0.05 mmol, 1 equiv.), was cleaved with 1 mL of cleavage solution (triisopropylsilane 2.5%, water 2.5% in TFA) for 3 hours at room temperature. The peptide was precipitated in 40 mL of cold diethyl ether/petroleum ether 1:1 after 10 minutes of centrifugation. The pellet was washed twice with 40 mL of diethyl ether. The residue was dried over vacuum, dissolved in water and lyophilized. The residue was dissolved in 1 mL of DMSO. Peptides were cleaved from the resin with TFA/H_2O/TIPS (95/2.5/2.5) for 3 h and precipitated with diethyl ether 0 °C. The crude peptide was purified using preparative RP-HPLC (Agilent 1290 infinity hyphenated with 6120 quadrupole Mass spectrometer) using Macherey-Nagel C_{18} gravity, 5 µm, 125x10 mm column. The purification was done with a flow rate of 20 mL/min in a gradient of 10% B to 100% B in 45 min (A = H_2O + 0.1% TFA, B = ACN + 0.1% TFA, detection at 214 nm, 220 nm, 254 nm and 280 nm and MS detection). The purification yielded 2.2 mg of FITC-YAP with a HPLC purity of 95%. Obs. $[M+4H]^{4+}$= 1558.9, Calc. $[M+4H]^{4+}$= 1558.5.
FITC-YAP purity was checked by analytical RP-HPLC (Agilent 1290/1260 infinity HPLC), using EC 100/2 NUCLEODUR C_{18} gravity column, 1.8 µm from Macherey-Nagel. The analysis was done at 1 mL/min in a gradient of 5% B and 1% C to 65% B and 1% C in 30 min (A = H_2O, B = ACN, C = H_2O + 10% TFA, detection at 214 nm).

Purity mass spectra was recorded on LC-MS Thermo FLEET from Thermo Fisher Scientific with a gradient of 5% B to 95% B in 8 min (A = H_2O + 0.1 % TFA, B = ACN + 0.1% TFA, detection at 214 nm).

FITC-YAP stock solution was prepared in DMSO. Solution concentration was measured at 495 nm in 100 mM sodium phosphate buffer (pH = 8.5) and calculated with an extinction coefficient of 77.000 $M^{-1}\cdot cm^{-1}$ in JASCO V-550.

Evaluation of compound 8 as MDM2 binder

Docking of compound 8 into the crystal structure of MDM2

Method: The modeling was performed using MOLOC software[14]. The crystal structure of MDM2 interacting with the p53 peptide was used (PDB ID 1YCR)[15]. First the p53 peptide was removed from the structure. The indole part of **8** was modelled into the MDM2 pocket in a way that p53 Trp23 indole were aligned. Energy minimization was performed. Then, the whole structure MDM2 and **8** was minimized.

Expression and purification of MDM2

The plasmid (pETM-13) containing MDM2 was transformed into *E. coli* BL21(DE3) using heat shock. MDM2 was expressed in LB medium supplemented with 50 µg/L kanamycin and 35 µg/L chloramphenicol. Induction was performed at an OD_{600} = 0.6 nm with 1 mM IPTG prior to incubation of the cultures at 18 °C overnight. Cells were harvested by centrifugation at 5000 rpm for 15 min. The cell pellet was resuspended in PBS buffer (pH = 7.4) containing 10 mM dithiothreitol (DTT) and protease-inhibitor cocktail (Protease Inhibitor Cocktail Set II, MERCK) and lysed by sonication for 10 min at 30% sonication power (Branson 250 Digital Sonifier). The lysate was cleared by centrifugation at 12,000 rpm, 4 °C.

The collected IBs (inclusion bodies) were washed three times, twice with washing buffer 1 (PBS, pH = 7.4, 10 mM DTT, 0.05% Triton) and the third time with washing buffer 2 (PBS, pH = 7.4, 10 mM DTT). Protein was then denatured in denaturation buffer (100 mM Tris, pH = 8.0, 6 M guanidine-hydrochloride, 1 mM EDTA and 10 mM *β*-mercaptoethanol) and dialysis was performed against the dialysis buffer (4 M guanidine hydrochloride ,pH = 3.5, 10 mM *β*-mercaptoethanol) for 6 h at 4 °C. Next, protein was diluted 100-fold into refolding buffer (10 mM Tris base, pH = 7.0, 1 mM EDTA, 10 mM DTT) and incubated under stirring overnight at 4 °C.

The protein was incubated with 1.5 M ammonium sulfate for 2 h at 4 °C and the supernatant was collected by centrifugation at 10,000 g, then incubated for 2 h with 3 mL pre-equilibrated HIC (hydrophobic interaction chromatography) beads equilibrated with equilibration buffer (10 mM Tris, pH = 7.0, 1 mM EDTA, 10 mM DTT and 1.5 ammonium sulfate) at 4 °C. The beads were isolated and washed with equilibrium buffer (10 mM Tris, pH = 7.0, 1 mM EDTA, 10 mM DTT and 1.5 M ammonium sulfate), followed by protein elution with elution buffer (100 mM Tris, pH = 7.2, 5 mM DTT). Protein elution fractions were pooled and concentrated by filtration at 3000 g (Vivaspin 15R, Sartorius). Samples were loaded onto a Superdex 75 16/60 column (ÄKTA™ pure protein purification system, GE) equilibrated with gel filtration buffer (20 mM HEPES, pH = 7.0, 50 mM NaCl, 5 mM DTT). Pure samples

of MDM2 eluted in a single peak and were estimated to be >95% pure as judged by SDS–PAGE analysis.

MicroScale Thermophoresis (MST) Assay

The binding affinities of compounds towards MDM2 were analyzed using MicroScale Thermophoresis technique. For MST experiment, purified MDM2 protein was prepared following Monolith Protein Labeling Kits RED-NHS protocol (Cat# MD-L001) and pre-tested using Monolith™ NT.115 MST Premium Coated Capillaries by Monolith NT.115. The compound stock solution of 100 mM was prepared in DMSO and diluted with PBS-T buffer (PBS, pH = 7.4, 0.05% Tween 20) to reach the final concentration. Then, compound serial dilution was prepared by 16 x 2-fold serial dilutions with a starting concentration of 0.2 mM. Adding 10 µL prepared protein to compound serial dilution from 1 to 16, mixing well and incubate at room temperature for 20 min. Place 16 capillaries to sample tray after withdrawing protein-compound solution. Binding affinity were calculated from MST trace curve using 20% excitation power, medium MST power at 22 °C.

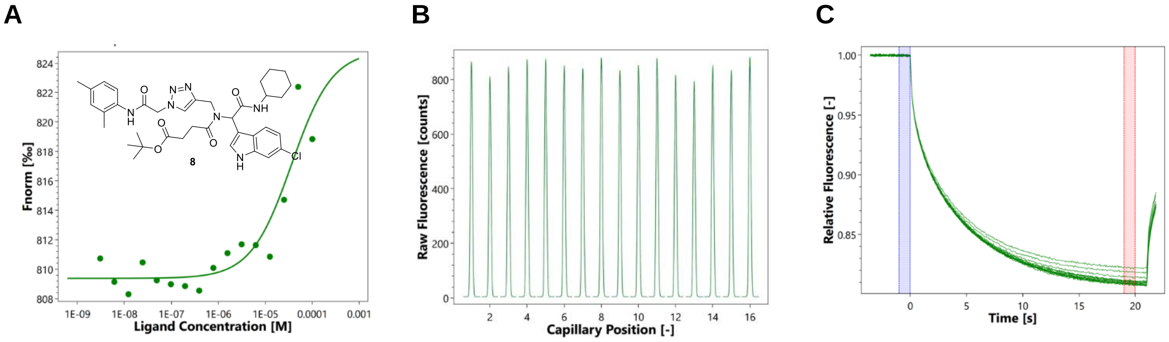

Figure S9: Thermal protein stability curve obtained by MST. (**A**) Dose response curve. K_D = 36.1 µM; signal to noise ratio: 10.1. (**B**) Capillary scan curve. Average: 849 counts; variation: ± 6.2%. (**C**) MST traces. Cold region: -1 s - 0 s; hot region: 19 s - 20 s.

Biological evaluation of compounds 9-15 as hTEAD4 binder

Expression and purification of hTEAD4 [16]

hTEAD4 was expressed, purified and deacylated as reported elsewhere.[16] Briefly, N-terminal His_6-tagged $hTEAD4^{217-434}$ was expressed in *E. coli* BL21-CodonPlus (DE3)-RIPL (Agilent, 230280) from pOPIN-neo vector. The transformed cells were grown at 37 °C in TB medium (0.01% alpha-lactose monohydrate, 2 mM $MgSO_4$, 100 µg/mL ampicillin and 50 µg/mL chloramphenicol) for 4 h. Then, the temperature was reduced to 25 °C and the culture was incubated for another 20-24 h. Cell lysis was performed by sonication in HEPES buffer (50 mM HEPES, 300 mM NaCl, 20 mM imidazole, 1 mM TCEP, pH = 8.0), followed by ultracentrifugation. The cleared lysate was first purified by affinity chromatography using the ÄktaXpress (GE Healthcare, column: His trap FF, GE Healthcare), followed by size exclusion chromatography (SD75 26/60, GE Healthcare) using 20 mM HEPES, 100 mM NaCl, 1 mM TCEP, 2 mM $MgCl_2$, 5% glycerol at pH = 8.0. The purified protein was concentrated using Amicon Ultra Centrifugal Filters, 10 K (Merck), aliquots were snap frozen and stored at -80 °C.

Deacylation (depalmitoylation) of hTEAD4 [16]

To 0.5 mL 2.8 mg/mL recombinant hTEAD4, 30 µL of 105 mM hydroxylamine solution was added and the mixture was gently shaken at room temperature for 3 h. The deacylated hTEAD4 was then purified over two consecutive size exclusion columns: (i) Amicon, 0.5 mL, 10 K (Merck) ii) Zebaspin, 7 K (Thermo Fisher) using Tris (20 mM, pH = 8), NaCl (100 mM), $MgCl_2$ (2 mM) as the final storage buffer.

Nano Differential Scanning Fluorometry (nanoDSF) [16]

To 5 µM of depalmitoylated hTEAD4 were added 10 equiv. of compound in 25 mM HEPES, 200 mM NaCl, 1 mM EDTA, 1 mM TCEP, pH = 7.4. The samples were incubated at room temperature for 60 min, then high sensitivity NanoDSF capillaries (NanoTemper Technologies) were filled with 10 µL of the sample. All the capillaries were placed in the sample holder of the Prometheus NT.48 instrument (NanoTemper Technologies) and a gradient of 1 °C/min from 20 to 85 °C was applied. Melting temperatures were determined with the inflection point of the intrinsic fluorescence curve for the ratio 350/330 nm. All experiments were performed as quadruplicates or triplicates.

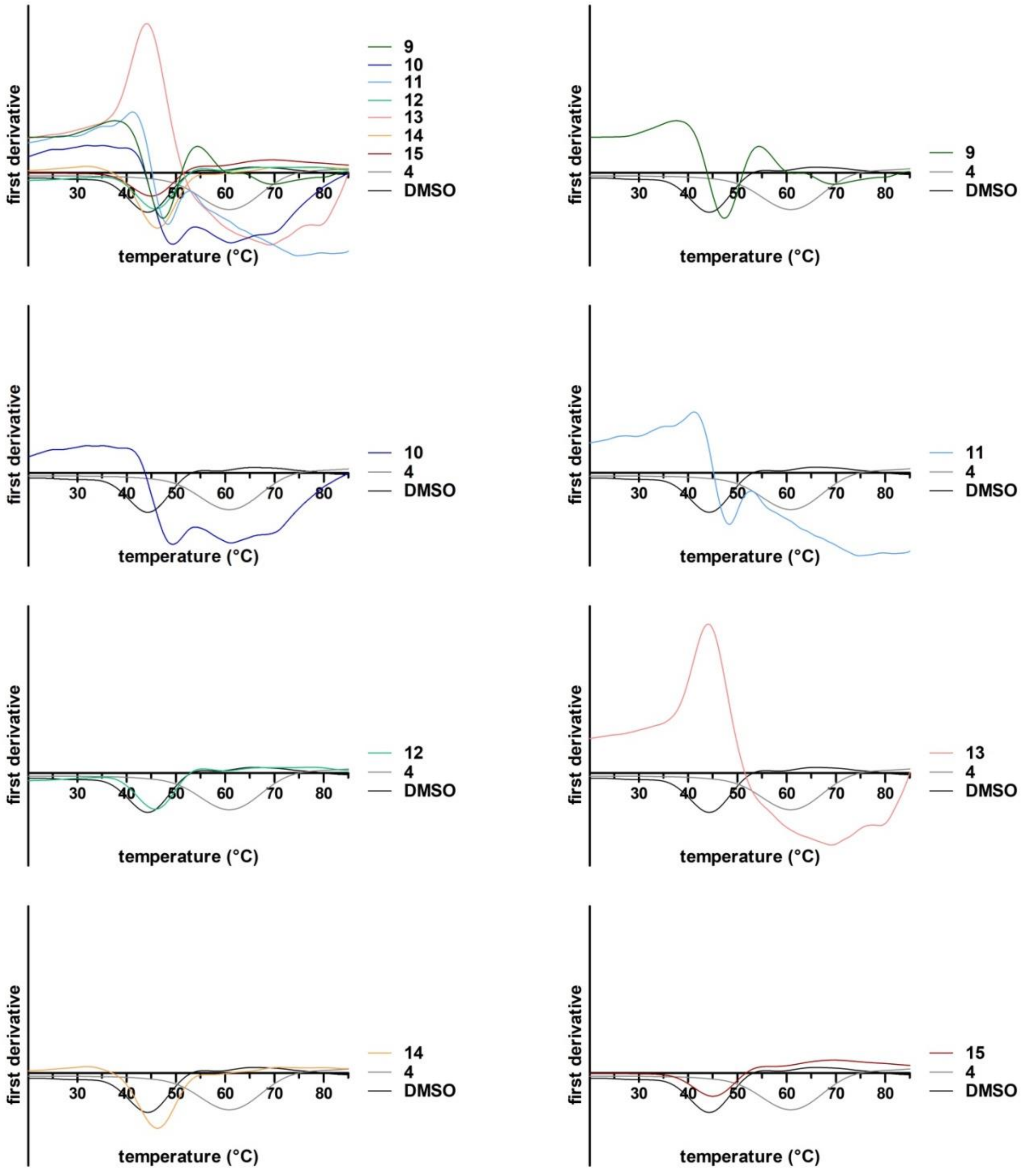

Figure S10: Thermal protein stability curves obtained by nanoDSF. 5 µM of depalmitoylated hTEAD4 was incubated with 10 equiv. of compounds. T_m were determined by mean of the inflection point of the intrinsic fluorescence curve for the ratio 350/330 nm. hTEAD4 T_m in presence of the compounds were compared to the T_m in presence of DMSO only. n: 3-4.

Fluorescence polarization (FP) experiments [16]

FP experiments were established as reported elsewhere.[16] FP experiments were performed using black, low volume, round-bottom, non-binding 384-well plates (Corning 4514). The plates were imaged using Tecan Spark. To increase the solubility of the compounds in buffer solution, we prepared a master solution of compounds containing 7% DMSO and 1% Triton X100.

A

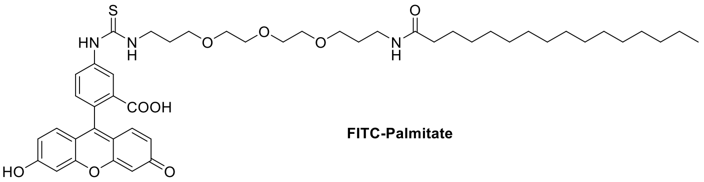

B FITC-AGHQIVHVRGDSETDLEALFNAVMNPKTANVPQTVPMRLRKLPDSFFKPPE

Figure S11: Tracers used in the FP-based competitive binding assays. a) Structure of the FITC-Palmitate tracer. b) Sequence of fluorescently labeled YAP$^{50\text{-}100}$ peptide.

a) Competitive inhibition of hTEAD4 central pocket was performed in HEPES buffer including 150 mM NaCl and 25 mM HEPES at pH = 6.5. Compounds were added to 5 µL buffer followed by addition of 5 µL non-acylated hTEAD4$^{217\text{-}434}$ protein solution and were then incubated for 10 min followed by addition of 5 µL of FITC-labeled palmitate solution. The assay plate was shaken at room temperature for two hours before measuring the fluorescence polarization (Ex./Em: 485/535 nm). Final protein and FITC-labeled palmitate concentrations are 50 nM and 10 nM, respectively. IC_{50} values of the compounds were calculated using GraphPad Prism 7 software.

b) Competitive inhibition of TEAD4-YAP protein-protein interaction was performed in PBS buffer at pH = 7.5. Compounds were added to 5 µL buffer followed by addition of hTEAD4$^{217\text{-}434}$ protein solution. The protein-compound mixture was incubated for 10 min at room temperature followed by addition of 5 µL of FITC-labeled YAP$^{50\text{-}100}$ solution. The assay plate was incubated at 4 °C for another 24 h before measuring the fluorescence polarization (Ex./Em: 485/535 nm). Final protein and FITC-YAP$^{50\text{-}100}$ concentrations are 5 nM and 2 nM, respectively. IC_{50} values of the compounds were calculated using GraphPad Prism 7 software.

Measurement of CTGF expression by RT-qPCR

500.000 HEK293 cells per well were plated in 6-well plate (Greiner BIO-ONE). After 24 h cells were treated with 3 µM XMU-MP-1, combination of XMU-MP-1 (3 µM) and **9** (100 µM and 3 µM) or DMSO as a control for 4 h. Total RNA was extracted using RNeasy Mini Kit (Qiagen) according to manufacturer's instructions. For each sample, 100 ng RNA was transcribed and qPCR was performed using SensiFAST Probe Hi-ROX One-Step Kit (Bioline). Experiment was done in triplicates with suitable controls. RT-qPCR was performed on StepOnePlus Real-Time PCR System (Applied Biosystems) using Primer-Probe Detection method. Conditions: RT-qPCR was performed at 48 °C for 10 min followed by polymerase activation at 95 °C for 2 min and 30 cycles at 95 °C for 5 s and at 60 °C for 20 s. Relative quantification of CTGF mRNA was normalized to housekeeping gene ACTB and data analyzed using Relative Quantification Software (Applied Biosystems). Statistical analysis was done with unpaired two-tailed Student t-test ($p<0.05$, GraphPad Prism 6). Final primer and probe concentrations were 500 nM and 250 nM.

**Primers and probes (Integrated DNA Technologies)**
**CTGF** (assay ID HS.PT.58.14485164.g)
5′-GCTCGGTATGTCTTCATGCTG-3′
5′-GAAGCTGACCTGGAAGAGAAC-3′
5′-HEX-CAGCCAGAA-ZEN-AGCTCAAACTTGATAGGC-IBFQ-3′
**ACTB** (assay ID Hs.PT.39a.22214847)
5′-ACAGAGCCTCGCCTTTG-3′
5′-CCTTGCACATGCCGGAG-3′
5′-FAM-TCATCCATG-ZEN-GTGAGCTGGCGG-IFBQ-3′

ZEN, IBFQ as quenchers

Cell culture medium:

DMEM (PAN Biotech, P04-03590), 10% heat inactivated FBS (Capricorn Scientific), 2 mM L-glutamine (PAN Biotech, P04-80100).

Reagents:

DMSO cell culture grade (PanReac AppliChem, A3672)

XMU-MP-1 (MedChemExpress)

0.05% Trypsin / 0.02% EDTA in PBS (PAN Biotech, P10-0231SP)

DPBS (PAN Biotech, P04-36500)

ΔΔCt calculation:

$$\Delta Ct = Ct(CTGF) - Ct(ACTB)$$
$$\Delta\Delta Ct = \Delta Ct(inhibitor) - \Delta Ct(control)$$

$$\Delta\Delta Ct\ SD = \sqrt{(\Delta Ct\ SD\ control)^2 + \Delta Ct\ SD\ inhibitor)^2}$$

ΔΔCt SD was calculated using Gaussian error propagation.

Compound characterization data

MALDI spectra of hexT conjugates

Compounds **hexT 4-81** were prepared by Ugi-reaction according to the representative procedure RP-2.

hexT 4

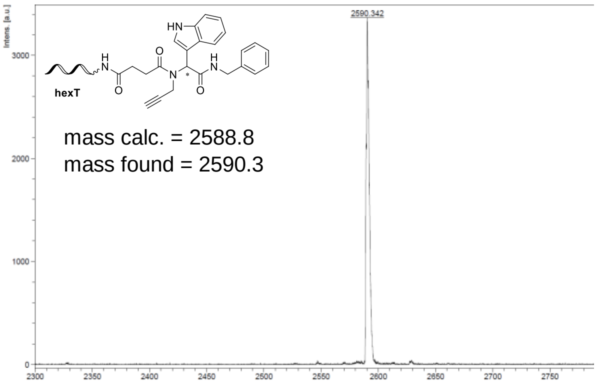

hexT 5

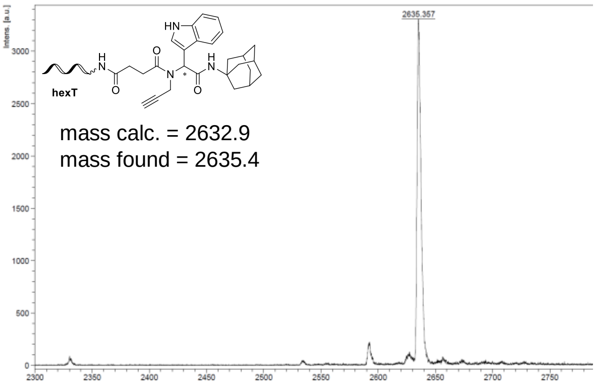

hexT 6

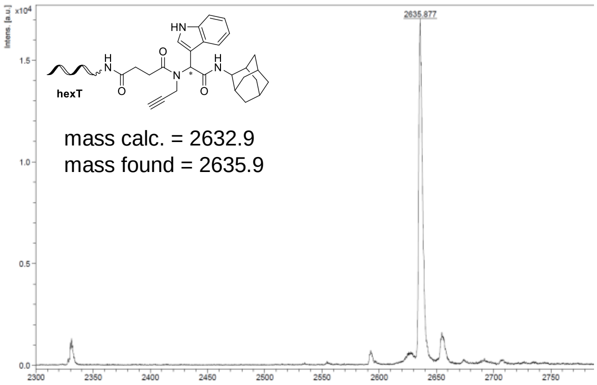

hexT 7

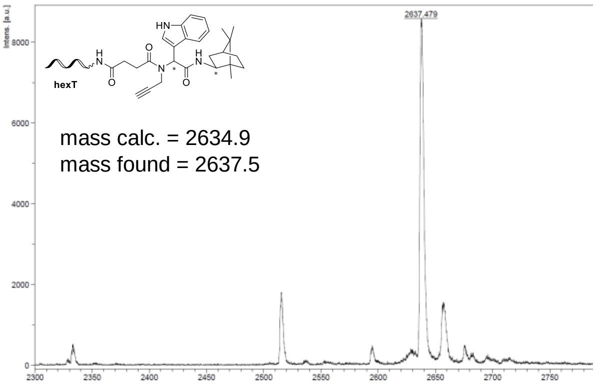

hexT 8

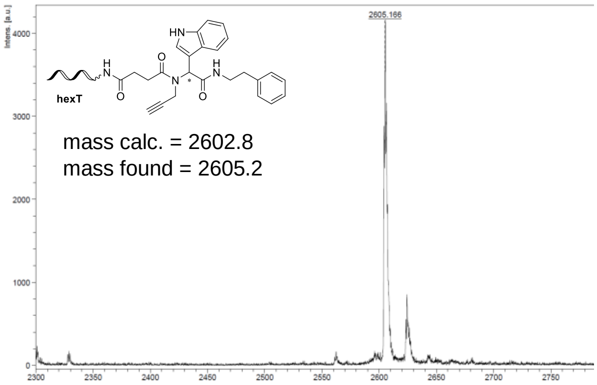

hexT 9

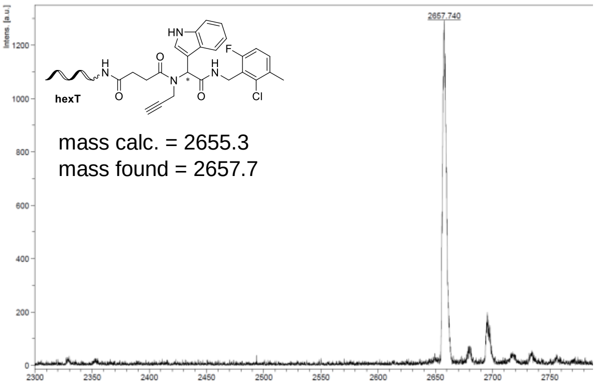

hexT 10

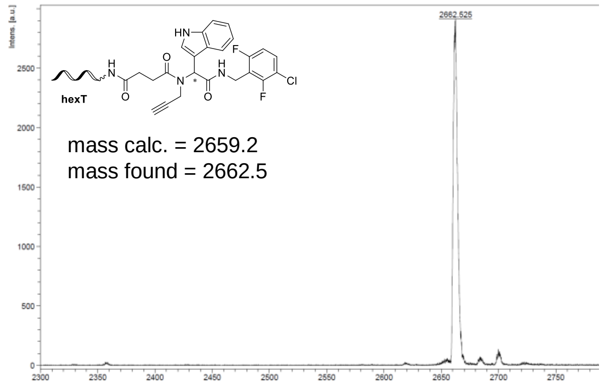

hexT 11

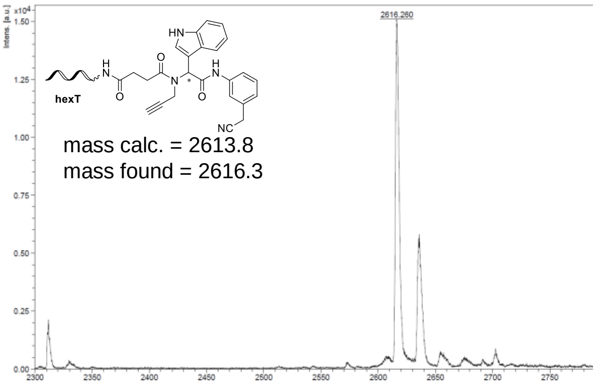

hexT 12

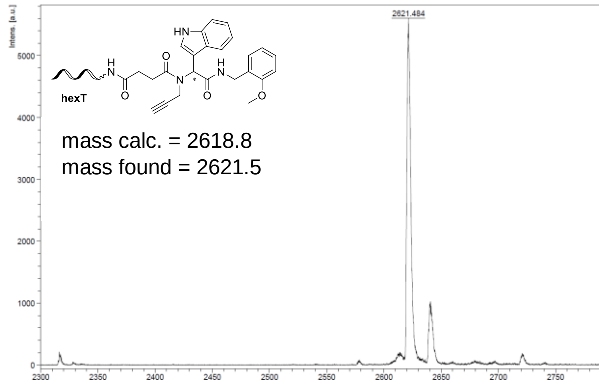

hexT 13

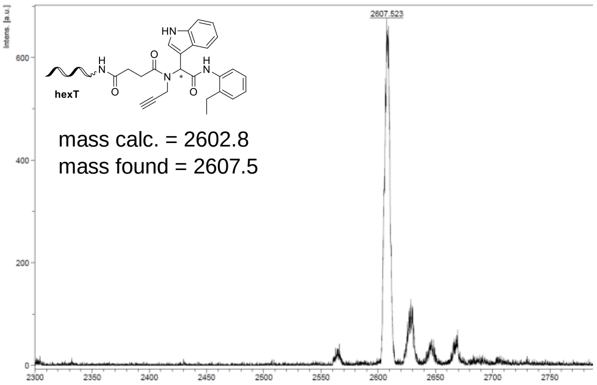

hexT 14

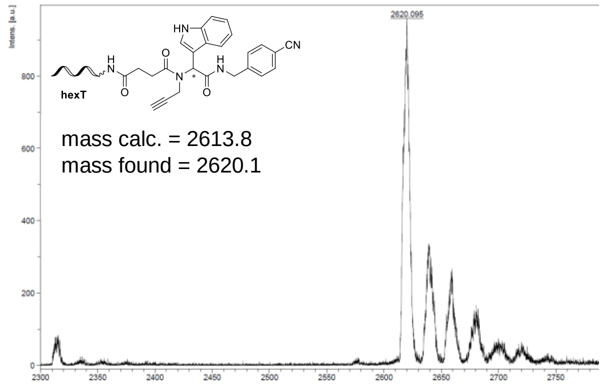

hexT 15

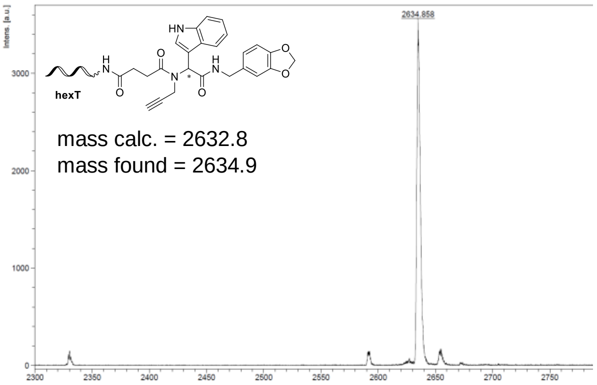

hexT 16

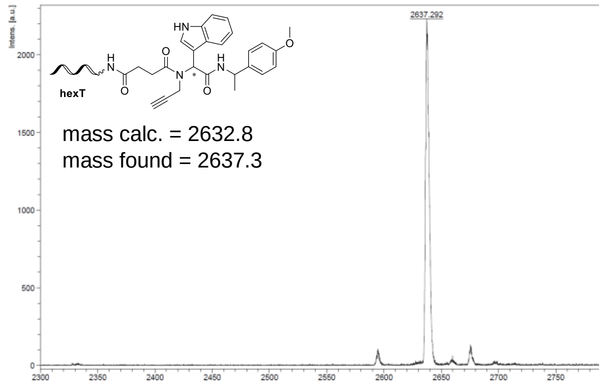

hexT 17

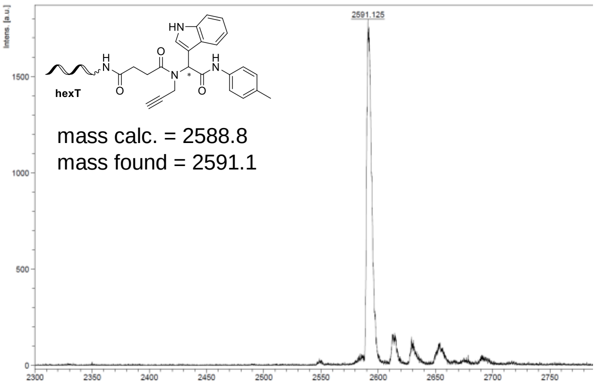

hexT 18

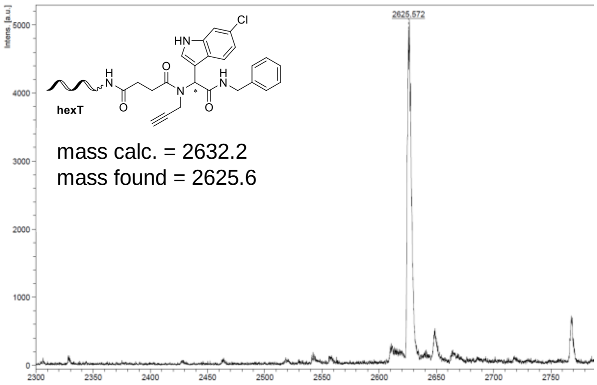

hexT 19

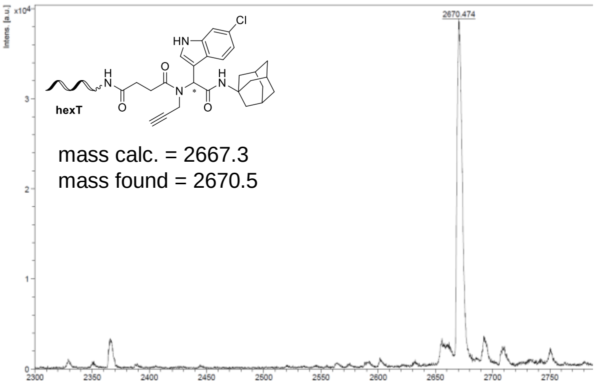

hexT 20

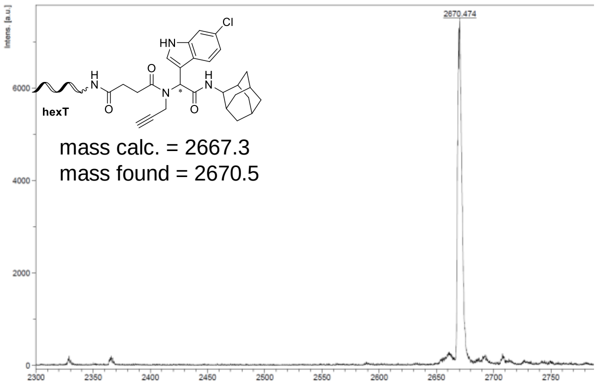

hexT 21

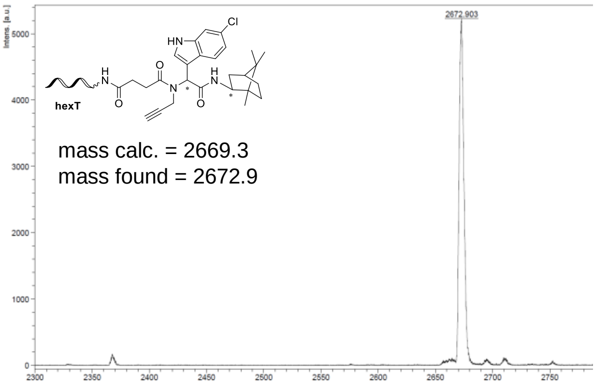

hexT 22

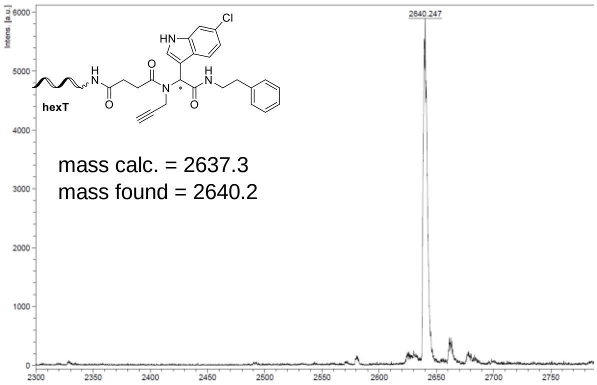

hexT 23

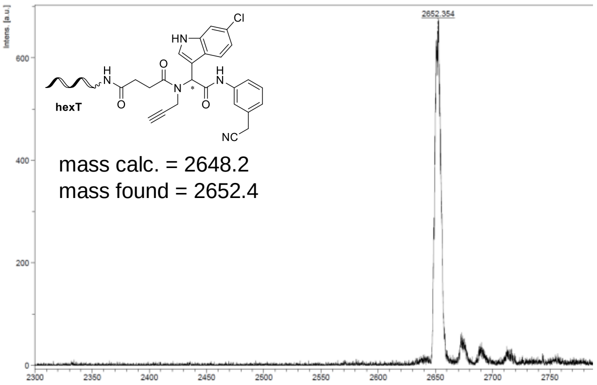

hexT 24

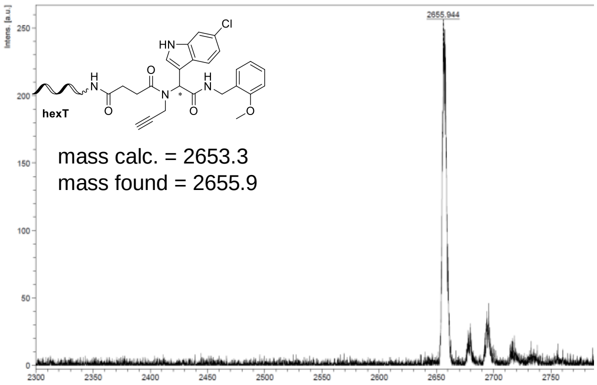

hexT 25

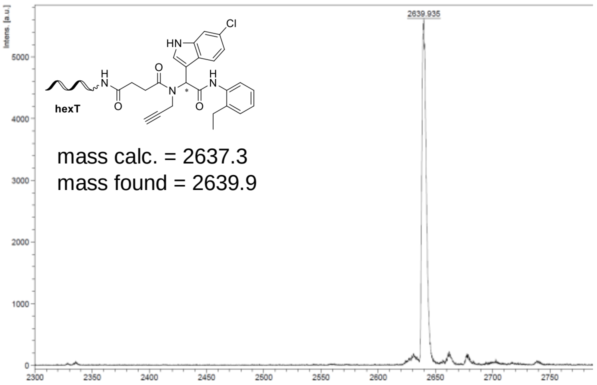

hexT 26

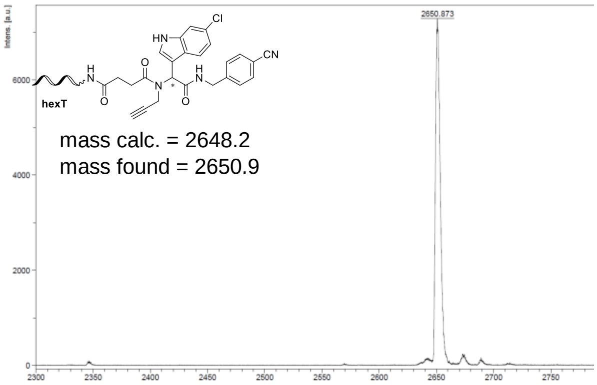

hexT 27

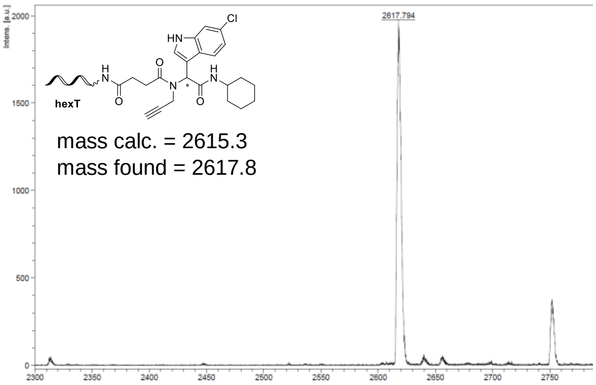

hexT 28

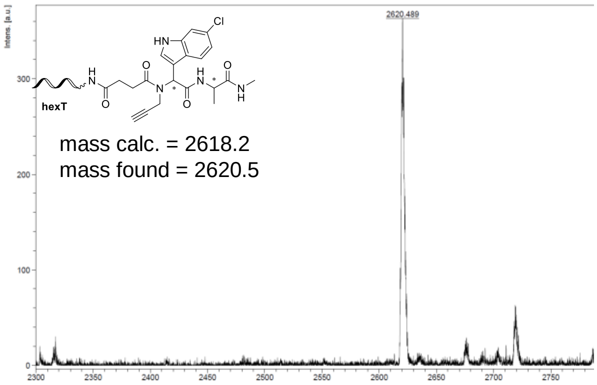

hexT 29

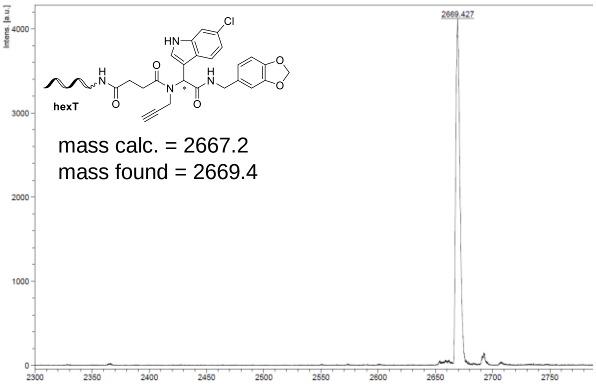

hexT 30

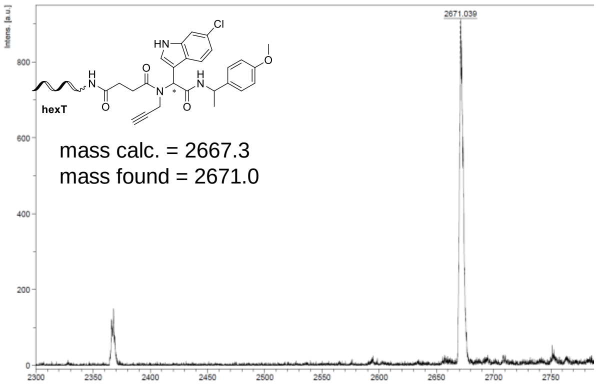

hexT 31

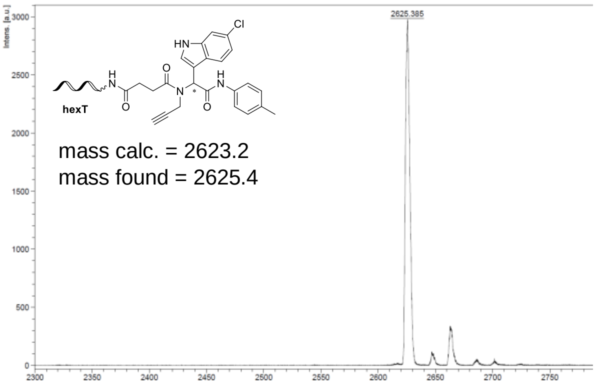

hexT 32

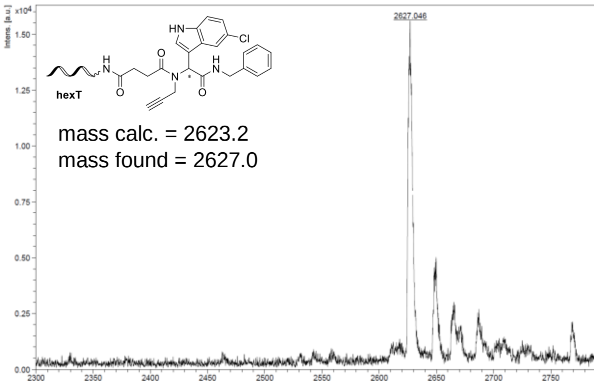

hexT 33

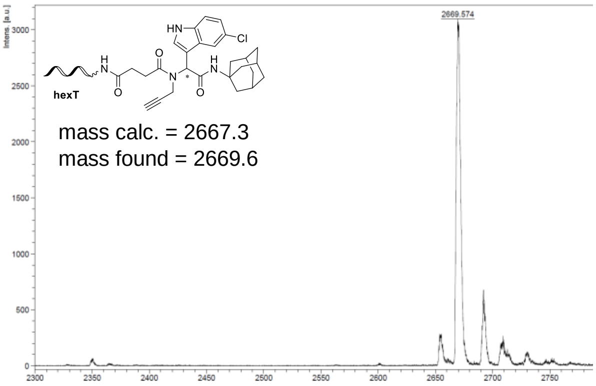

hexT 34

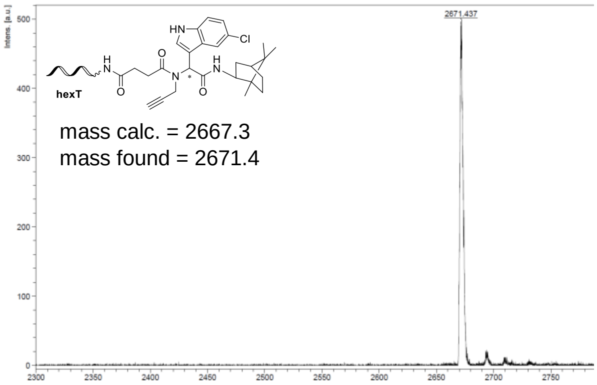

hexT 35

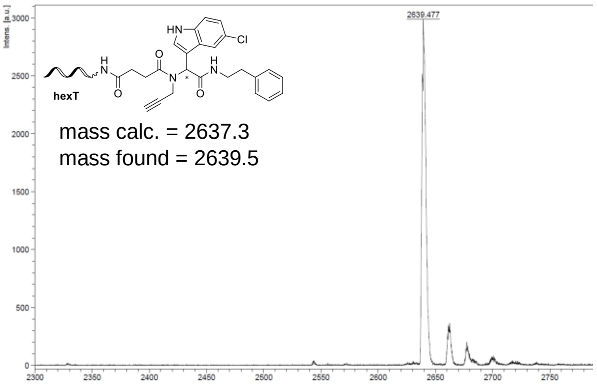

hexT 36

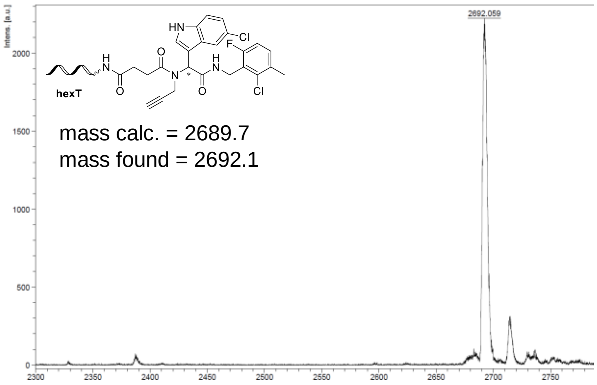

hexT 37

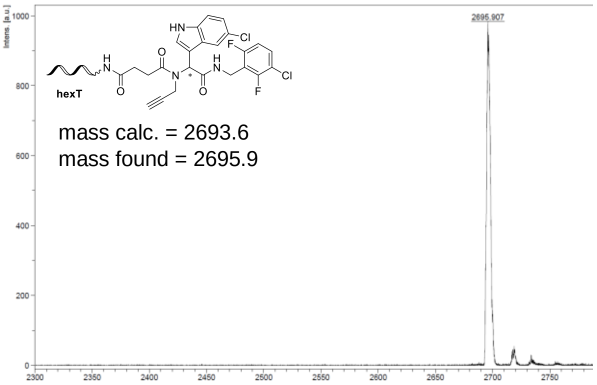

hexT 38

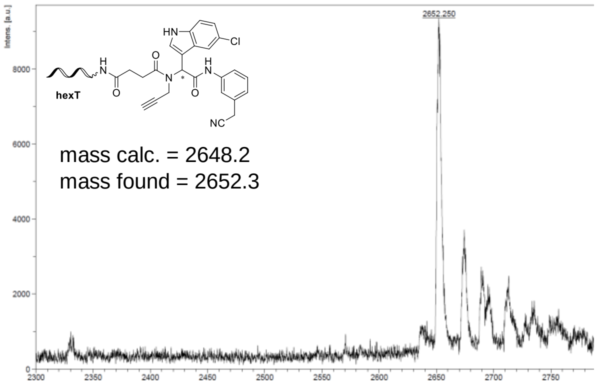

hexT 39

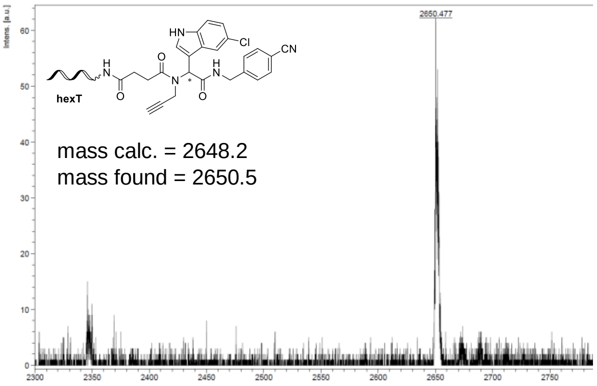

hexT 40

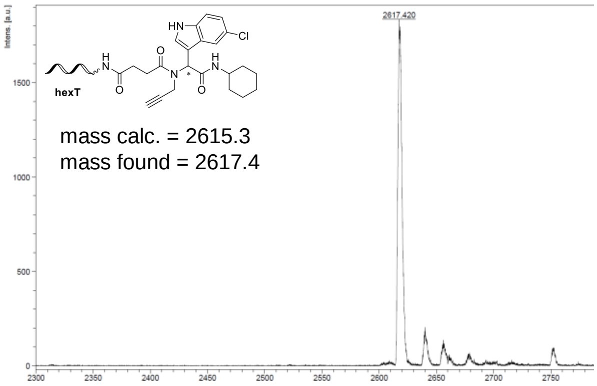

hexT 41

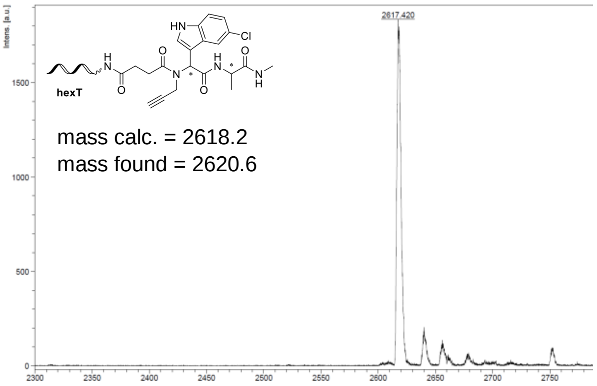

hexT 42

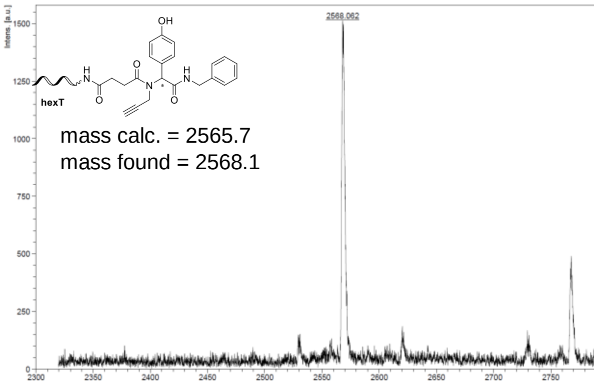

hexT 43

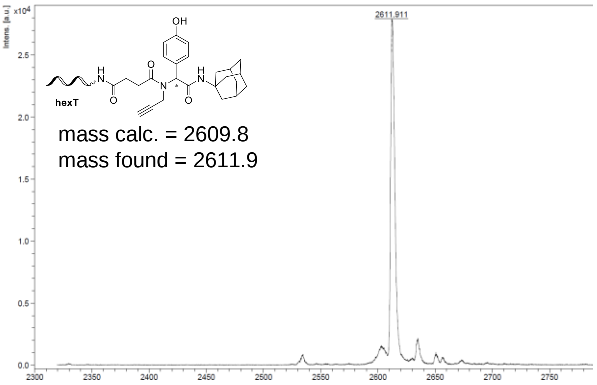

hexT 44

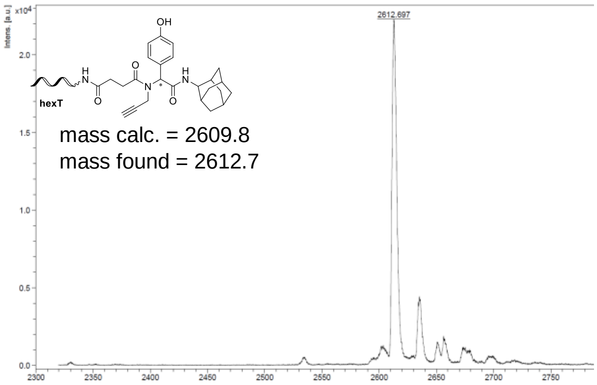

hexT 45

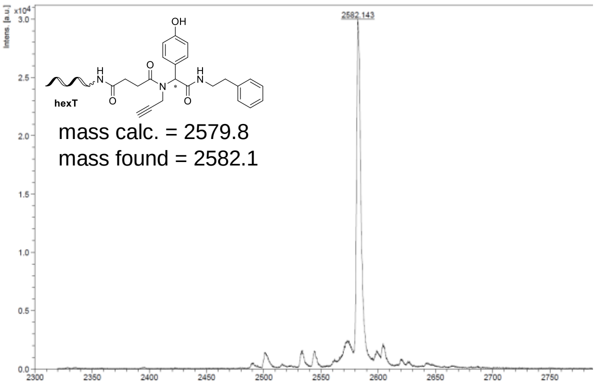

hexT 46

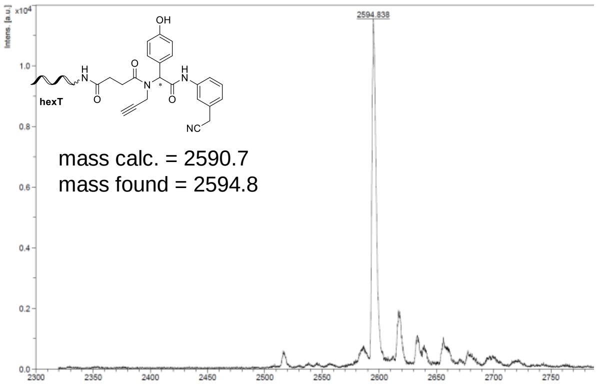

hexT 47

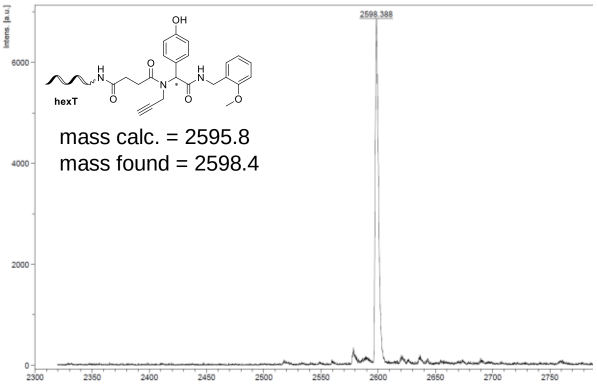

hexT 48

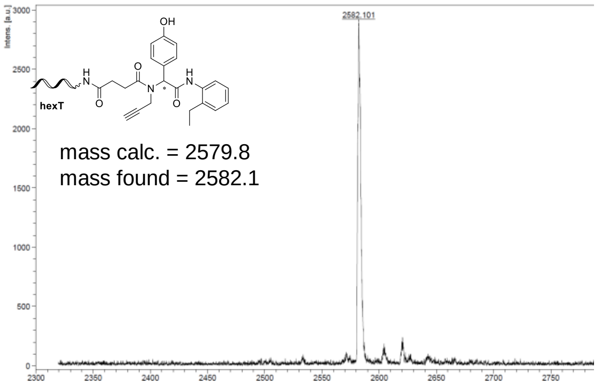

hexT 49

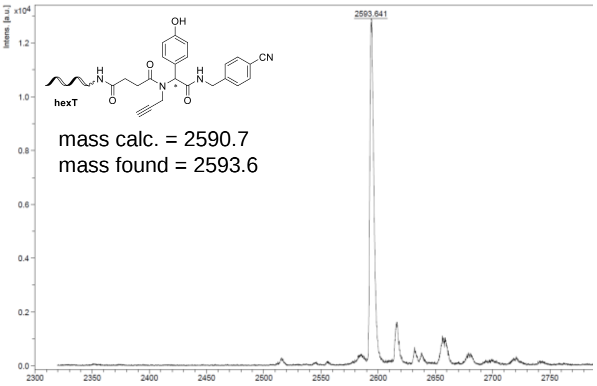

hexT 50

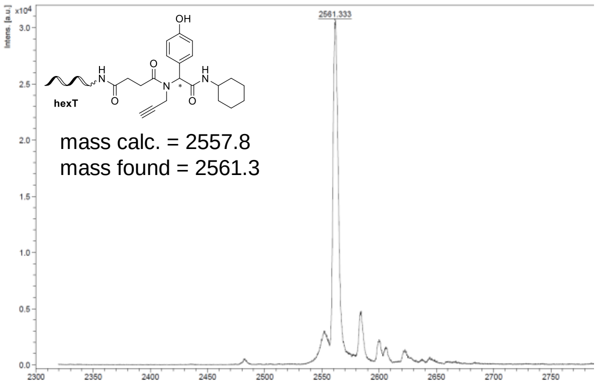

hexT 51

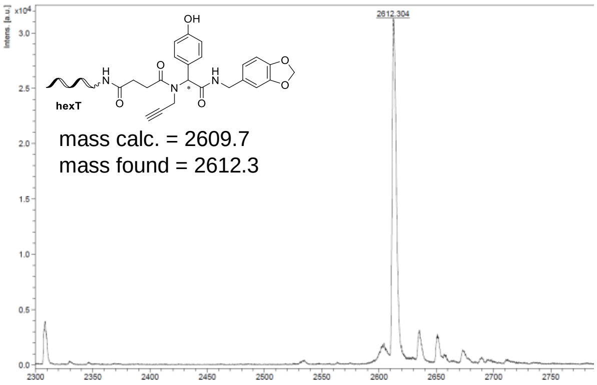

hexT 52

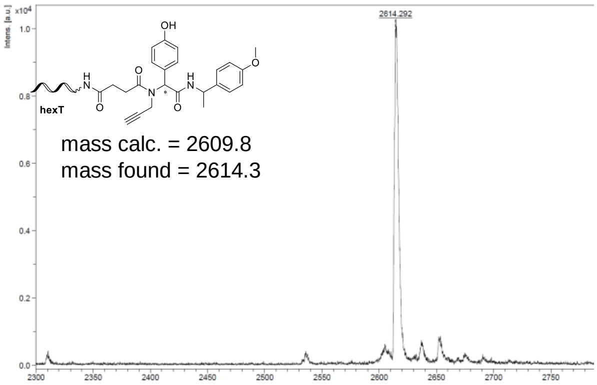

hexT 53

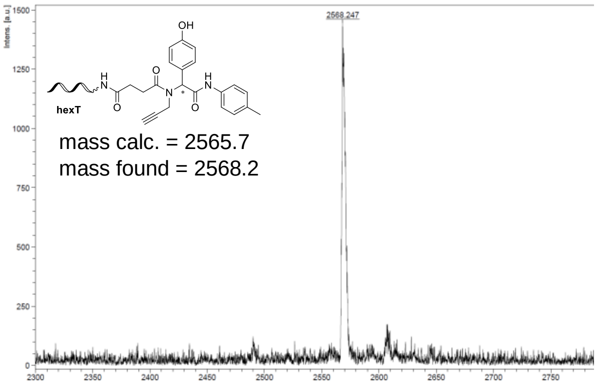

hexT 54

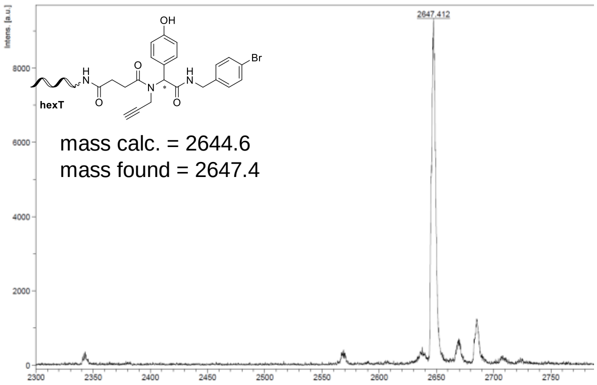

hexT 55

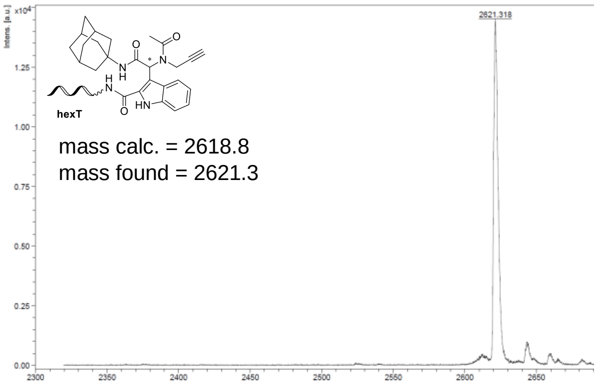

hexT 56

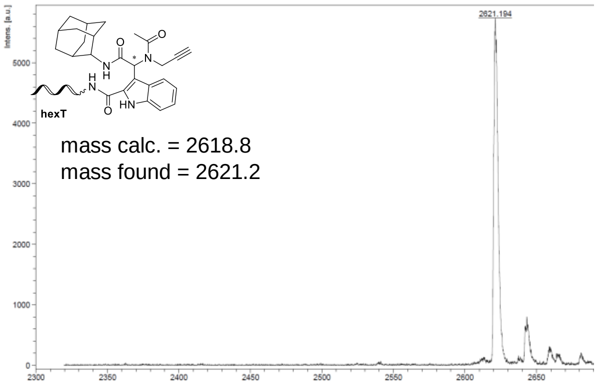

hexT 57

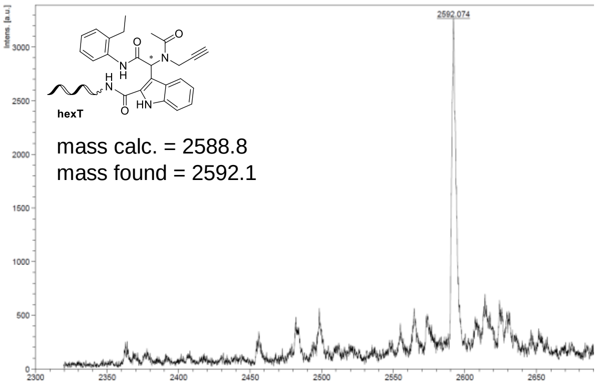

hexT 58

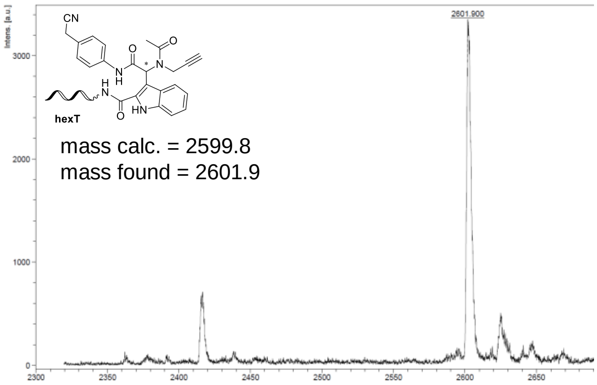

hexT 59

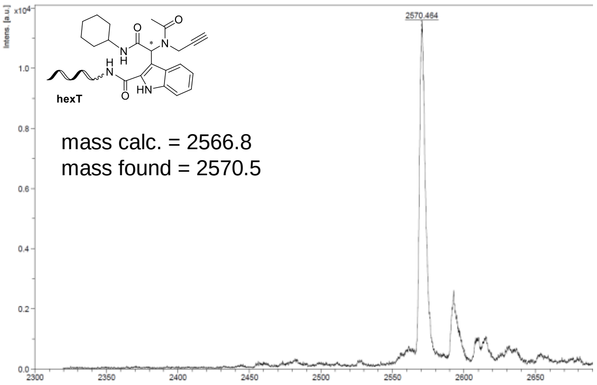

hexT 60

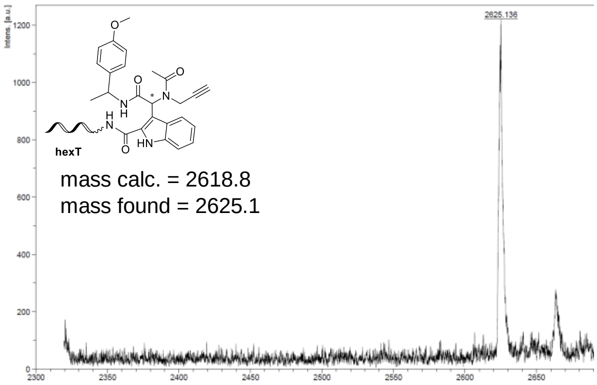

hexT 61

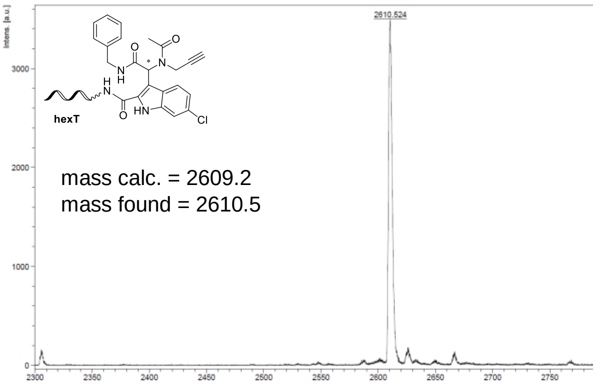

hexT 62

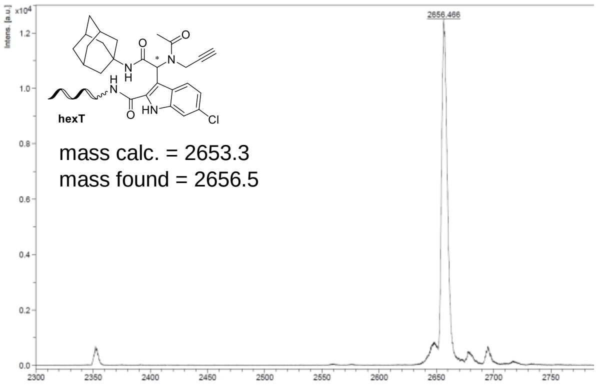

hexT 63

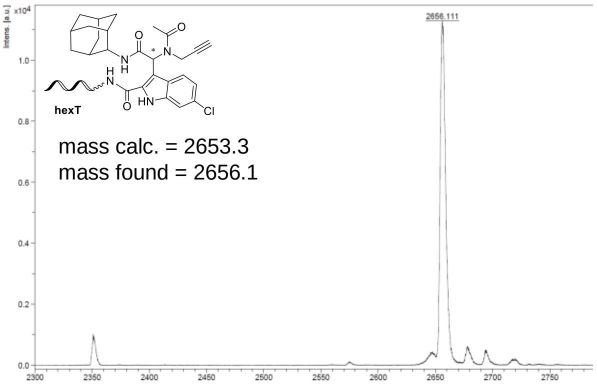

hexT 64

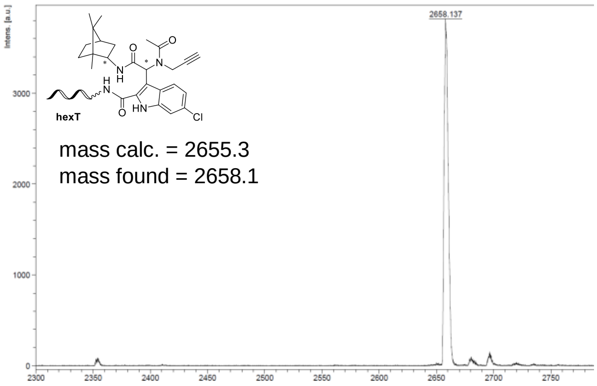

hexT 65

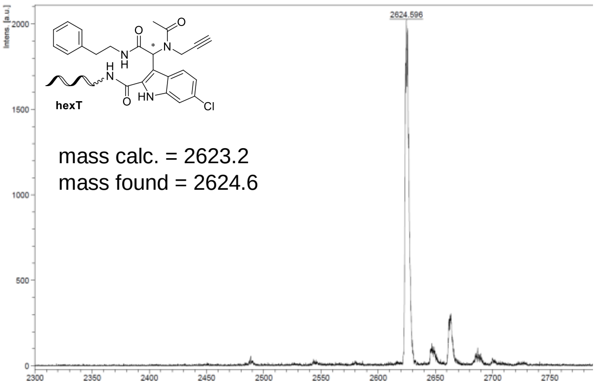

hexT 66

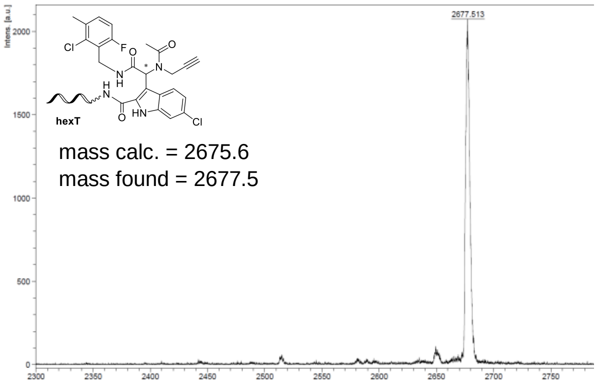

hexT 67

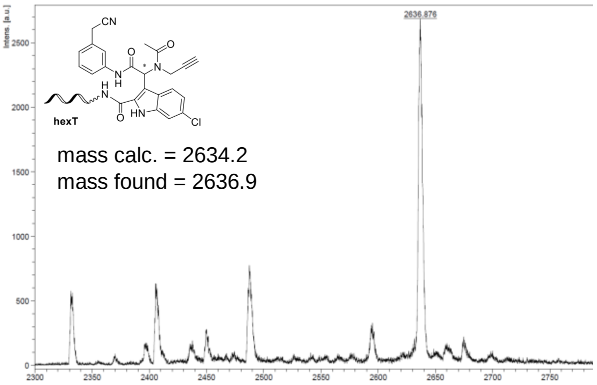

hexT 68

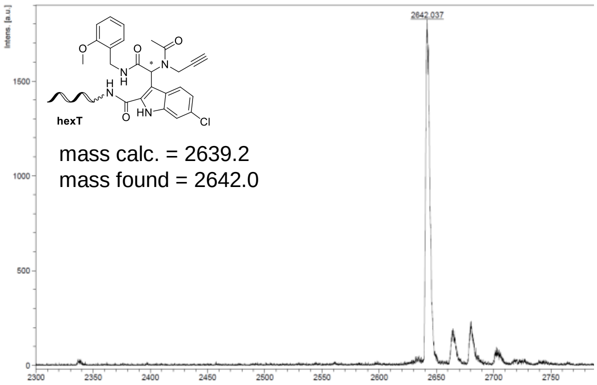

hexT 69

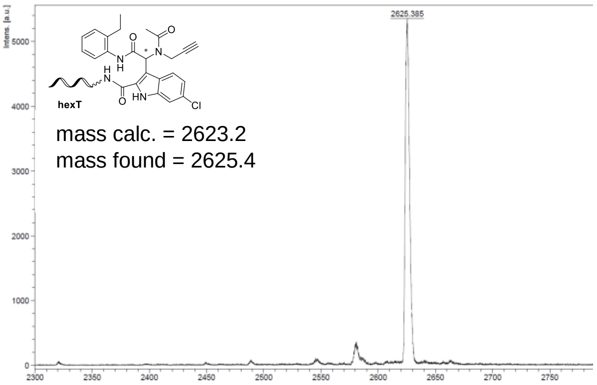

hexT 70

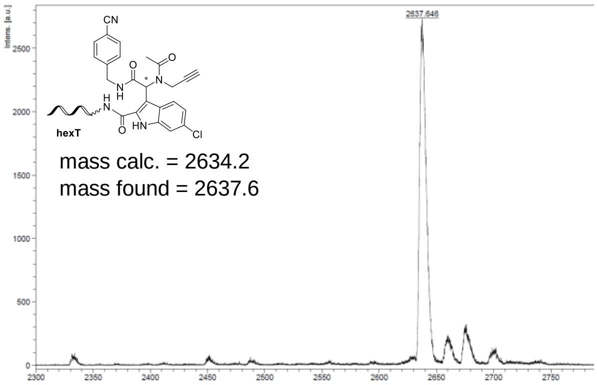

hexT 71

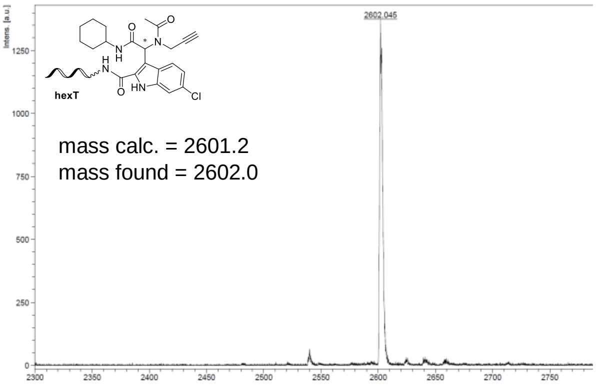

hexT 72

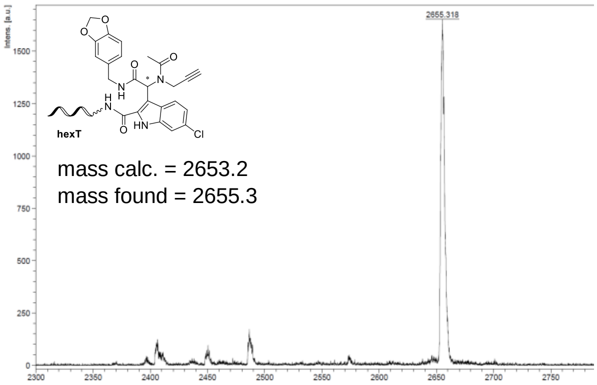

hexT 73

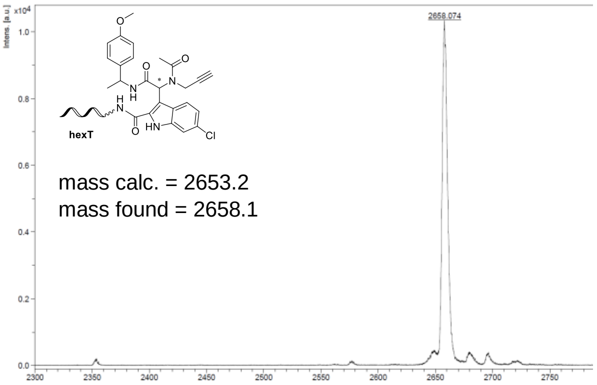

hexT 74

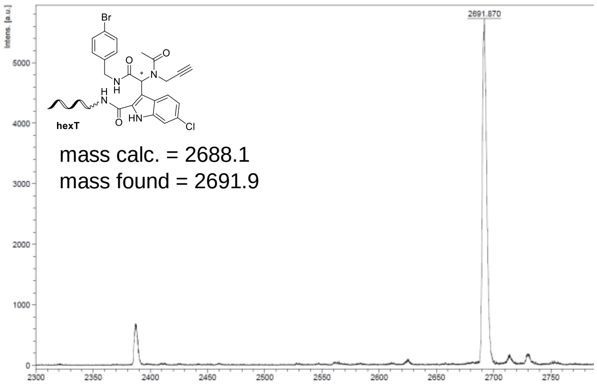

hexT 75

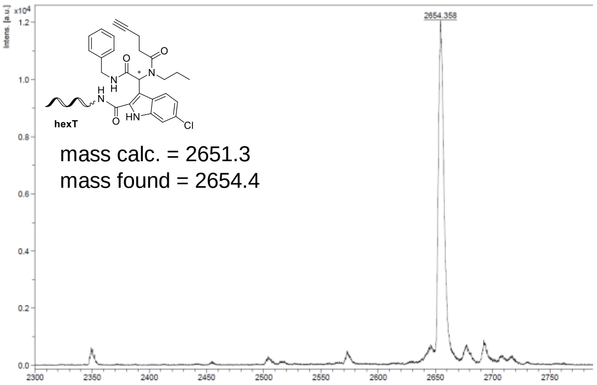

hexT 76

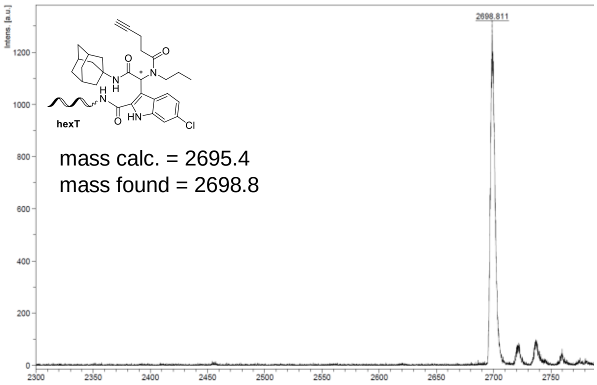

hexT 77

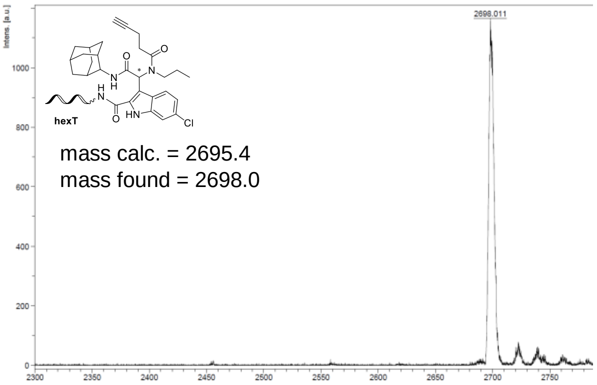

hexT 78

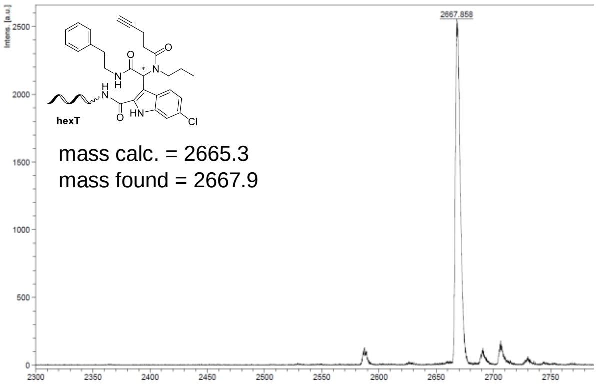

hexT 79

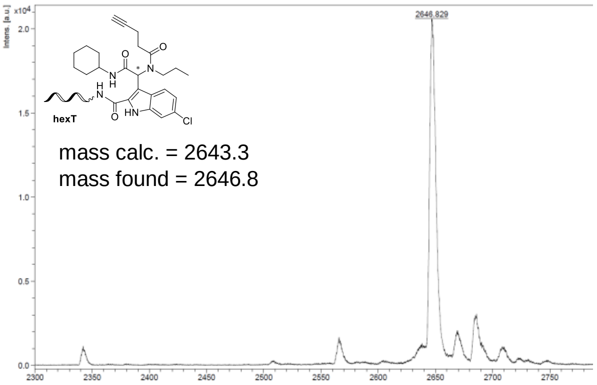

hexT 80

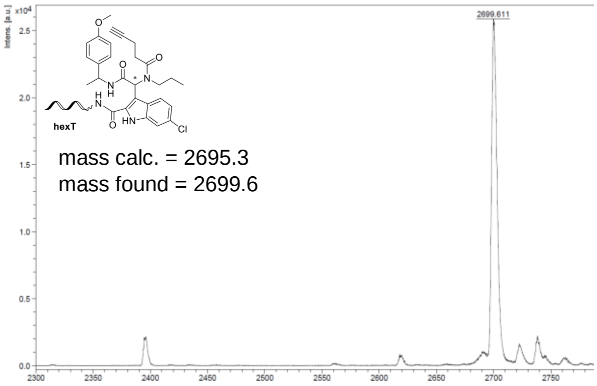

hexT 81

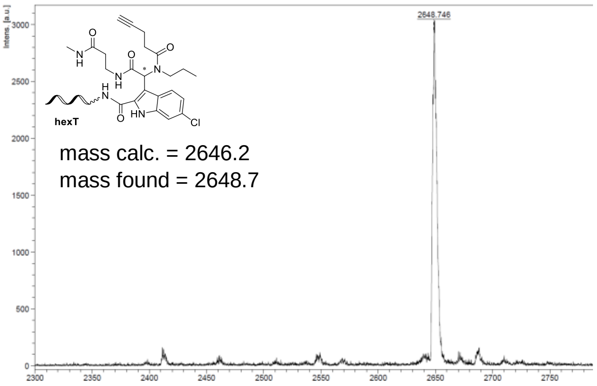

^{1}H and ^{13}C NMR spectra for isocyanides I4, I10, I11, I15

2-isocyano-1,7,7-trimethylbicyclo[2.2.1]heptane (I4)

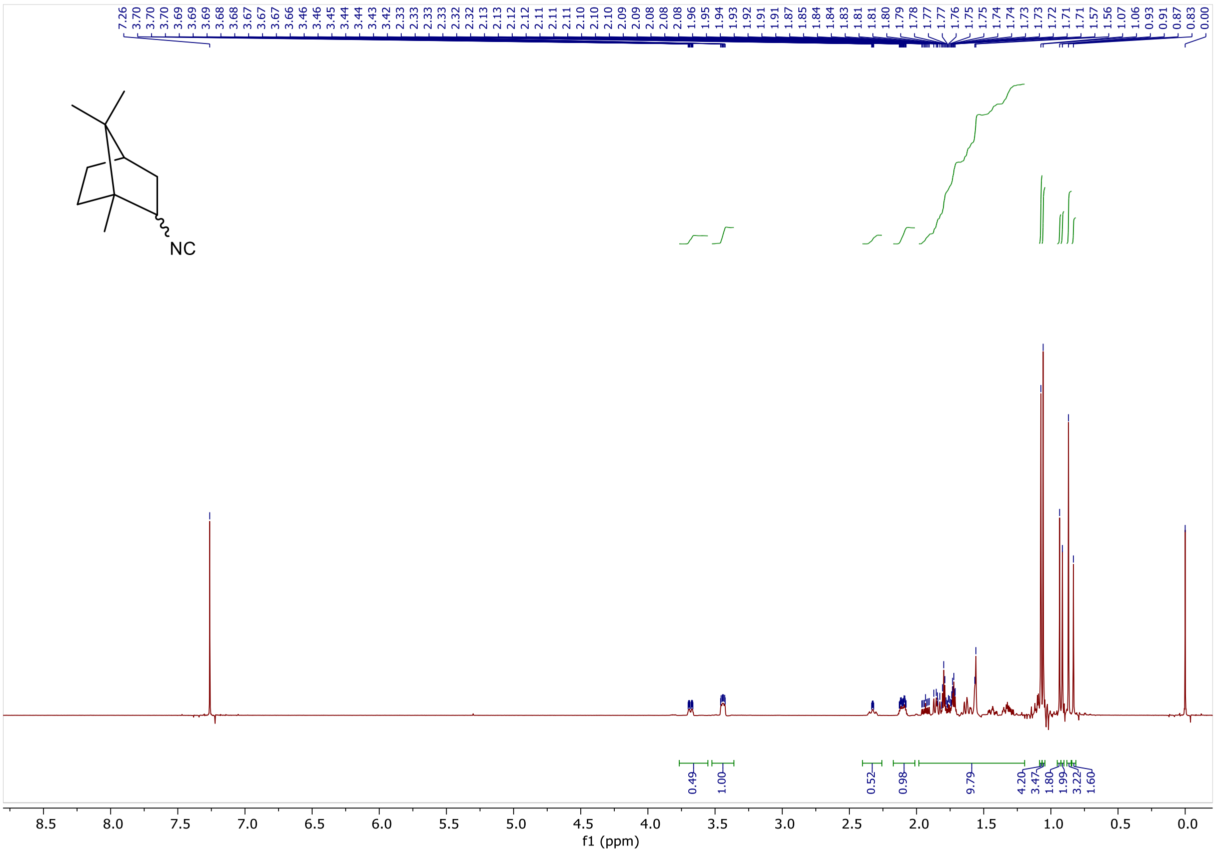

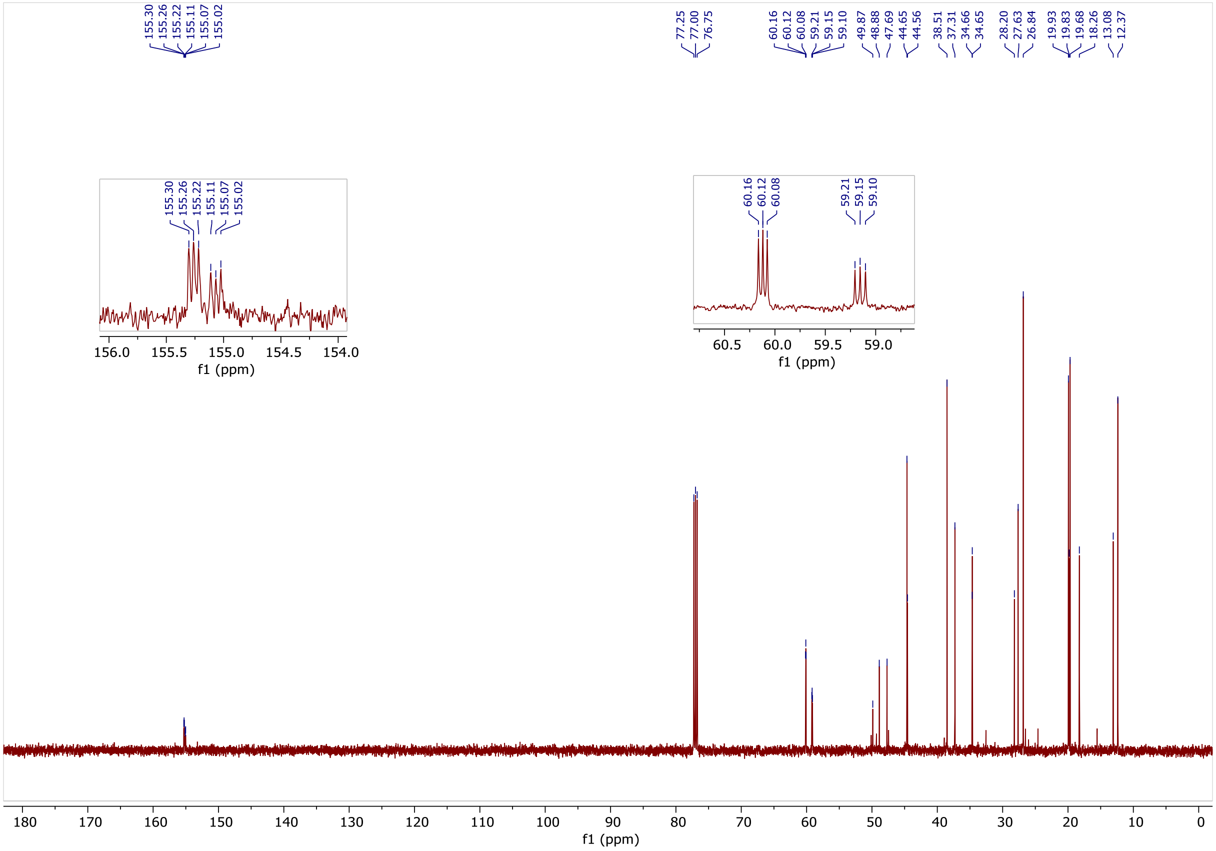

1-ethyl-2-isocyanobenzene (I10)

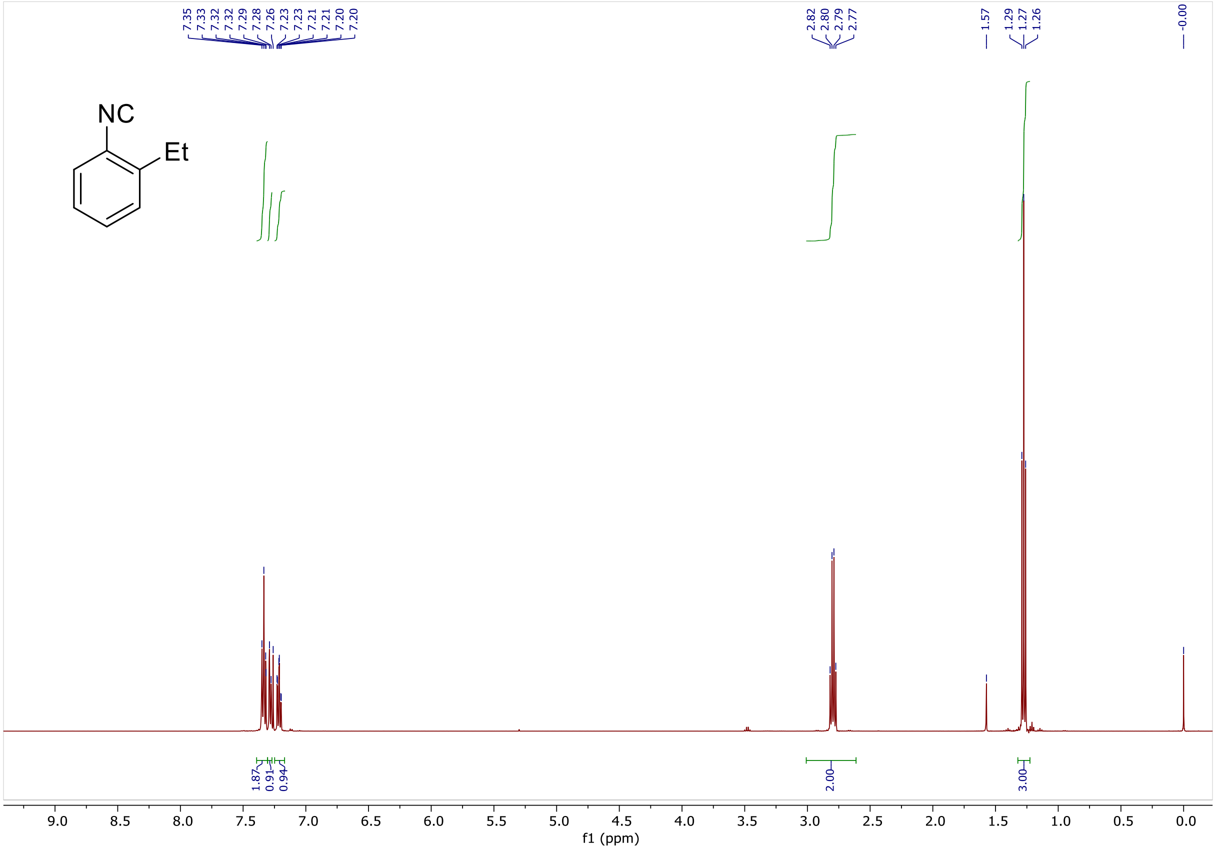

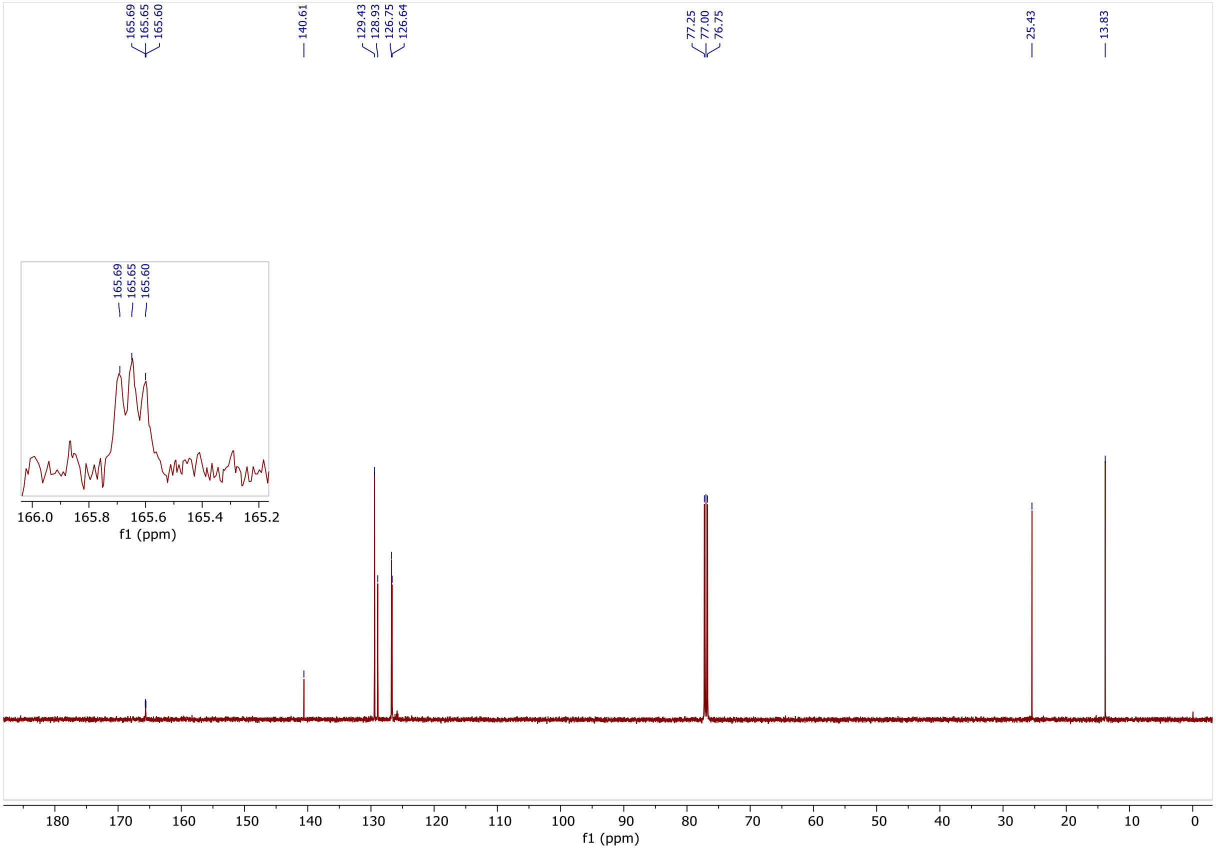

4-(isocyanomethyl)benzonitrile (I11)

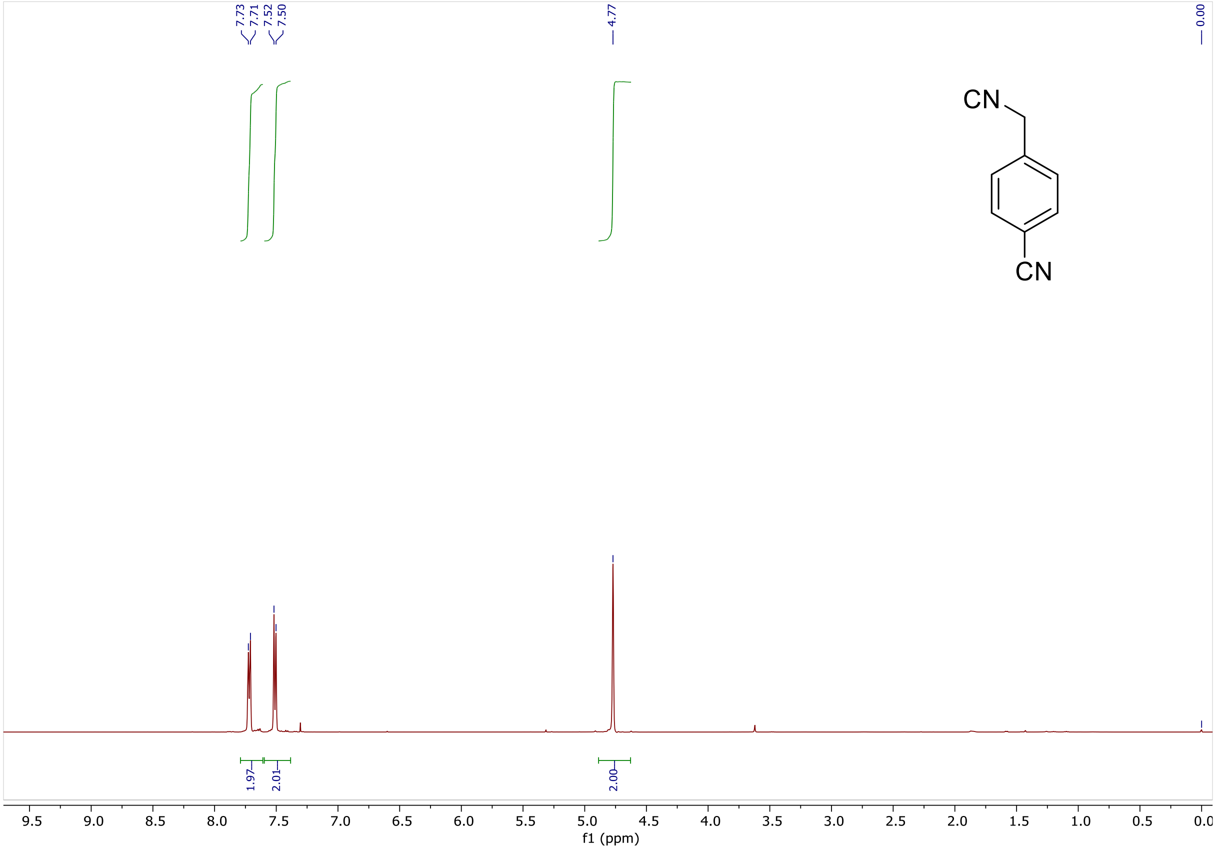

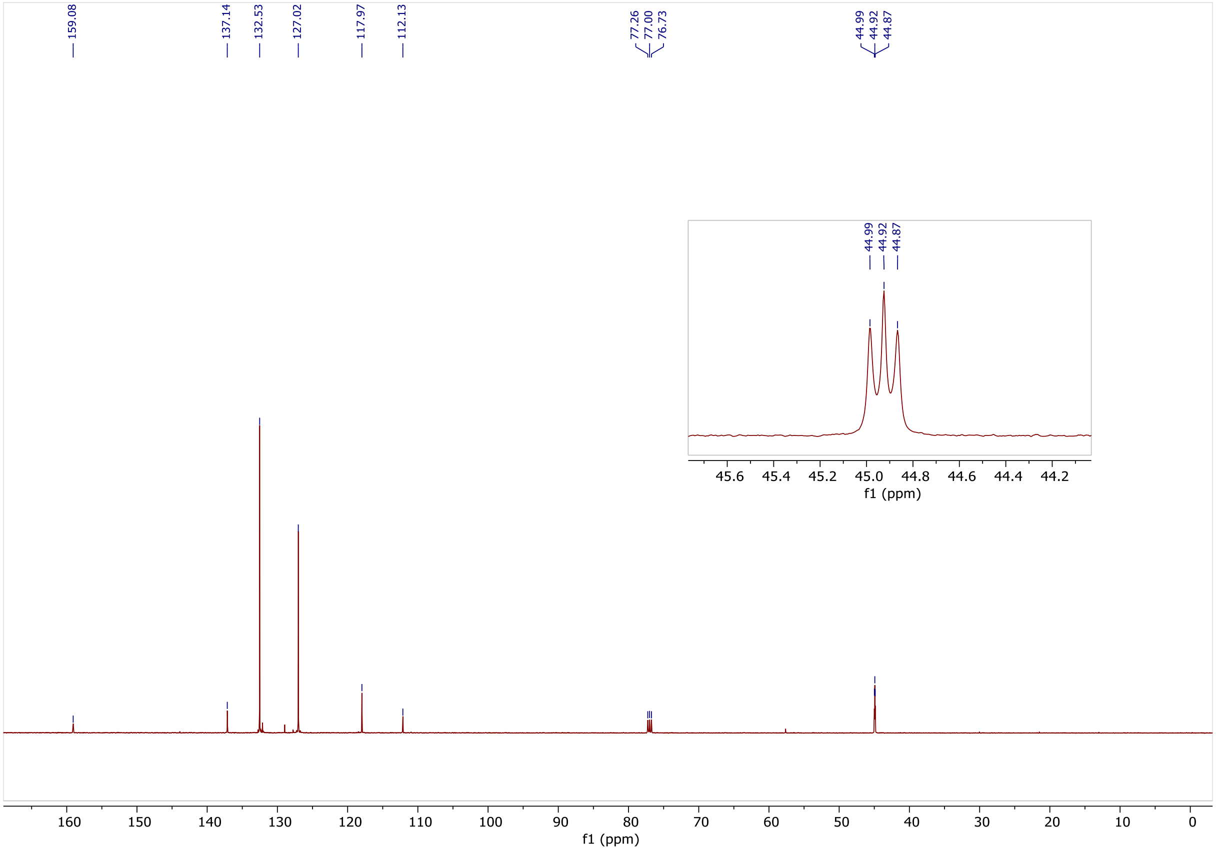

1-(1-isocyanoethyl)-4-methoxybenzene (I15)

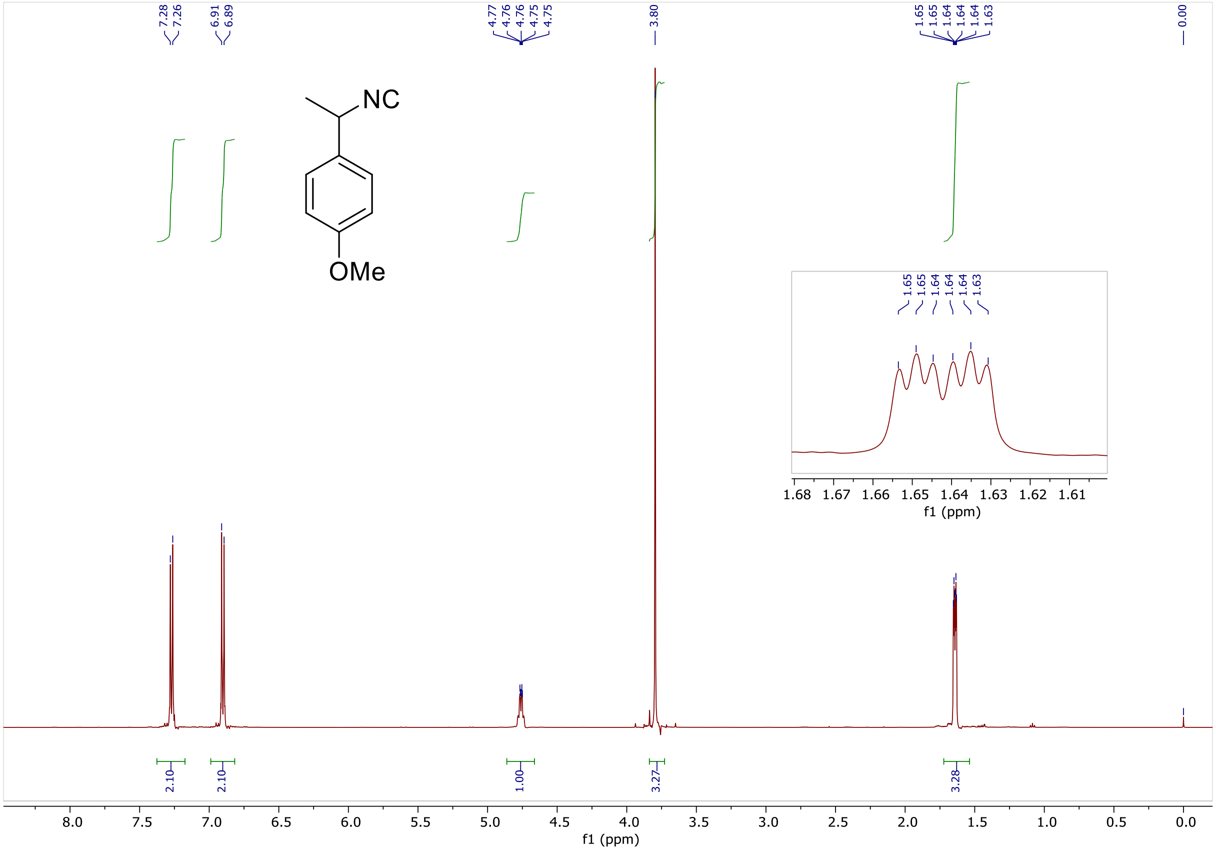

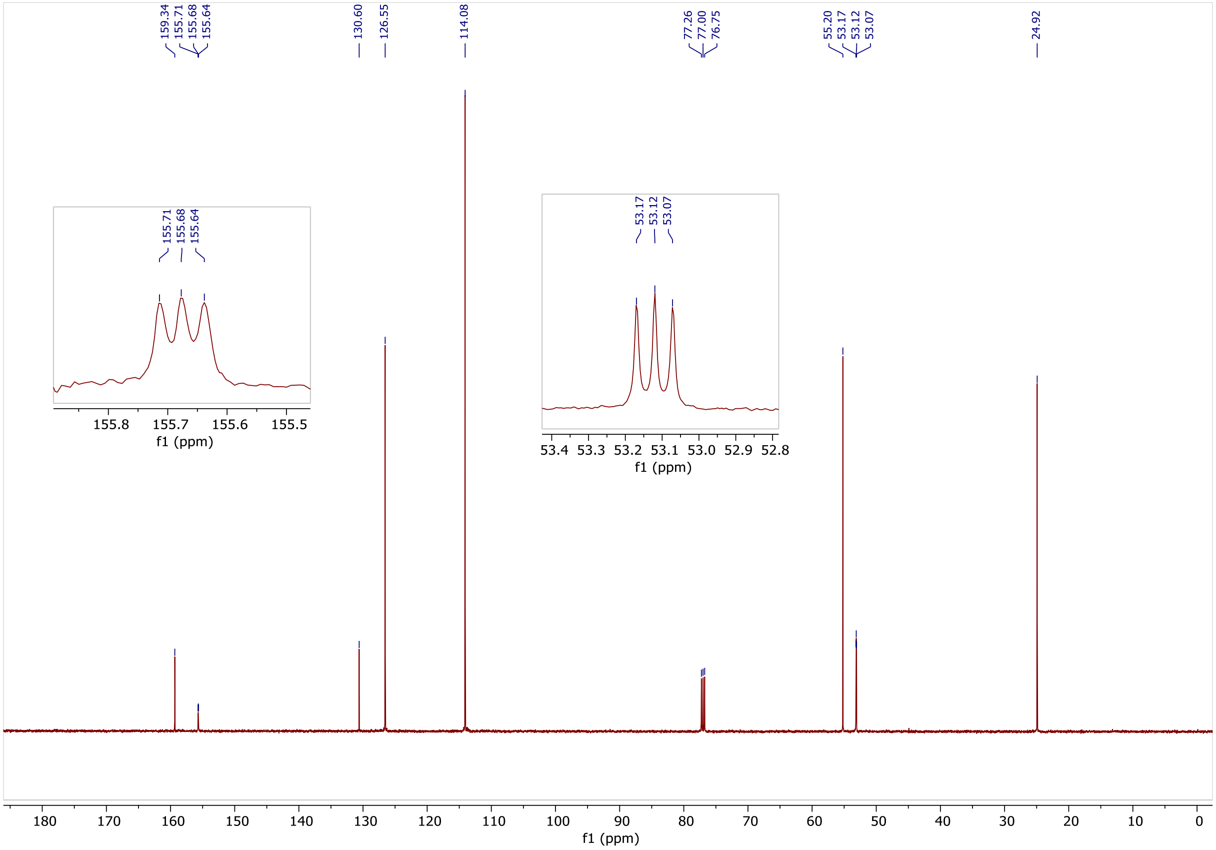

^{1}H and ^{13}C NMR and RP-HPLC-MS spectra for compounds 8-15

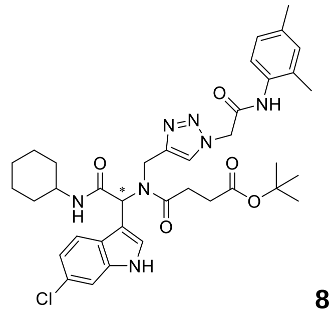

8

^{1}H NMR

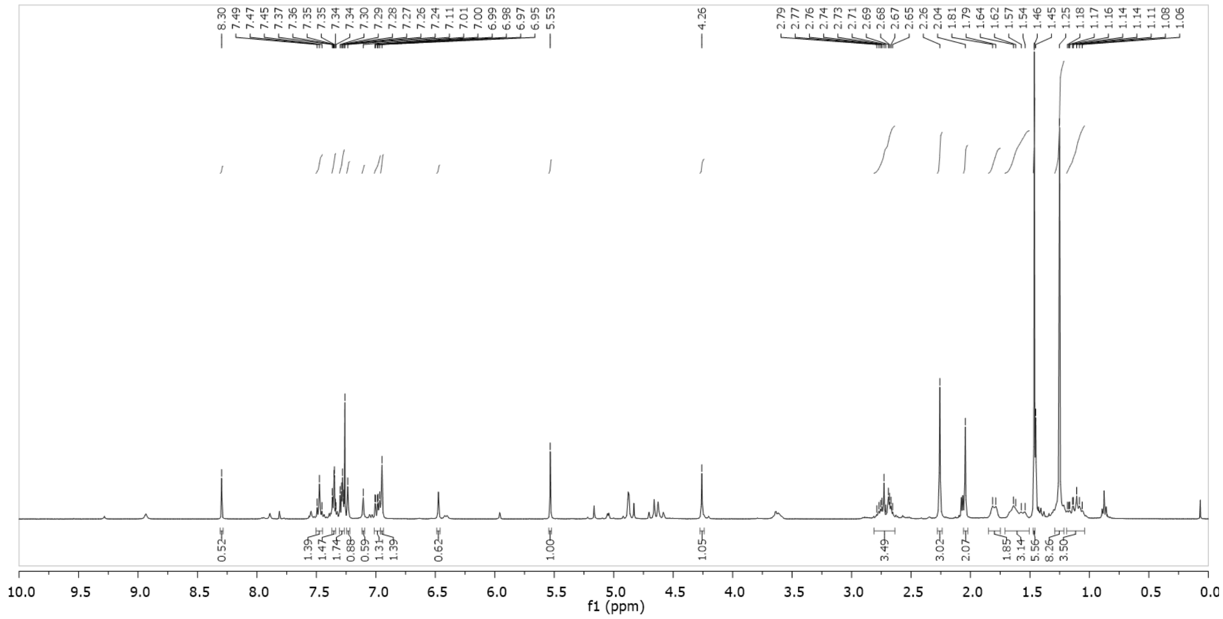

^{13}C NMR

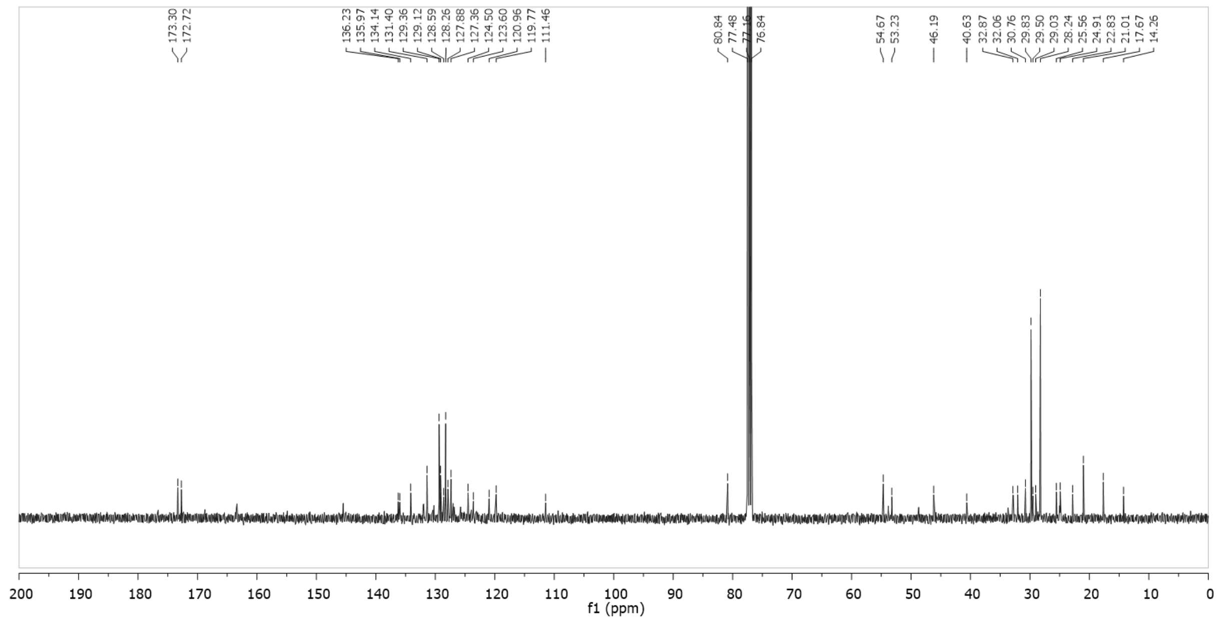

Analytical RP-HPLC-MS (I)

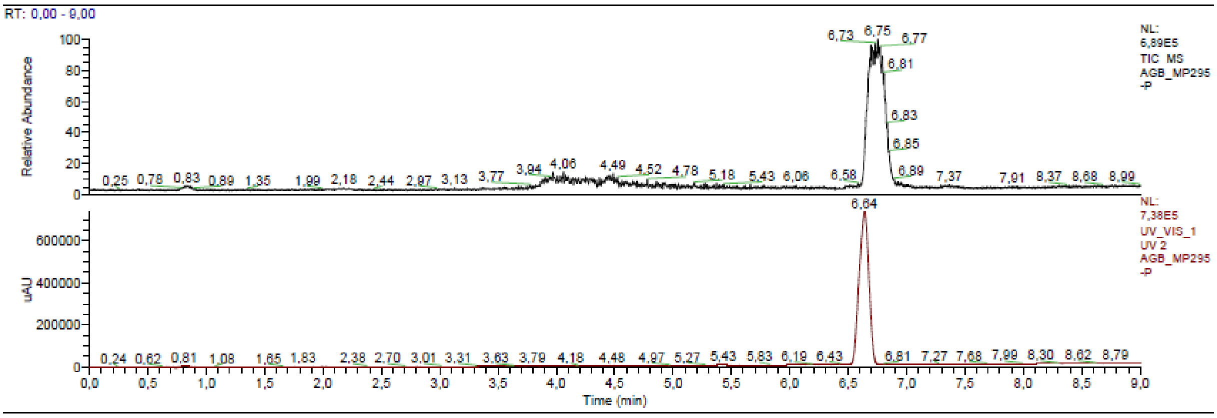

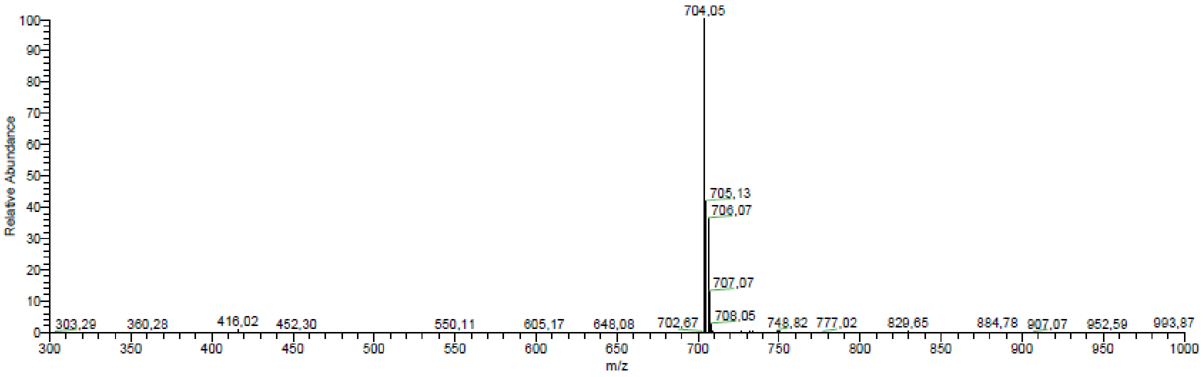

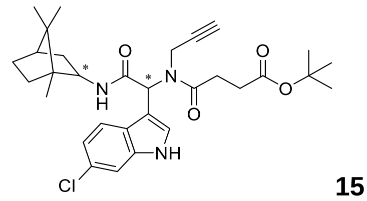

15

¹H NMR

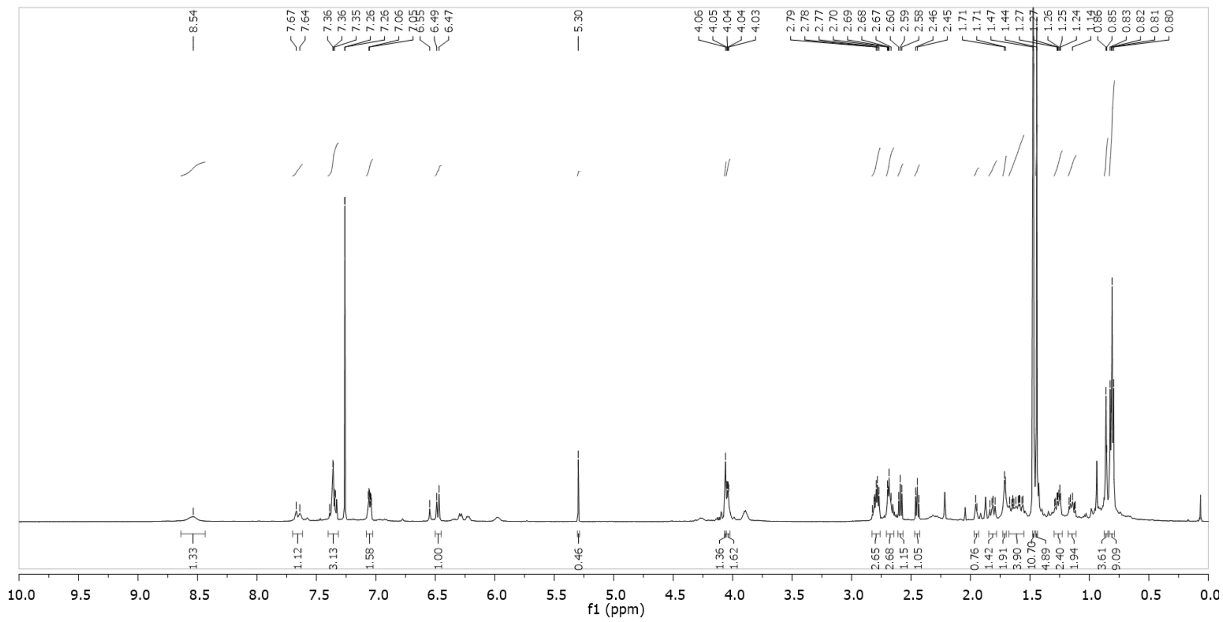

¹³C NMR

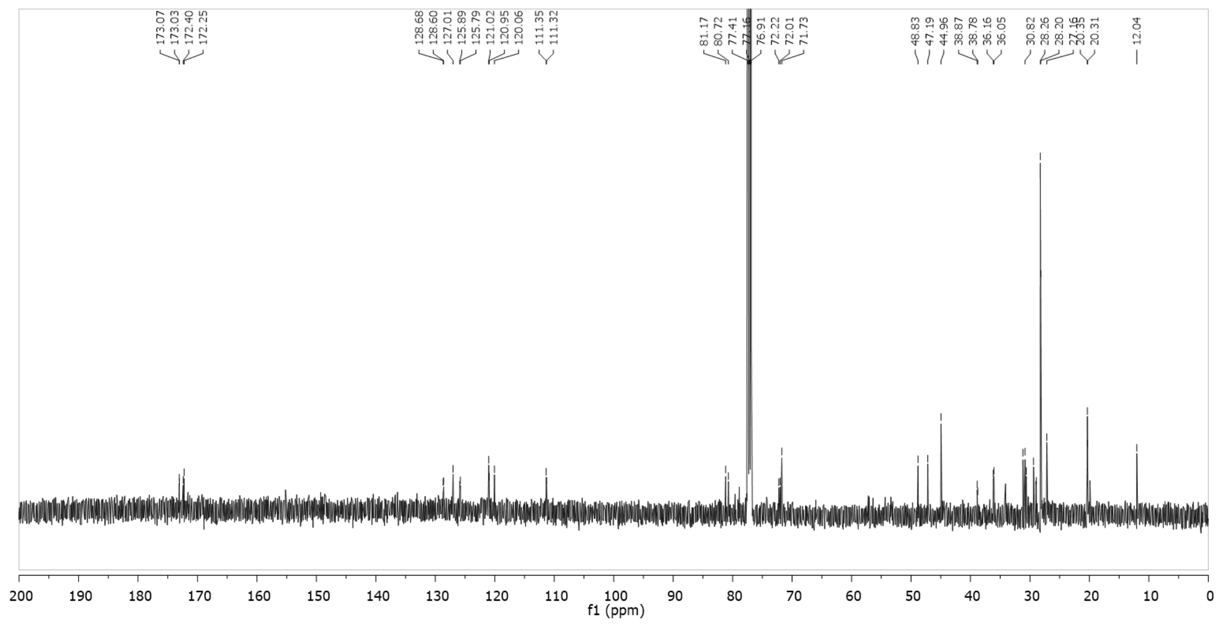

Analytical RP-HPLC-MS (I)

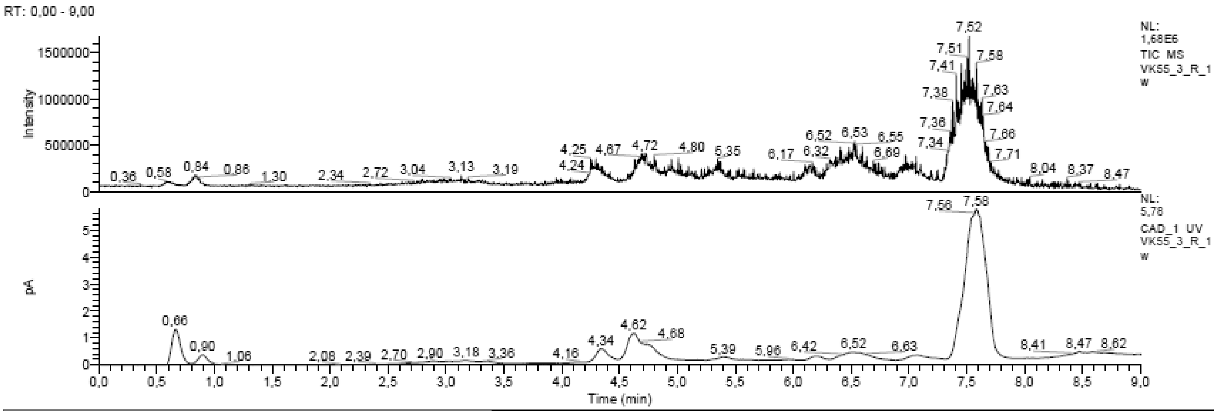

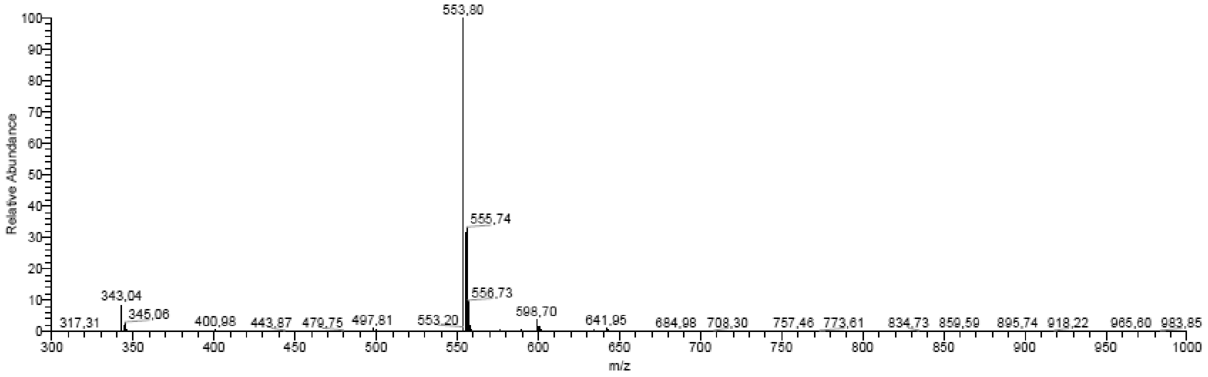

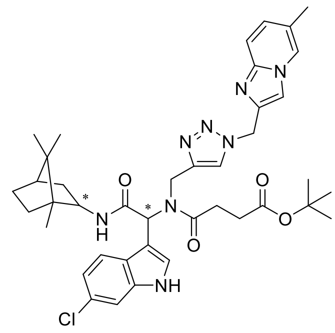

9

^{1}H NMR

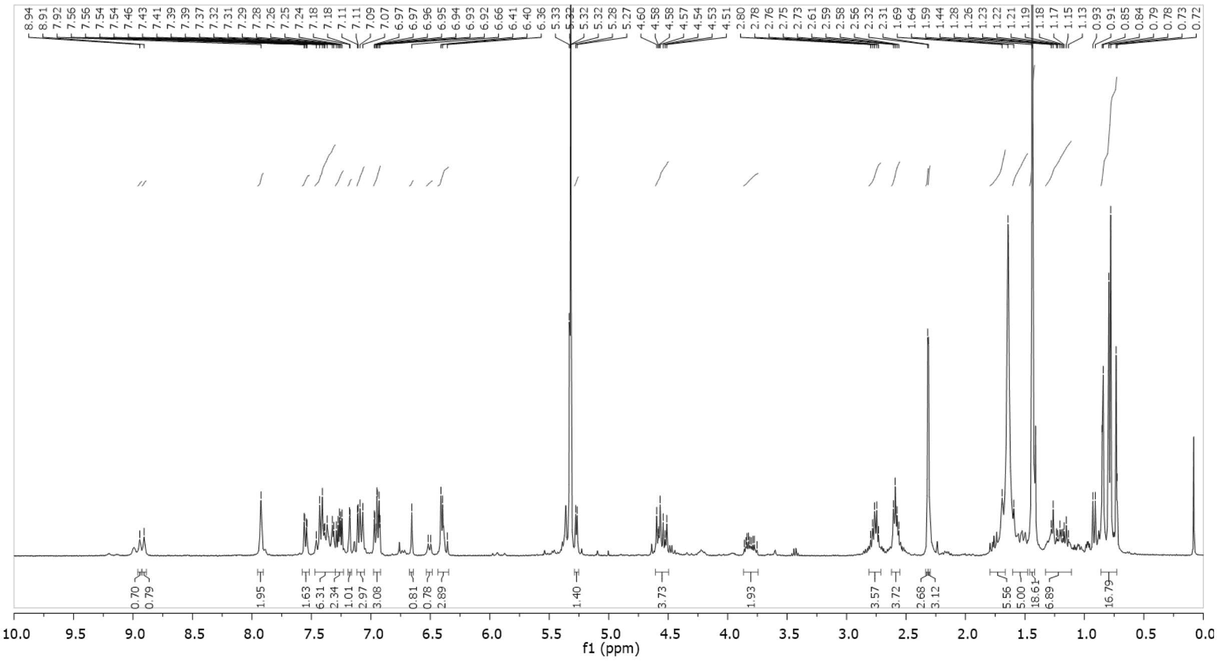

^{13}C NMR

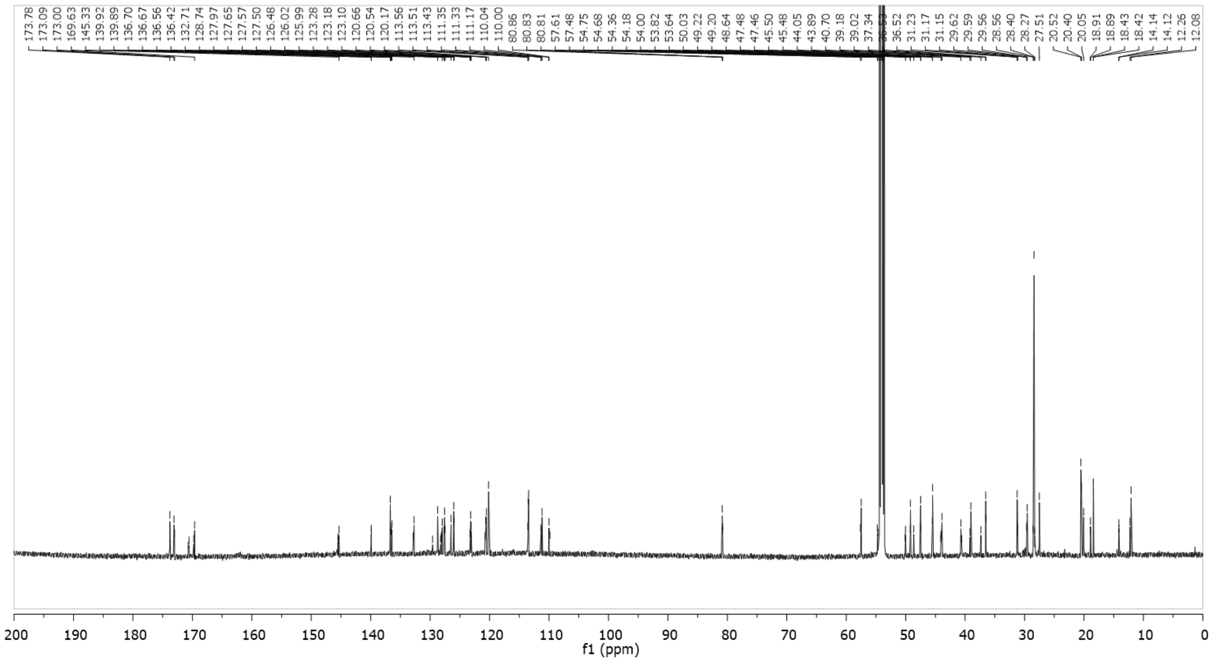

Analytical RP-HPLC-MS (I)

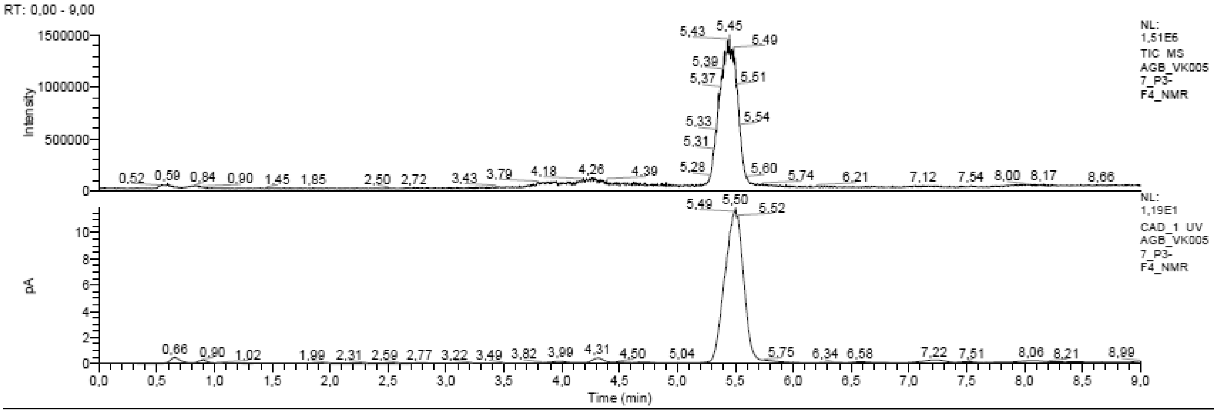

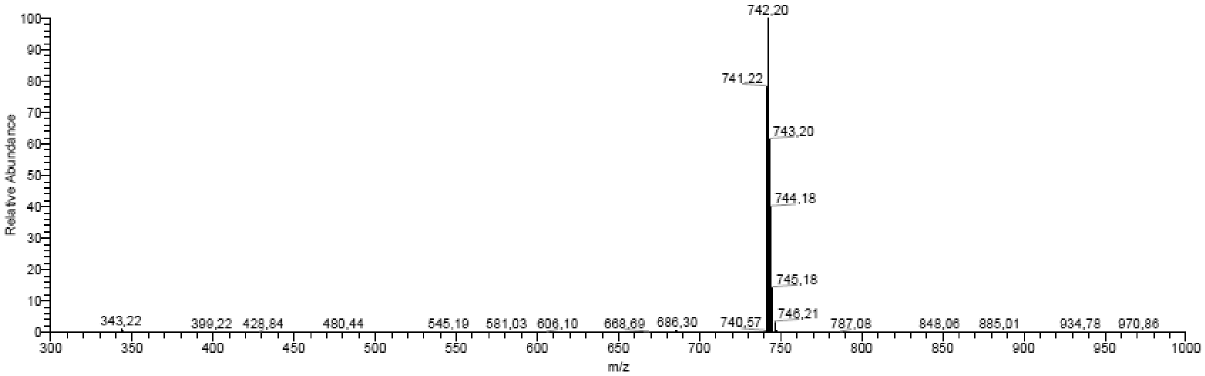

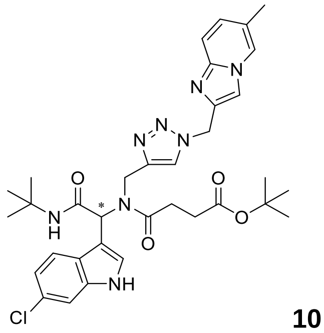

10

^{1}H NMR

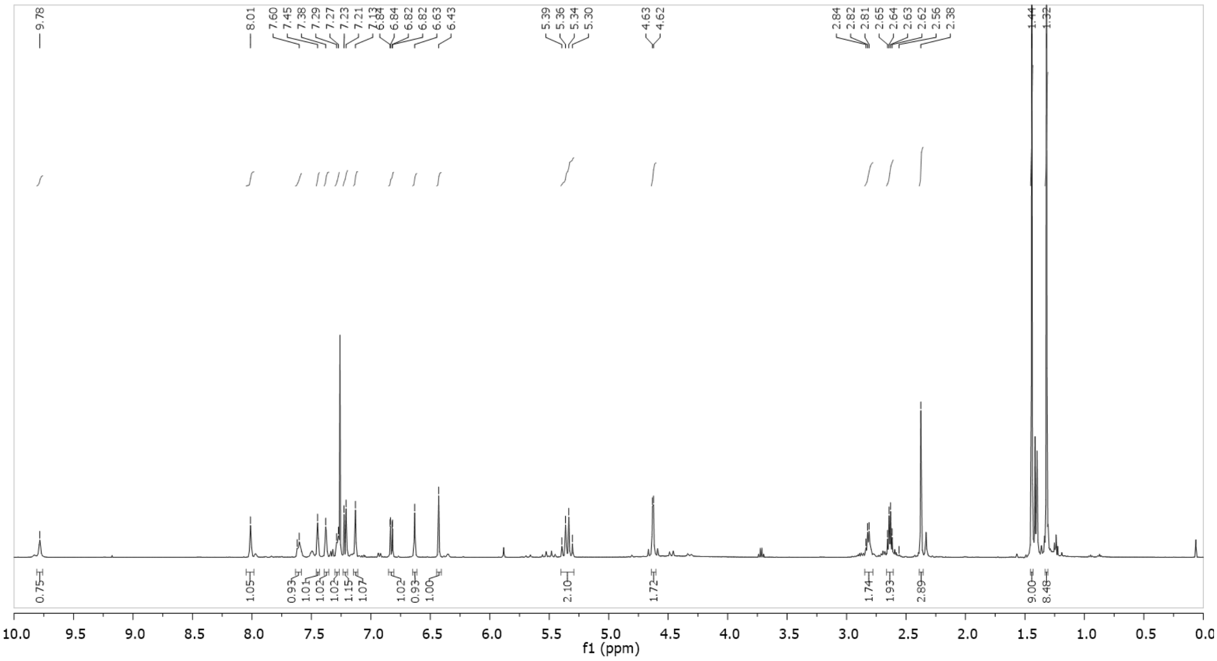

^{13}C NMR

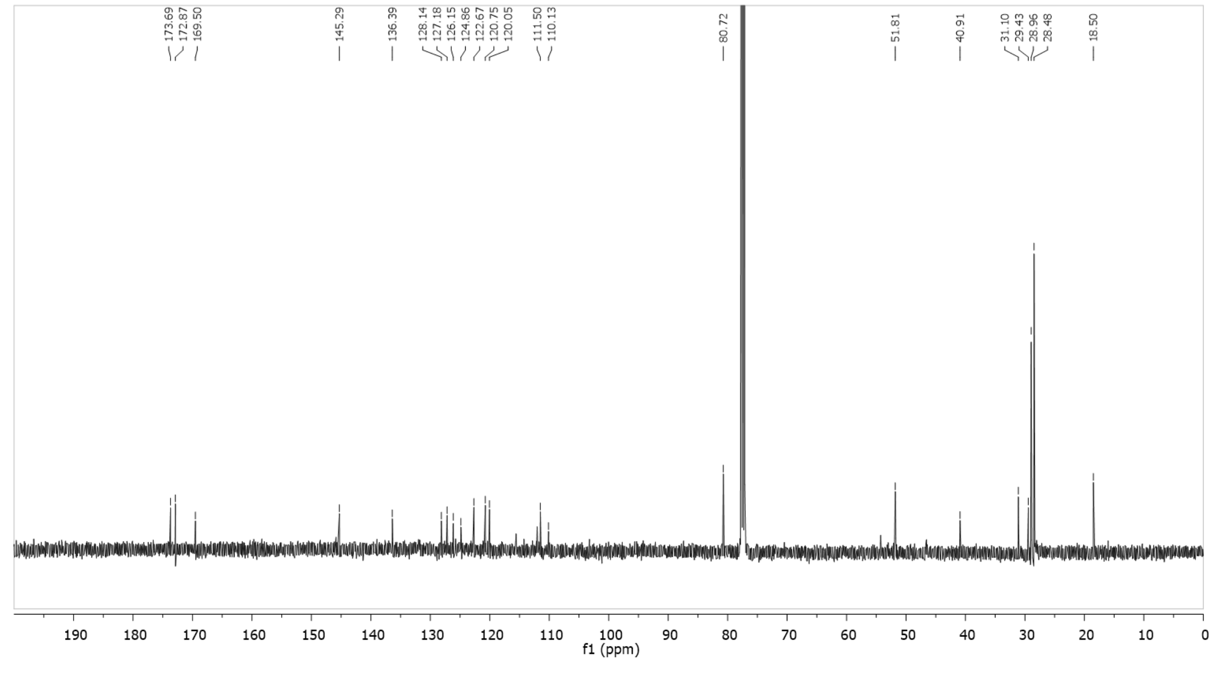

Analytical RP-HPLC-MS (I)

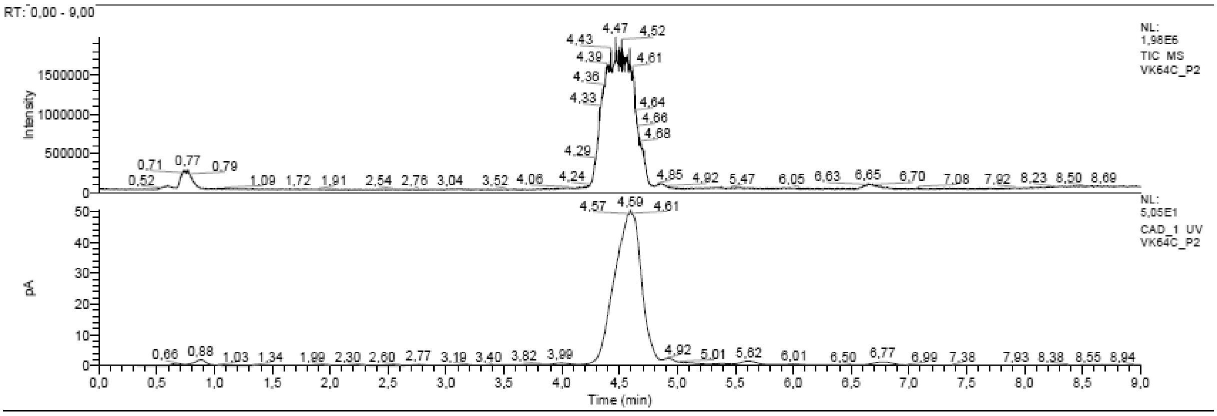

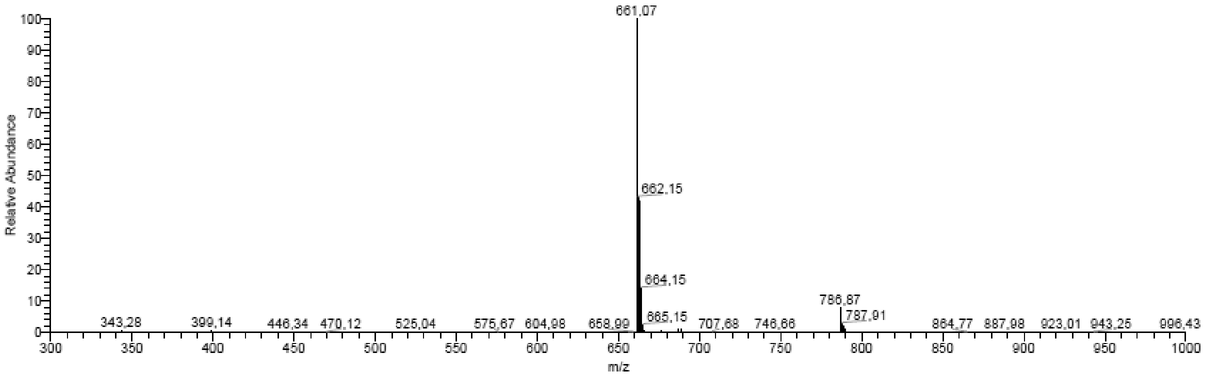

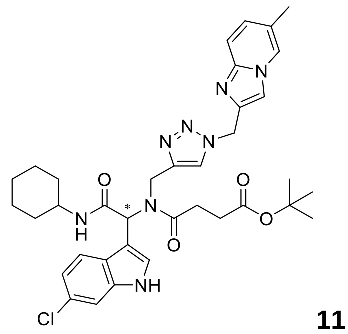

11

^{1}H NMR

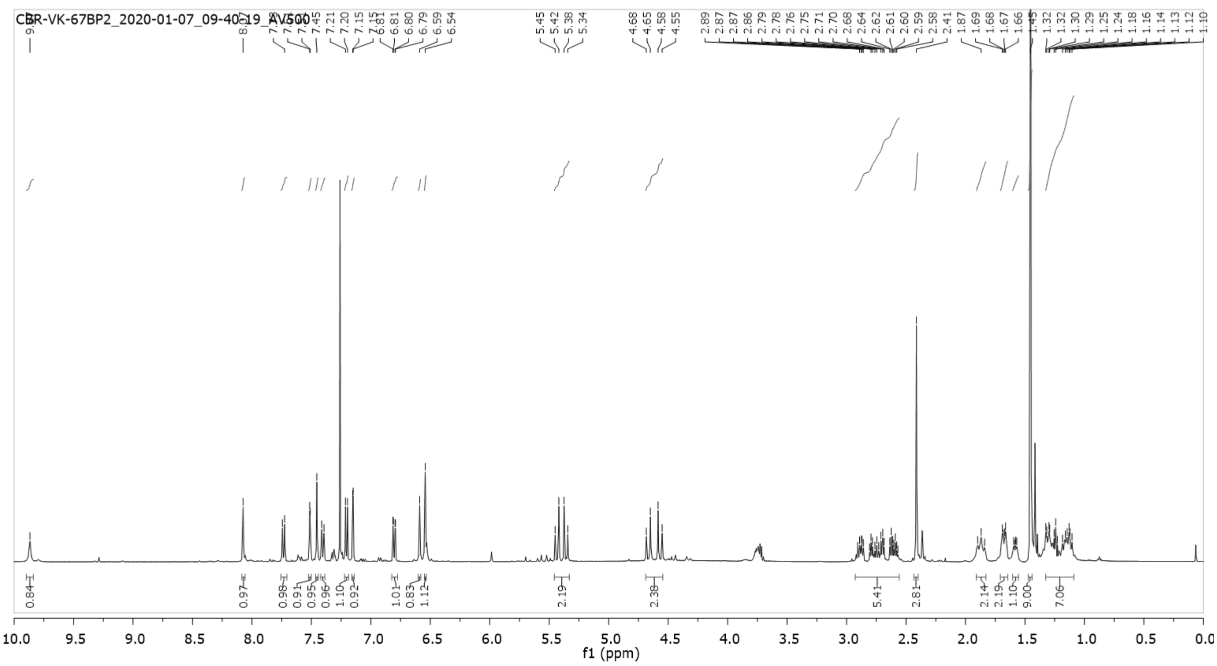

^{13}C NMR

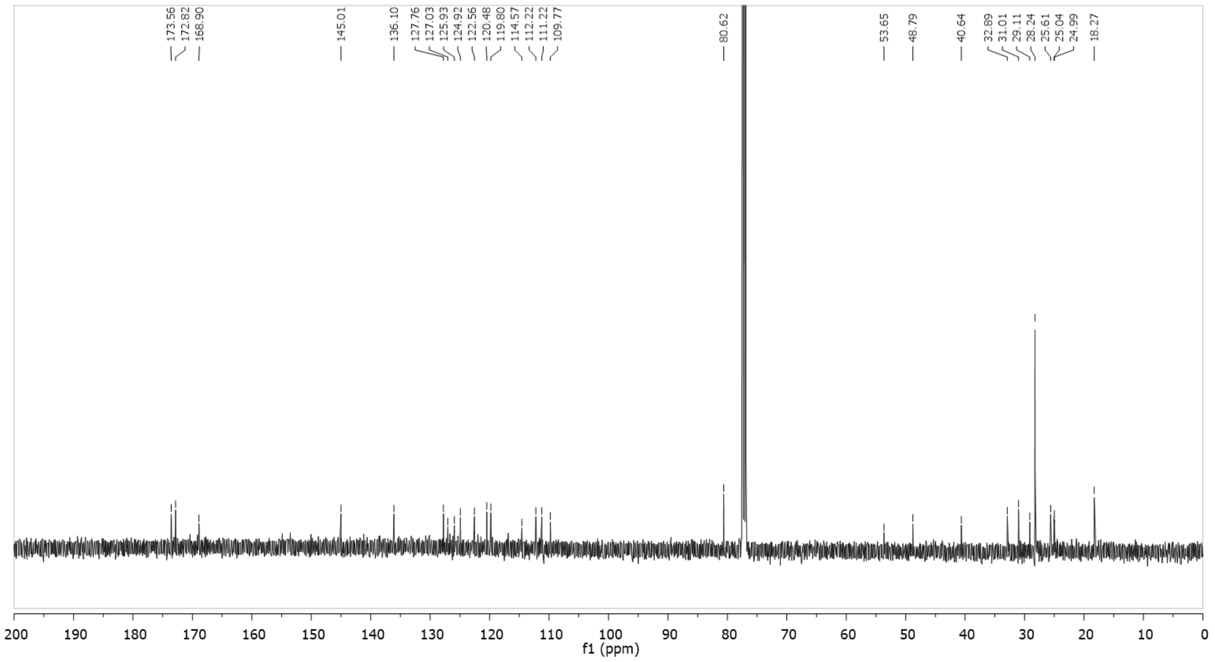

Analytical RP-HPLC-MS (I)

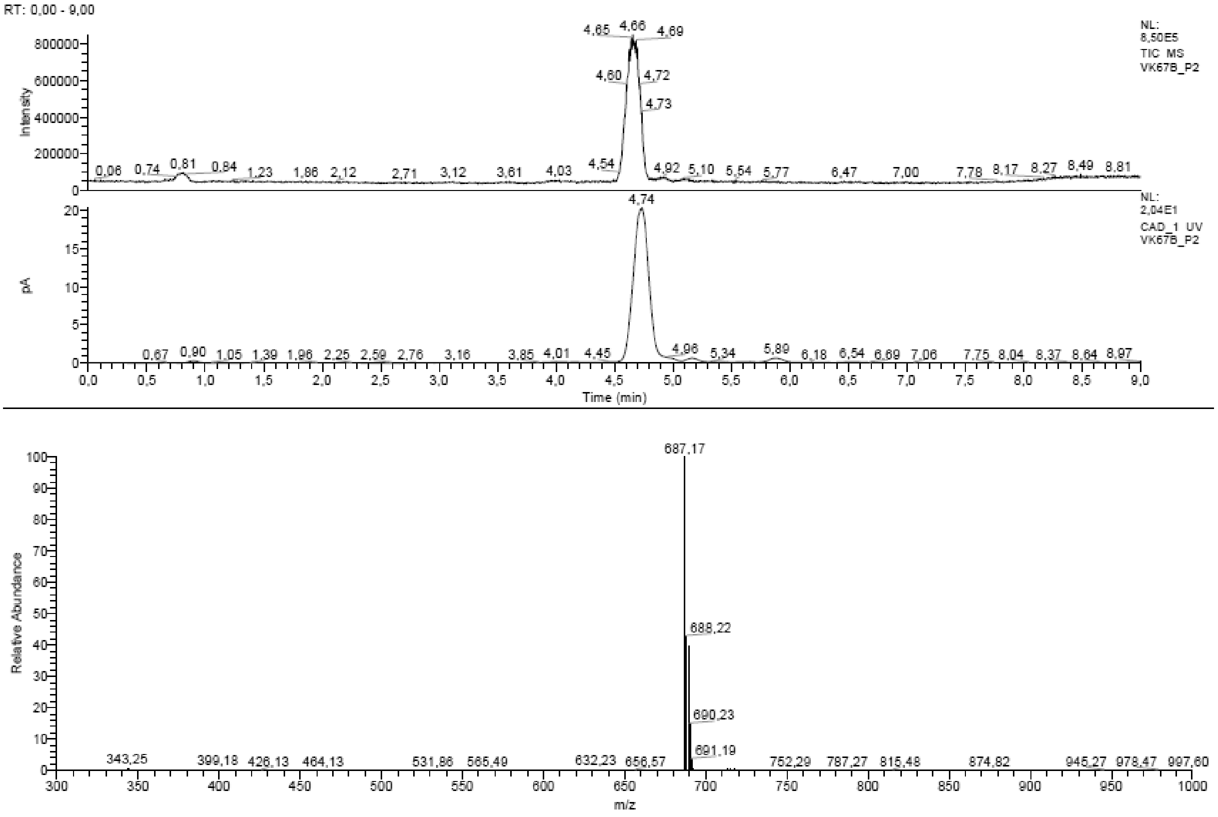

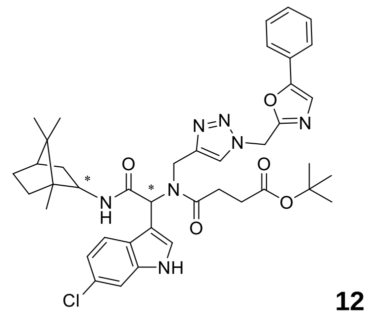

12

^{1}H NMR

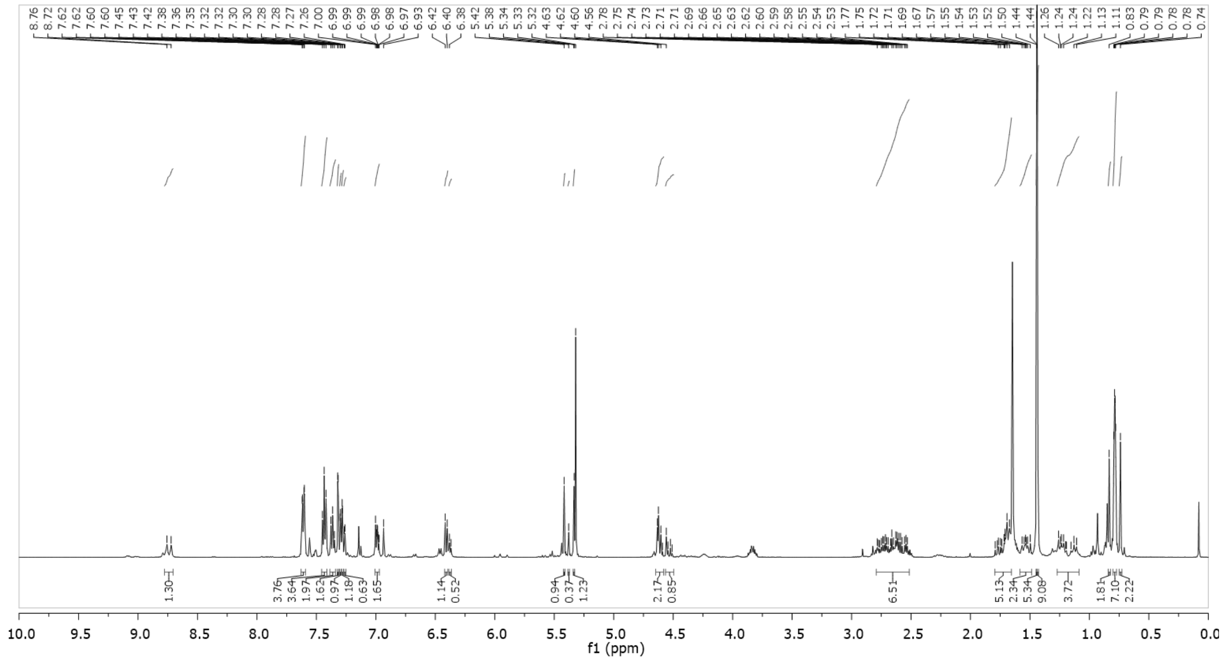

^{13}C NMR

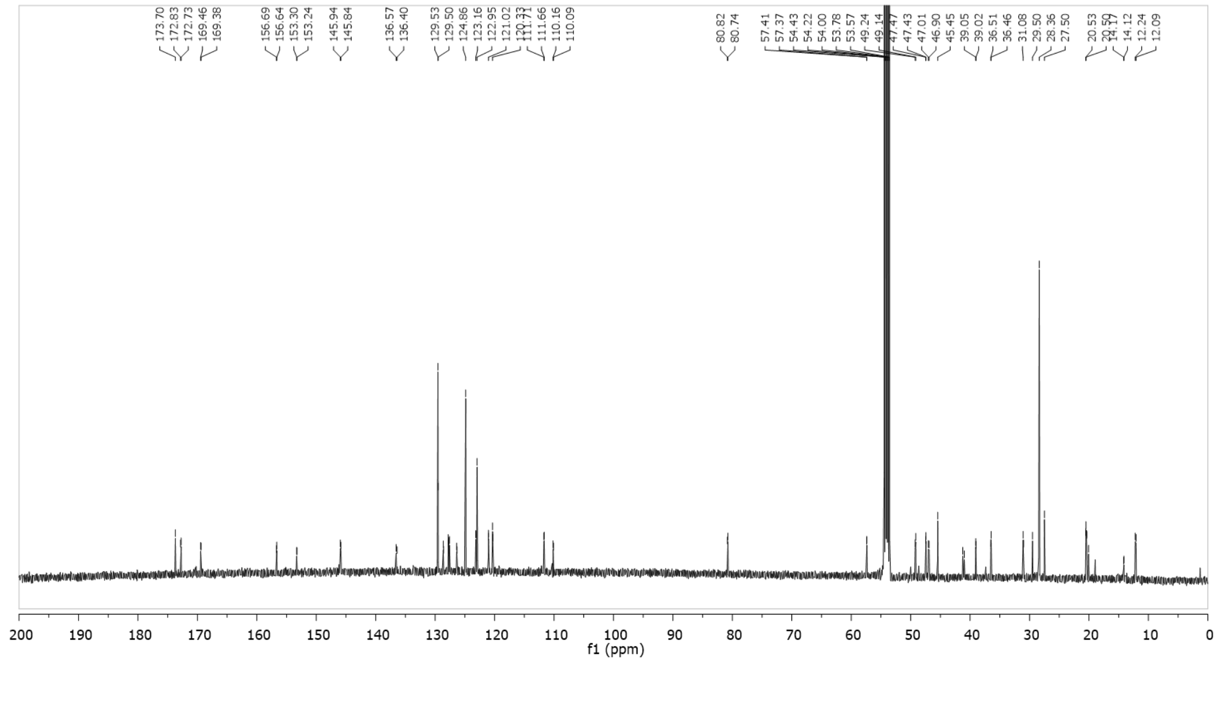

Analytical RP-HPLC-MS (II)

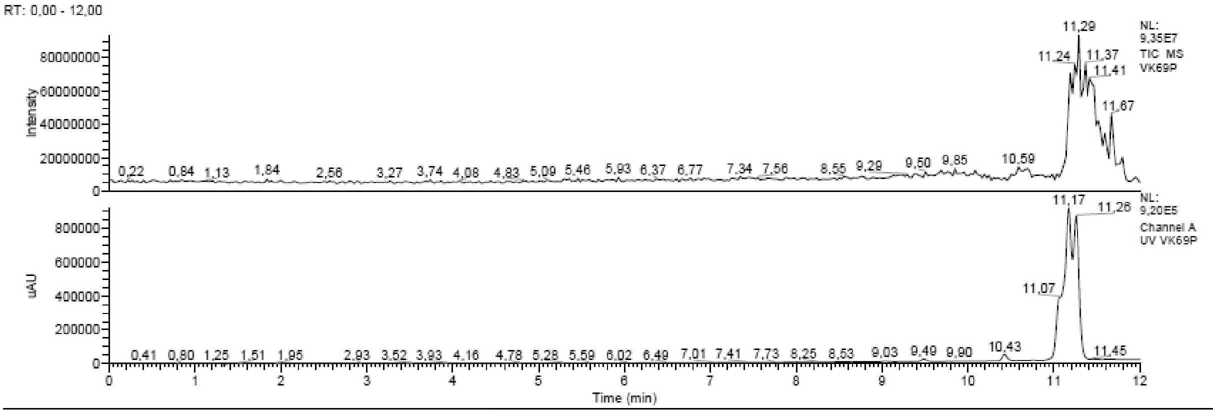

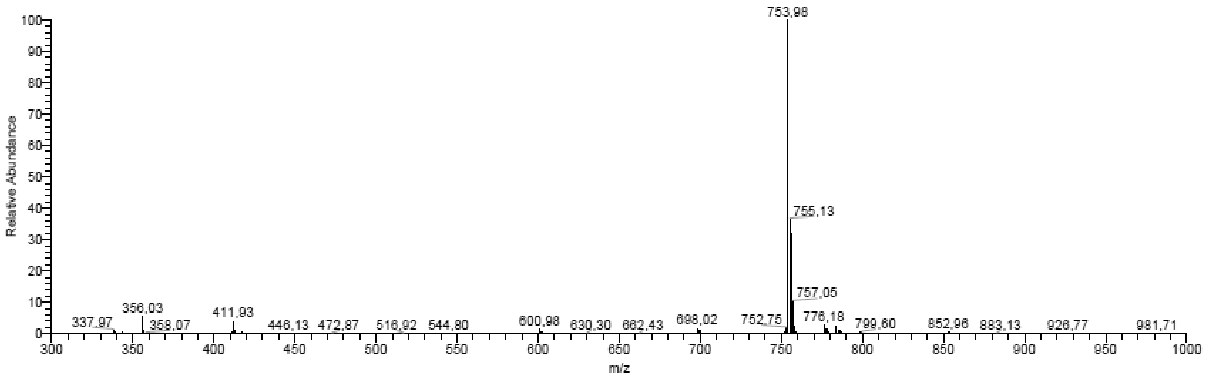

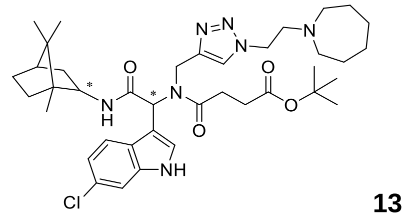

13

^{1}H NMR

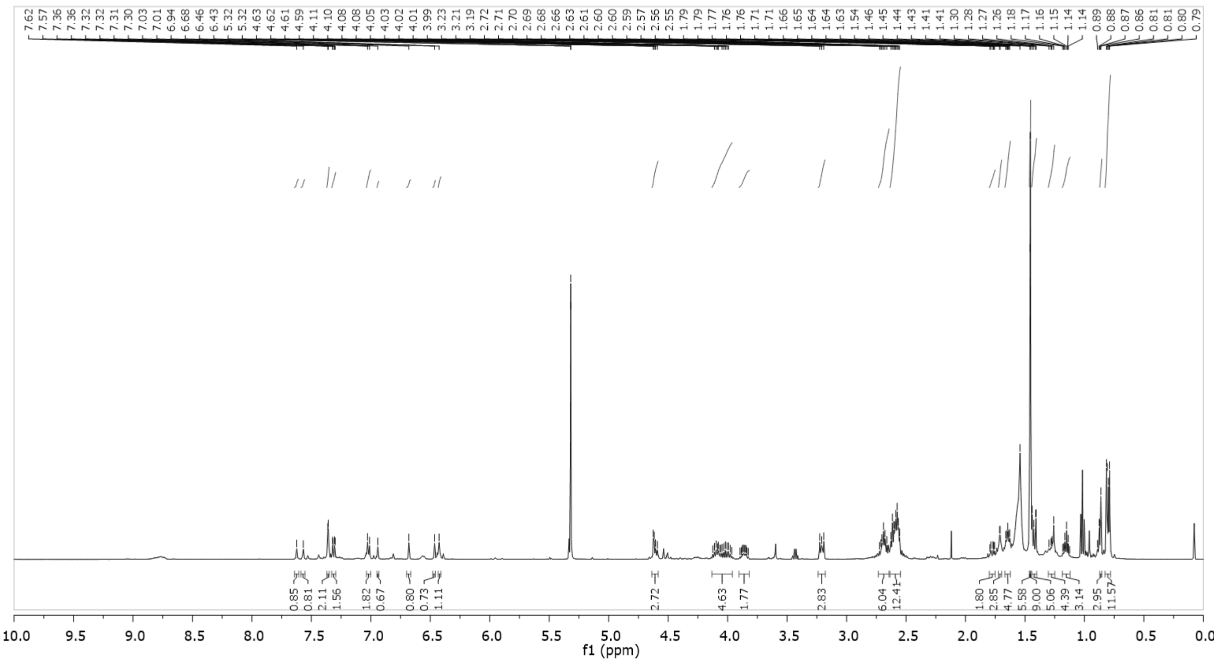

^{13}C NMR

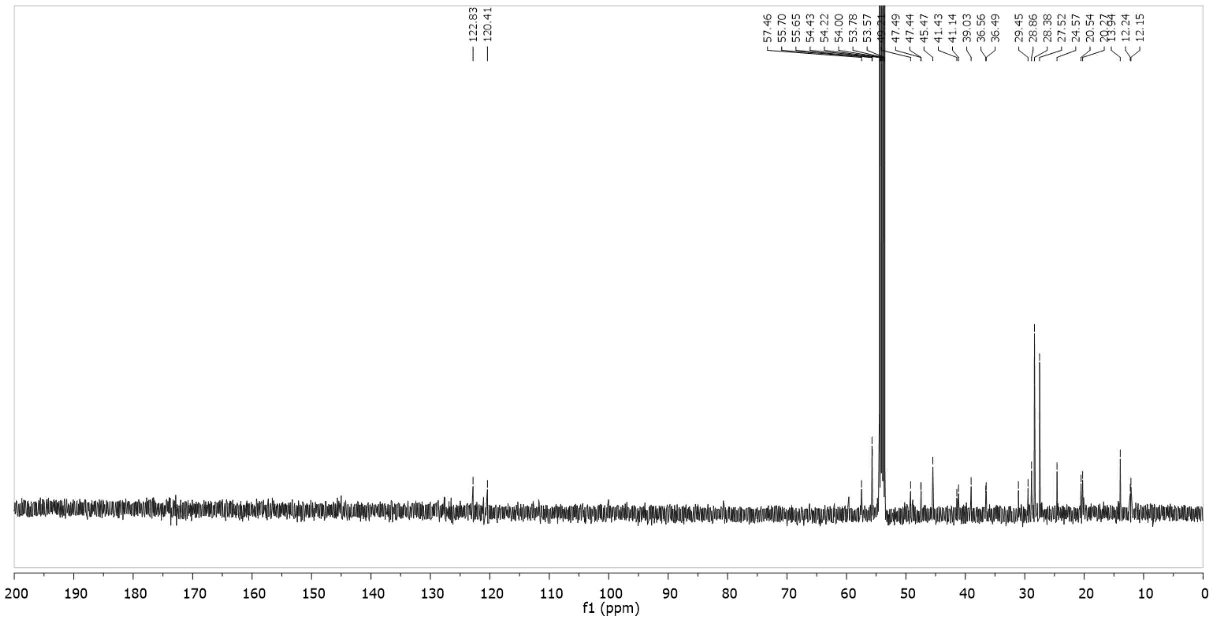

Analytical RP-HPLC-MS (II)

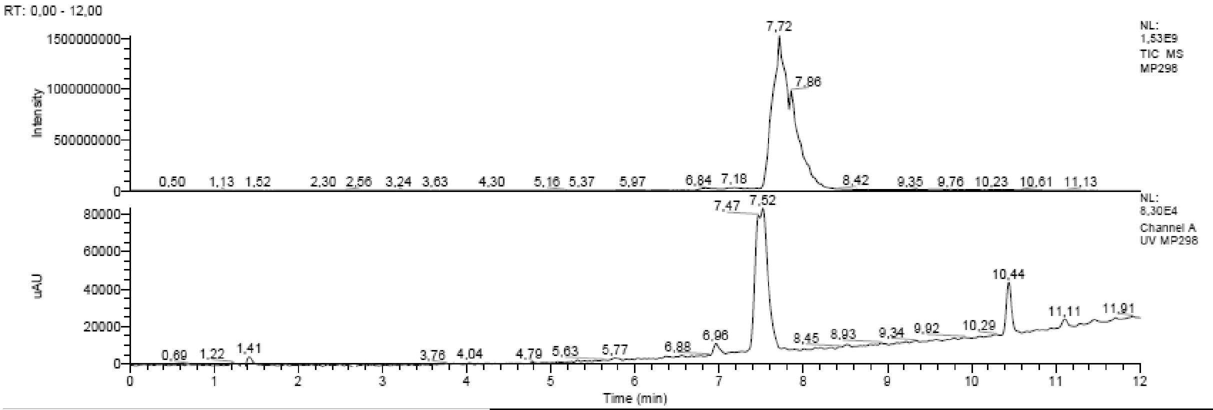

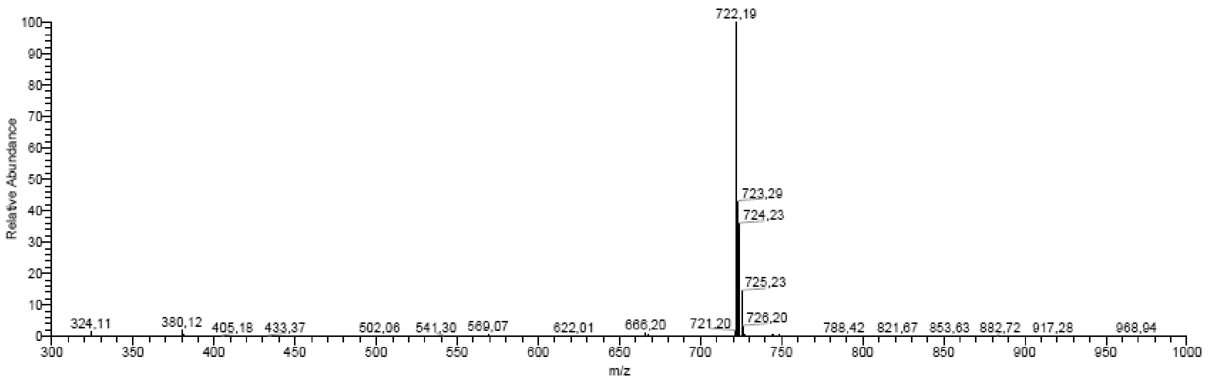

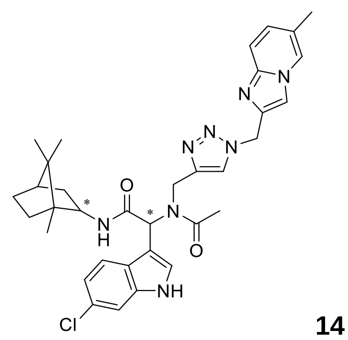

14

^{1}H NMR

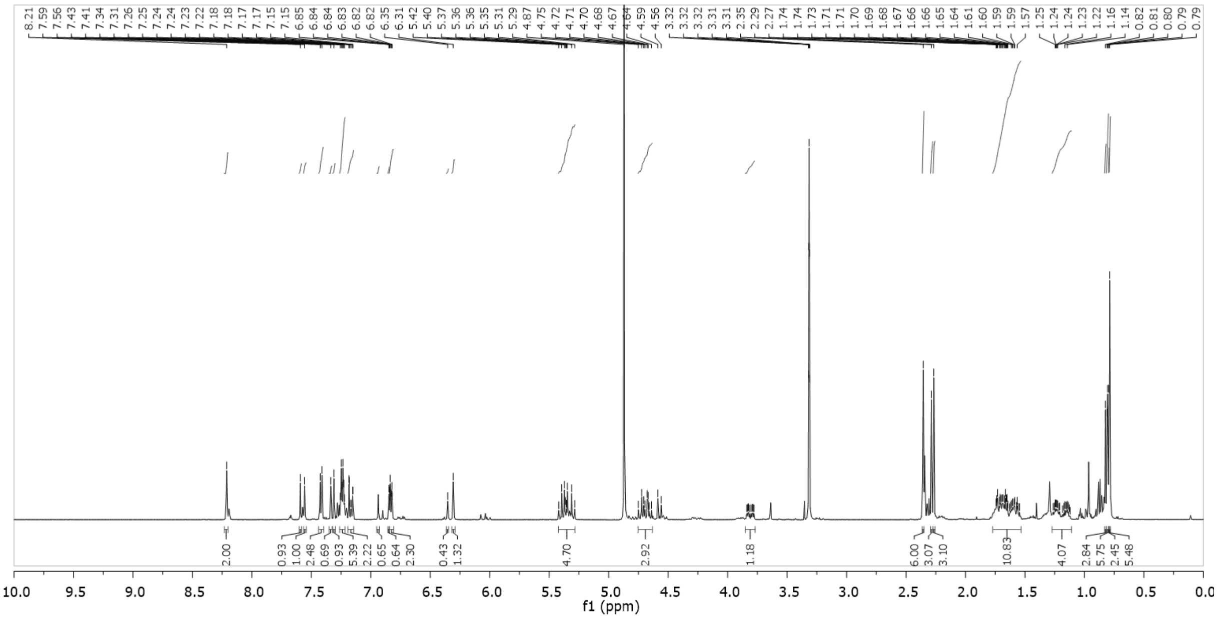

^{13}C NMR

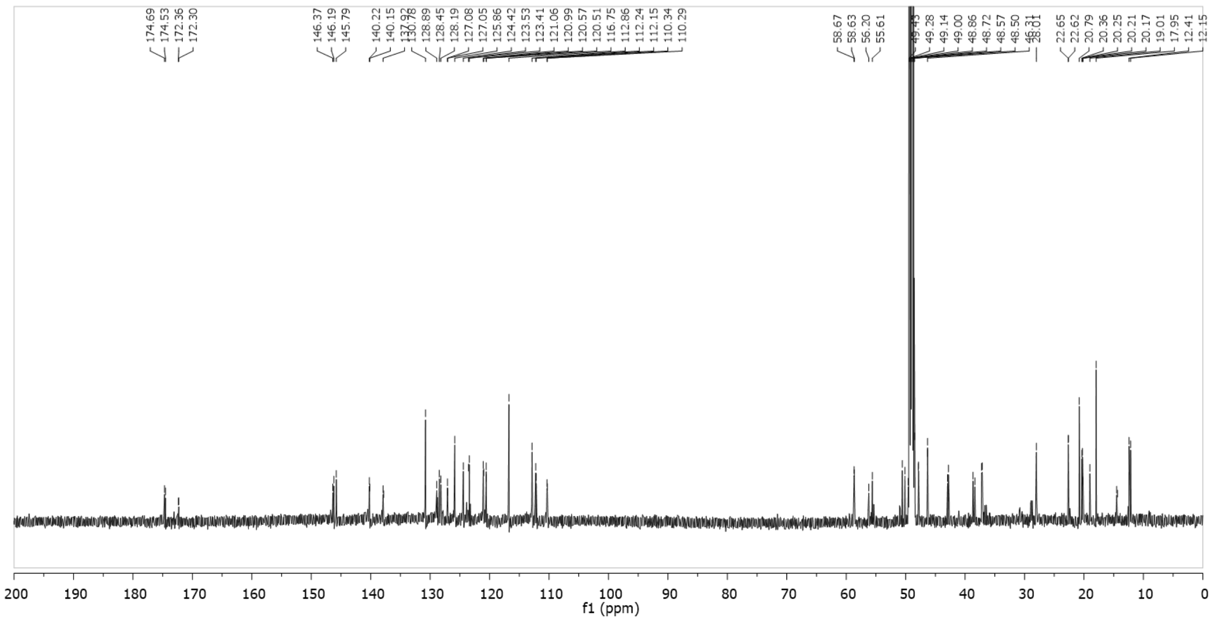

Analytical RP-HPLC-MS (I)

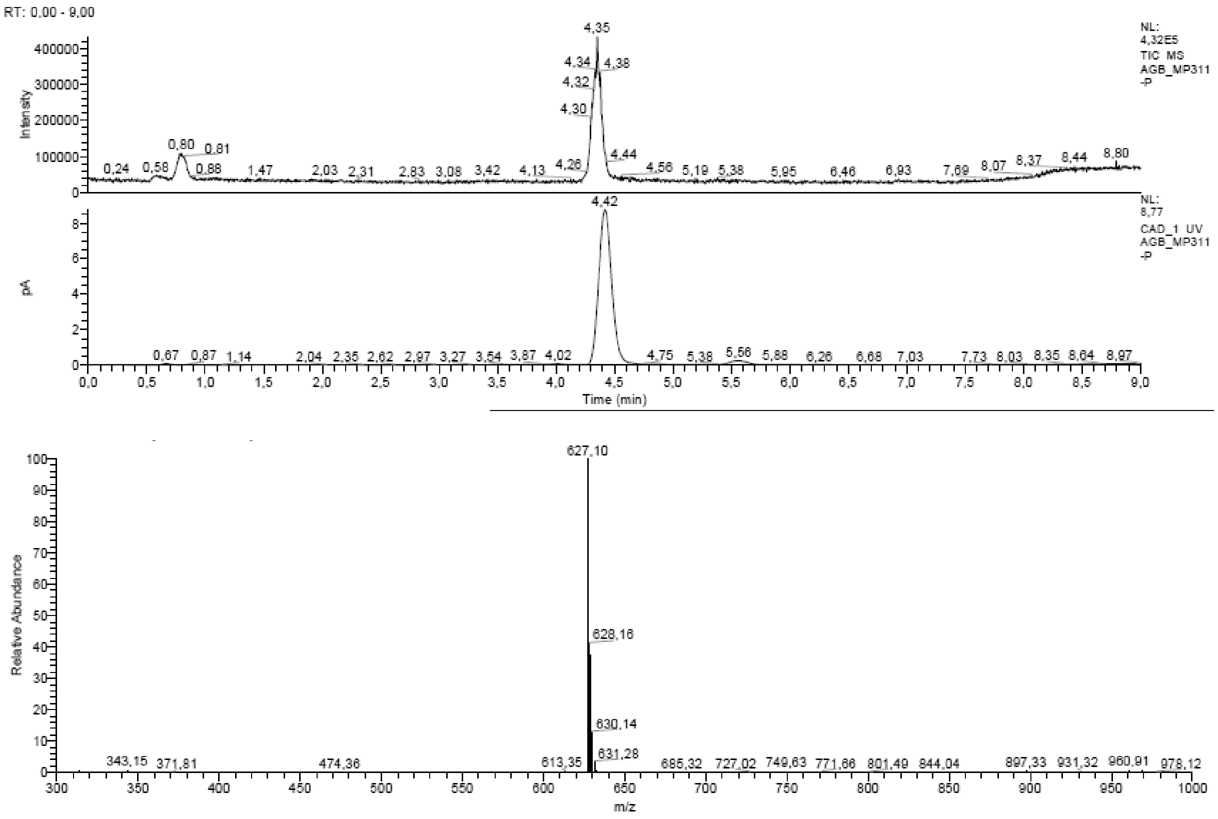

MS spectrum and HPLC chromatogram for YAP peptide

LC-MS Thermo FLEET MS spectrum

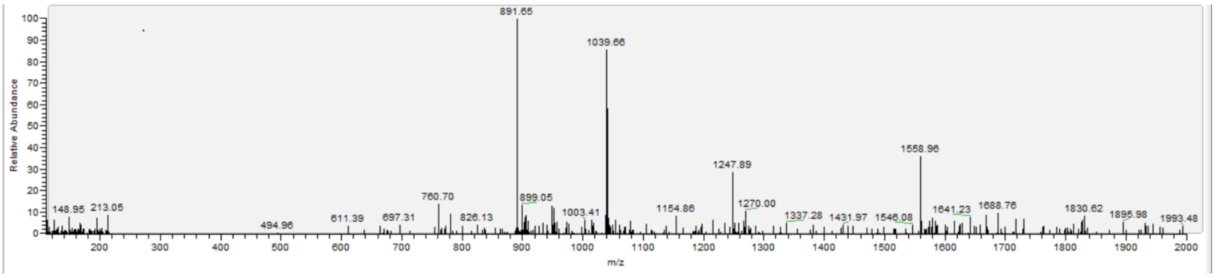

HPLC chromatogram

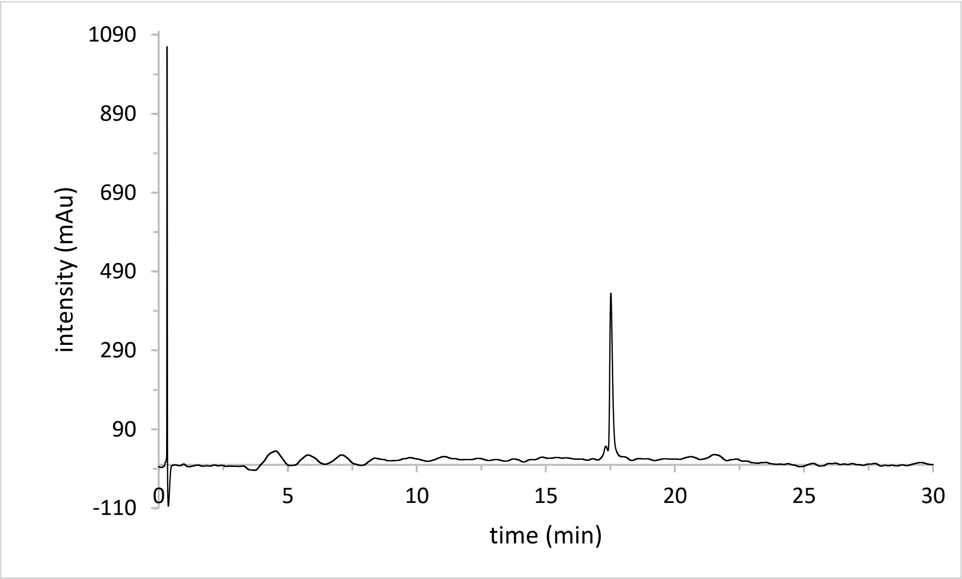

Supplementary References

[1] R Core Team (2019). R: A language and environment for statistical computing. R Foundation for Statistical Computing, Vienna, Austria. URL https://www.R-project.org/.

[2] S. Wu, X. Lei, E. Fan, Z. Sun, *Org. Lett.* **2018**, *20*, 522-525.

[3] S. Wu, J. Huang, S. Gazzarrini, S. He, L. Chen, J. Li, L. Xing, C. Li, L. Chen, C. G. Neochoritis, G. P. Liao, H. Zhou, A. Dömling, A. Moroni, W. Wang, *ChemMedChem* **2015**, *10*, 1837-1845.

[4] H. Cao, H. Liu, A. Dömling, *Chem. Eur. J.* **2010**, *16*, 12296-12298.

[5] S. Shaabani, R. Xu, M. Ahmadianmoghaddam, L. Gao, M. Stahorsky, J. Olechno, R. Ellson, M. Kossenjans, V. Helan, A. Dömling, *Green Chem.* **2019**, *21*, 225-232.

[6] C. G. Neochoritis, T. Zarganes-Tzitzikas, S. Stotani, A. Dömling, E. Herdtweck, K. Khoury, A. Dömling, *ACS Comb. Sci.* **2015**, *17*, 493-499.

[7] A. Guirado, A. Zapata, J. L. Gómez, L. Trabalón, J. Gálvez, *Tetrahedron* **1999**, *55*, 9631-9640.

[8] D. W. Carney, J. V. Truong, J. K. Sello, *J. Org. Chem.* **2011**, *76*, 10279-10285.

[9] L. W. Herman, V. Sharma, J. F. Kronauge, E. Barbarics, L. A. Herman, D. Piwnica-Worms, *J. Med. Chem.* **1995**, *38*, 2955-2963.

[10] K. Škoch, I. Císařová, P. Štěpnička, *Chem. Eur. J.* **2018**, *24*, 13788-13791.

[11] Y. Kitano, T. Manoda, T. Miura, K. Chiba, M. Tada, *Synthesis* **2006**, *2006*, 405-410.

[12] (a) R. Obrecht, R. Herrmann, I. Ugi, *Synthesis (Stuttgart)* **1985**, 400-402; (b) G. Skorna, I. Ugi, *Angew. Chem. Int. Ed. Engl.* **1977**, *16*, 259-260; (c) I. Ugi, U. Fetzer, U. Eholzer, H. Knupfer, K. Offermann, *Angew. Chem. Int. Ed. Engl.* **1965**, *4*, 472-484.

[13] (a) G. W. Gokel, R. P. Widera, W. P. Weber, *Org. Synth.* **2003**, *55*, 96-96; (b) W. P. Weber, G. W. Gokel, I. K. Ugi, *Angew. Chem. Int. Ed. Engl.* **1972**, *11*, 530-531.

[14] P. R. Gerber, K. Müller, *J. Comput. Aided Mol. Des.***1995**, *9,* 251-268.

[15] P.H. Kussie, S.Gorina, V. Marechal, B. Elenbaas, J. Moreau, A. J. Levine, N. P. Pavletich, *Science* **1996**, *274*, 948-953.

[16] H. Karatas, A.V. Pobbati, M. Akbarzadeh, H. Adihou, G. Hahne, C. Kang, S. M. Guéret, S. Sievers, A. Pahl, M. Metz, L. Dötsch, S. Zinken, C. Nowak, S.Thavam, A. Friese, E. Yihui Ng, W. Hong, H. Waldmann. Discovery of Covalent Inhibitors Targeting the Transcriptional Enhanced Associate Domain (TEAD) Central Pocket. Unpublished.
